# A multi-omic atlas of human embryonic skeletal development

**DOI:** 10.1038/s41586-024-08189-z

**Published:** 2024-11-20

**Authors:** Ken To, Lijiang Fei, J. Patrick Pett, Kenny Roberts, Raphael Blain, Krzysztof Polański, Tong Li, Nadav Yayon, Peng He, Chuan Xu, James Cranley, Madelyn Moy, Ruoyan Li, Kazumasa Kanemaru, Ni Huang, Stathis Megas, Laura Richardson, Rakesh Kapuge, Shani Perera, Elizabeth Tuck, Anna Wilbrey-Clark, Ilaria Mulas, Fani Memi, Batuhan Cakir, Alexander V. Predeus, David Horsfall, Simon Murray, Martin Prete, Pavel Mazin, Xiaoling He, Kerstin B. Meyer, Muzlifah Haniffa, Roger A. Barker, Omer Bayraktar, Alain Chédotal, Christopher D. Buckley, Sarah A. Teichmann

**Affiliations:** 1 Wellcome Sanger Institute, Wellcome Genome Campus, Hinxton, UK; 2 Department of Surgery, University of Cambridge, Cambridge, UK; 3 Sorbonne Université, INSERM, CNRS, Institut de la Vision, Paris, France; 4 European Molecular Biology Laboratory, European Bioinformatics Institute (EMBL-EBI), Wellcome Genome Campus, Cambridge, UK; 5 Department of Pathology, University of California, San Francisco, San Francisco, CA USA; 6 Department of Medicine, University of Cambridge, Cambridge, UK; 7 Cambridge Centre for AI in Medicine, Department of Applied Mathematics and Theoretical Physics, Cambridge, UK; 8 John van Geest Centre for Brain Repair, Department of Clinical Neurosciences, University of Cambridge, Cambridge, UK; 9 Cambridge Stem Cell Institute, University of Cambridge, Jeffrey Cheah Biomedical Centre, Cambridge Biomedical Campus, Cambridge, UK; 10 Newcastle University, Biosciences Institute, Newcastle University, Newcastle upon Tyne, UK; 11 Department of Dermatology and NIHR Newcastle Biomedical Research Centre, Newcastle Hospitals NHS Foundation Trust, Newcastle upon Tyne, UK; 12 Institut de Pathologie, Groupe Hospitalier Est, Hospices Civils de Lyon, Lyon, France; 13 University Claude Bernard Lyon 1, MeLiS, CNRS UMR5284, INSERM U1314, Lyon, France; 14 Kennedy Institute of Rheumatology, University of Oxford, Oxford, UK; 15 CIFAR Macmillan Multi-scale Human Programme, CIFAR, Toronto, Canada

**Keywords:** Bone development, RNA sequencing

## Abstract

Human embryonic bone and joint formation is determined by coordinated differentiation of progenitors in the nascent skeleton. The cell states, epigenetic processes and key regulatory factors that underlie lineage commitment of these cells remain elusive. Here we applied paired transcriptional and epigenetic profiling of approximately 336,000 nucleus droplets and spatial transcriptomics to establish a multi-omic atlas of human embryonic joint and cranium development between 5 and 11 weeks after conception. Using combined modelling of transcriptional and epigenetic data, we characterized regionally distinct limb and cranial osteoprogenitor trajectories across the embryonic skeleton and further described regulatory networks that govern intramembranous and endochondral ossification. Spatial localization of cell clusters in our in situ sequencing data using a new tool, ISS-Patcher, revealed mechanisms of progenitor zonation during bone and joint formation. Through trajectory analysis, we predicted potential non-canonical cellular origins for human chondrocytes from Schwann cells. We also introduce SNP2Cell, a tool to link cell-type-specific regulatory networks to polygenic traits such as osteoarthritis. Using osteolineage trajectories characterized here, we simulated in silico perturbations of genes that cause monogenic craniosynostosis and implicate potential cell states and disease mechanisms. This work forms a detailed and dynamic regulatory atlas of bone and cartilage maturation and advances our fundamental understanding of cell-fate determination in human skeletal development.

## Abstract (web summary)

A multiomic atlas of human embryonic joint and cranium development enables detailed analysis of bone and cartilage maturation.

## Main

**Fig. 1:**
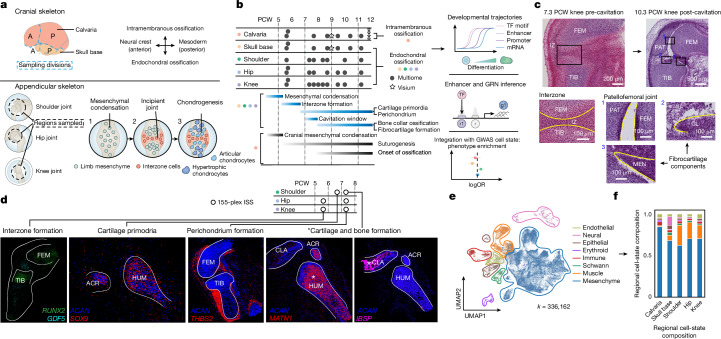
Multi-omics of human embryonic skeletal development. **a**, Anatomical sampling approach for the five main anatomical regions. Top, origin cells within the cranium are determined by the anterior (A)–posterior (P) axis. We sampled according to these canonical divisions. Incisions were made as shown by the blue dotted lines (top). Appendicular regions sampled are shown within the black dotted lines (bottom). Bottom, the diagram portrays (1) initial condensation of the mesenchyme, (2) joint-site determination by interzone cells in the incipient joint, and (3) chondrogenesis occurring within the region of the joint. **b**, Sample donor (*n *= 12) overview across age and anatomical regions sampled; atlasing modalities are represented in the legend. The timeline of morphological events and changes across the timeline sampled (bottom left) are shown; key analyses undertaken with Multiome data generated from this work are also displayed (right). g1, gene; r, region; TF, transcription factor. **c**, Haematoxylin and eosin staining of tissue sections of the knee joint to illustrate features before and after cavitation. The interzone (IZ) is present before cavitation and this is separated by the joint space following cavitation with emergence of soft-tissue structures including ligaments. CL, cruciate ligament; FEM, femur; MEN, meniscus; PAT, patella; TIB, tibia. Images are representative of sections from two donors. **d**, Marker genes detected by ISS probes applied to the tissue samples indicated in the schematic. The white lines outline regions of bone. The asterisks show cartilage in the humerus (HUM) and bone in the clavicle (CLA). ACR, acromion. Images are representative of three donors. **e**, Uniform manifold approximation and projection (UMAP) embedding of the dataset using MultiVI latent variables calculated from snRNA and snATAC data. The colours indicate cell cluster compartments. **f**, Relative cell-type abundance across anatomical locations. The bar plot of the proportion of the cell cluster compartment in each anatomical region sampled shows predominance of the mesenchyme compartment across anatomical regions.

Human bone development begins between 6 and 8 weeks after conception (post-conception weeks, PCW) during the transition from embryonic to fetal stages. In the cranium, calvarial progenitors differentiate into osteoblasts through intramembranous ossification and continue to house osteoprogenitors postnatally^1,2^. In the nascent synovial joint, an interzone condensation appears in the limb bud at 5–6 PCW^3^ and forms a joint cavity between 7 and 8 PCW, varying in timing across joints, within which fibrous and ligamentous structures develop^4,5^ (Fig. 1b,c). Cartilage scaffolds form on either side of synovial joints to facilitate development of the body plane until they are replaced by bone tissue as endochondral ossification ensues from 8 PCW^6,7^. These regionally distinct modes of ossification govern osteogenesis throughout the human skeleton. To our knowledge, the cellular basis by which they form and mature remain incompletely described in human development at single-cell resolution. To address this, we applied single-nucleus paired RNA (snRNA) and assay for transposase-accessible chromatin (snATAC) sequencing (seq), and spatial methods, to decipher the regulatory landscape that mediates maturation of the distinct bone-forming and joint-forming niches in the embryonic cranium and limbs from 5 to 11 PCW. Through this, we uncovered previously undescribed cellular diversity in the osteogenic and chondrogenic lineages. We developed ISS-Patcher, a tool to impute cell labels from the droplet data on our high-resolution 155-plex in situ sequencing (ISS) datasets, which facilitated insights into spatially defined niches within the embryonic synovial joint. Applying OrganAxis^8^, a new spatial transcriptomics annotation tool, we also define the spatial trajectory of the developing cranial bone. We characterized novel cell states of the craniofacial region and additionally delineated processes of human Schwann cell and fibroblast development. Our resource and new computational toolset, including SNP2Cell, enabled predictions of the mechanisms of developmental conditions, such as craniosynostosis^9–11^, and allowed association of gene-regulatory networks (GRNs) in region-specific mesenchymal clusters to ageing diseases of the human skeleton, such as osteoarthritis^12,13^.

## Cellular taxonomy of joint development

**Extended Data Fig. 1:**
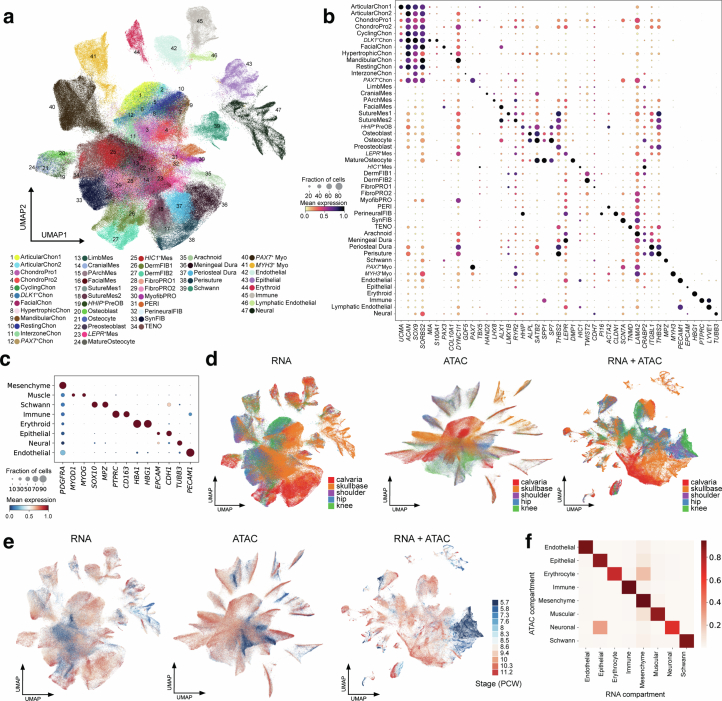
Droplet dataset overview. **a**. UMAP-embedding of RNA droplets with broad cell cluster labels. **b**. Dotplot with marker genes for each cluster. **c**. Dotplot with marker genes for each cell compartment. **d**. Skeletal region metadata displayed on RNA (left), ATAC (middle) and co-embedding (Right). **e**. Developmental stage metadata (/PCW) displayed on RNA (left), ATAC (middle) and co-embedding (Right). **f**. Heatmap of concordance of cell compartment labels across RNA and ATAC modalities, scale bar shows the proportion of intersected cells across RNA and ATAC compartment (scaled by row).

**Extended Data Fig. 2:**
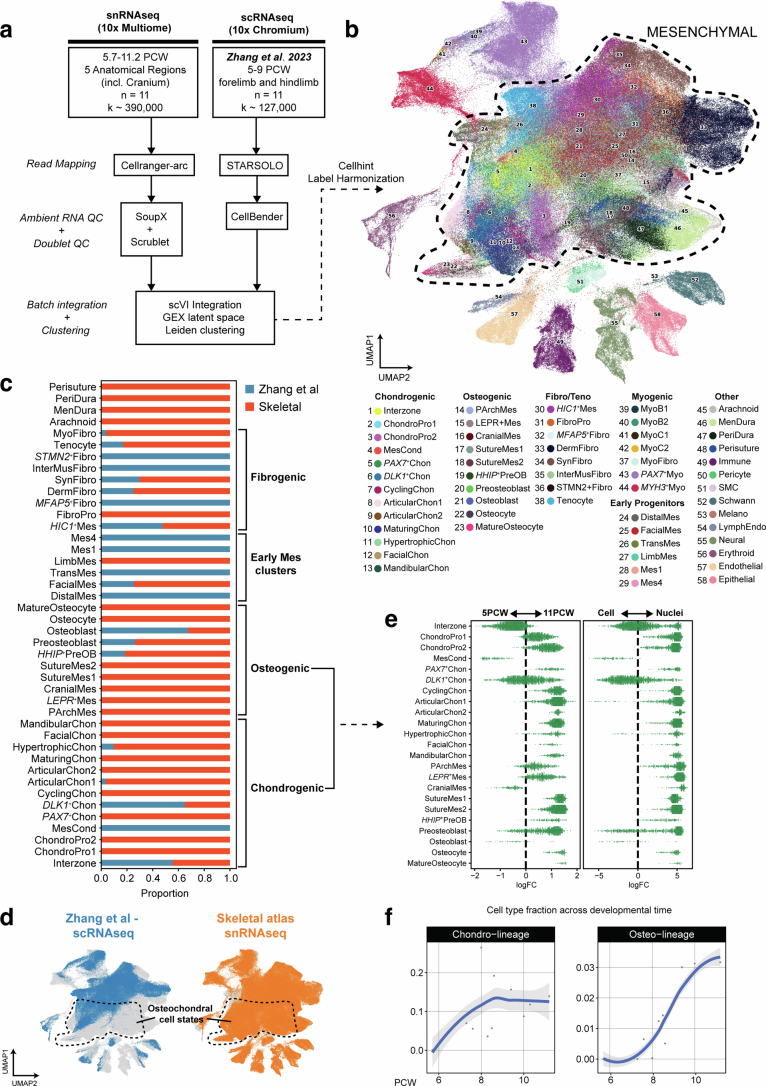
Integrative analysis of skeletal atlas with published limb atlas data. **a**. Computational workflow for integrating Zhang et al. data by remapping to a common reference, followed by quality control and batch integration using scVI and CellHint (see Methods). **b**. UMAP of the integrated datasets with harmonised cluster labels. **c**. Barplot of proportion of cell type from each of the two studies. **d**. UMAP of the integrated datasets showing droplets originating from each dataset. Dotted line shows regions of osteochondral cell states. **e**. MILO analysis of differential abundance of each cell type across age (PCW) and modality. **f**. Line graph of cell type fraction across developmental time (/PCW) in the chondrogenesis and osteogenesis lineages, showing enrichment of both lineages toward the latter parts of the first trimester. Data: mean with 95% confidence interval, n = 12 sample of donors.

**Extended Data Fig. 3:**
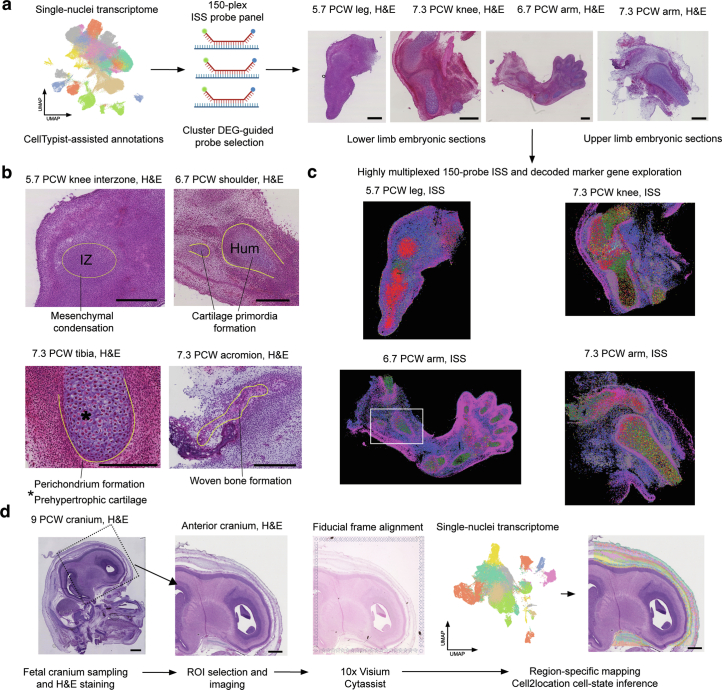
Spatial transcriptomics analysis and workflow. **a**. Workflow schematic showing UMAP-embedding of RNA droplets of the appendicular joints manually annotated informed by Celltypist utilising Zhang et al., 2023 labels. Per-cluster DEG from these were used to design a 150-plex probe panel, subsequently applied to sectioned samples of 5-7 PCW synovial joints (Right). Scale bars = 500 µm, Images are representative of three donors. **b**. Regions of cellular niches within the samples utilised for ISS imaging. Scale bars = 500 µm, Images are representative of three donors. **c**. Visualisation of probe distribution on ISS imaging data applied to histological sections in a, white box shows nascent shoulder joint in 6.7 PCW arm. **d**. Workflow schematic for 10x Visium Cytassist applied to 9 PCW cranium, Scale bars = 2000 µm.

**Extended Data Fig. 4:**
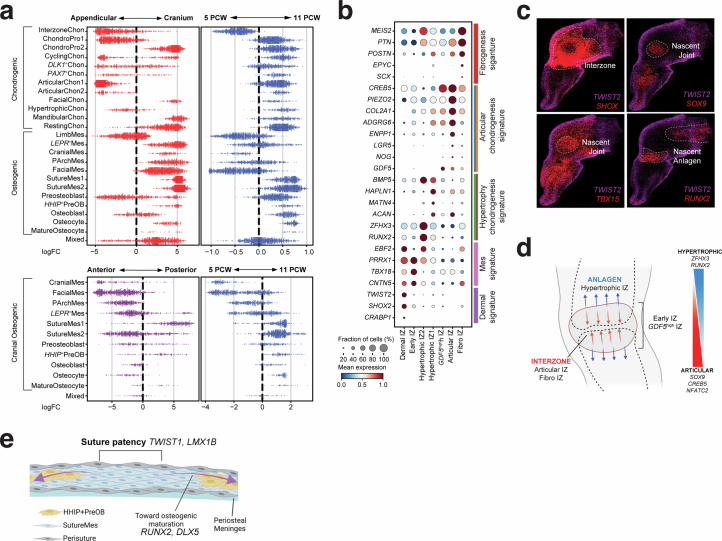
Cell states in the limb and cranial joints. **a**. Differential abundance (MILO, see Methods) of cell states across lineages against multiple comparators. Top: Osteogenesis and chondrogenesis clusters across region and PCW. Bottom: Cranial osteogenesis subclusters across region and PCW. Mixed: other related cell types that are not included in each plot. **b**. Dotplot of normalised expression of genes associated with skeletal development within InterzoneChon subclusters (IZs). **c**. RNA-ISS of the 5.7 PCW hindlimb, showing expression patterns of select genes. **d**. Schematic of knee joint interzone formation, gradients showing genes associated with zonated hypertrophic and articular phenotypes. **e**. Schematic illustrating TF gradients across regions of the coronal suture. Schematics in panels **d**,**e** were created using BioRender (https://biorender.com).

We performed paired droplet-based snRNA-seq and snATAC-seq (10X Genomics Multiome) on the hip, knee and shoulder joints across samples from 12 donors between 5 and 11 PCW (Fig. 1a,b). For the developing cranium, which had not been previously profiled across different ages, to our knowledge, we sampled the anterior and posterior regions of the calvaria and skull base separately, to divide the presumed intramembranous and endochondral bone-forming niches (Fig. 1a and Supplementary Table 1). We captured 336,162 high-quality droplets across 8 shared cellular compartments (Fig. 1c–e, Extended Data Fig. 1a–e and Supplementary Table 2). High concordance was observed between the transcriptome and ATAC peak profile across compartments (Extended Data Fig. 1f). Mesenchymal cells were predominant across all regions, whereas myogenic cells were absent in the calvaria (Fig. 1f). From these, we defined over 100 fine-grained clusters (Supplementary Table 3) and captured a diversity of chondrogenic and osteogenic populations in our data compared with previous published single-cell data (see Methods; Extended Data Fig. 2 and Discussion in Supplementary Information). To resolve bone-lineage cell states and their spatial organization in the nascent synovial joint, we performed high-resolution 155-plex ISS of the whole intact early embryonic forelimbs (6.7 PCW) and hindlimbs (5.7 PCW), and late embryonic (7.3 PCW) knee and shoulder regions (Fig. 1d, Extended Data Fig. 3a–c and Supplementary Table 4). In addition, we conducted sequencing-based spatial transcriptomics (10X Genomics Visium CytAssist) of the developing coronal suture (9 PCW) and frontal bone (Extended Data Fig. 3d), allowing capture of osteolineage maturation across space. We then leveraged these data to systemically curate cell lineages within the mesenchymal compartment in a spatial context. This enabled the discovery of osteogenic cell states in the appendicular regions and skull base (endochondral ossification) and calvarium (intramembranous ossification), reflecting different mechanisms for osteoblastogenesis. Although postnatal mice leverage endochondral ossification during suture closure^14^, it remains unclear whether this is typical in prenatal mice or human sutures. We found that chondrogenic clusters were relatively depleted in the calvarium (Fig. 3a and Extended Data Fig. 4a), consistent with previously reported mechanisms of intramembranous bone formation^15^. We also observed greater cell-abundance discrepancies across droplet and spatial data, particularly at later developmental stages (Supplementary Fig. 1 and Discussion in Supplementary Information).

## Zonation of the embryonic synovial joint

**Fig. 2:**
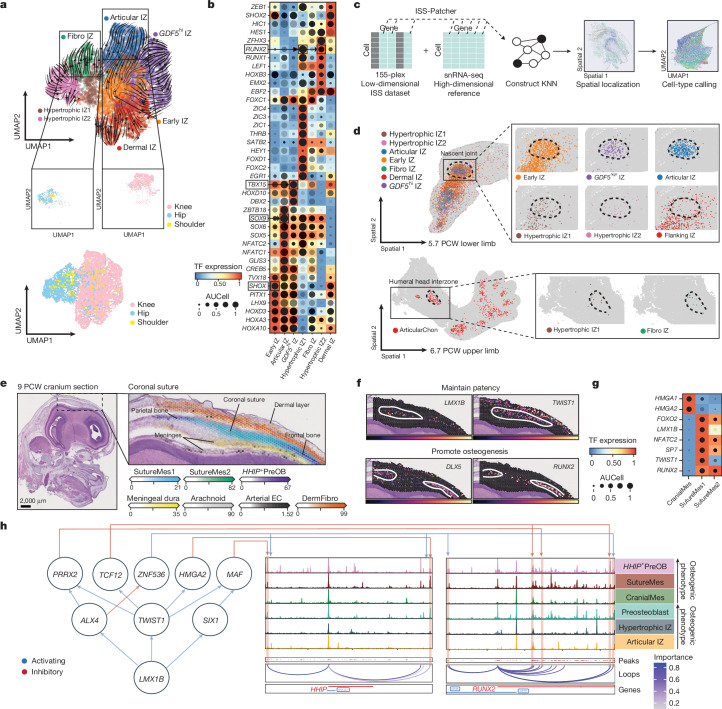
Formation of embryonic joints across space and time. **a**, RNA velocity on UMAP of RNA-subclustered cell states of the broad InterzoneChon cluster, with colours indicating each interzone cluster (top). UMAP coloured according to anatomical region (bottom). **b**, SCENIC+-predicted transcription factor expression (box colour). The dot size shows target gene accessibility (AUCell), and the dot shade (greyscale) shows target gene expression (GEX AUCell). **c**, ISS-Patcher workflow schematic (see Methods). KNN, *k*-nearest neighbour. **d**, Spatial plots of ISS-Patcher-imputed cell clusters. An ISS image of the hindlimb with the imputed interzone clusters overlaid is shown (top left), and individual imputed interzone clusters within the knee interzone are also shown (top right). An ISS image of the forelimb with the imputed articular chondrocyte cluster (bottom left), and individual imputed interzone clusters within the shoulder interzone (bottom right) are also displayed. **e**, A haematoxylin and eosin staining section of a cranium in sagittal view at 9 PCW (left), and a region on the adjacent section profiled using 10X CytAssist Visium data (right). Images are representative of one donor. Cell2location results of the coronal suture, showing enrichment of cluster labels in each voxel, are also shown (right). **f**, Normalized transcription factor expression plotted in Visium voxels of the Visium data in panel **e**. **g**, SCENIC+-predicted transcription factor expression (box colour). The dot size shows target gene accessibility (AUCell), and the dot shade (greyscale) shows target gene expression (GEX AUCell) in suture progenitors. **h**, Coverage plots showing aggregated single-cell ATAC signals around the *HHIP* and *RUNX2* loci for osteoprogenitor cell states with increasing osteogenic phenotype in intramembranous (CranialMes to *HHIP*^*+*^PreOB) and endochondral (Articular IZ to preosteoblast) ossification. Below each coverage plot, loops predicted by SCENIC+ between the transcription start site and enhancers are shown (coloured by importance score). Selected upstream transcription factors predicted by SCENIC+ to bind and regulate via some of the enhancers are also shown (left). The network links inhibitors of osteogenesis (such as *TWIST1* and *LMX1B*) to pro-osteogenic genes (*RUNX2* and *HHIP*) via overall inhibitory connections.

Synovial joint-site determination occurs between 5 and 6 PCW in the limbs, in an initial mesenchymal condensation comprising *GDF5*-expressing interzone cells^13,16^. We applied differential abundance testing on developmental stages and identified InterzoneChon as the earliest joint cluster (Extended Data Fig. 4a–c and Discussion in Supplementary Information). Subclustering of the InterzoneChon (*GDF5*^*+*^*PITX2*^*+*^) population and RNA velocity dynamics analysis (see Methods) allowed inference of their pseudotime trajectory (Fig. 2a and Discussion in Supplementary Information). We applied SCENIC+ to predict gene programs and transcription factor accessibility changes across the seven interzone subclusters (Fig. 2b and Supplementary Table 5). The early interzone population (*PRRX1*) enriched for transcription factors associated with limb mesenchymal development (*TBX18*, *SHOX* and *LHX9*)^5^ and had low *RUNX2* expression and target gene accessibility, but moderate *SOX5*, *SOX6* and *SOX9* expression and target accessibility, suggesting a poised trajectory favouring chondrogenesis over osteogenesis. The articular, fibro and *GDF5*^hi^ interzone clusters highly expressed *GDF5*, and each had distinct gene signatures (see Discussion in Supplementary Information). We hypothesize that the *GDF5*^hi^ interzone cluster, which showed low activity for chondrogenic and osteogenic transcription factors, is undifferentiated and has the potential to sustain influx into the forming joint^16^. We leveraged our newly developed ISS-Patcher function (see Methods) to infer cell labels in the 155-plex-clustered ISS data manifold (Fig. 2c). In the embryonic hindlimb, the early interzone cluster was diffusely distributed across regions of the interzone and cartilage scaffold, and was surrounded by the dermal interzone (Fig. 2d). The articular interzone cluster was predominantly enriched in sites of incipient knee articular cartilage, which colocalized with *SOX9* staining (Extended Data Fig. 4c). By contrast, the fibro interzone cluster^17^, which expressed meniscus-related (*PTN*) and ligament-related (*POSTN* and *SCX*) genes (Extended Data Fig. 4b), was enriched in the shoulder interzone region adjacent to the articular surface of the humerus^18^ (Fig. 2d). The comparative paucity of the fibro interzone cluster enriched in the hindlimb may be due to the earlier formation of the shoulder fibrocartilage than of the joints in the hindlimb^19,20^. From these spatial enrichment patterns, we demonstrated zonation of the presumptive joint, showing early chondrogenic and anti-osteogenic transcription factor enrichment in the interzone centre, and *RUNX2* enrichment in the developing cartilage scaffold (Extended Data Figs. 3d and 4d). Using gene-enrichment scoring, our data suggest that joint cavitation occurs subsequent to zonation and takes place between 7 and 8 PCW (Supplementary Fig. 2 and Discussion in Supplementary Information).

## Emergence of fibroblast lineages

**Extended Data Fig. 5:**
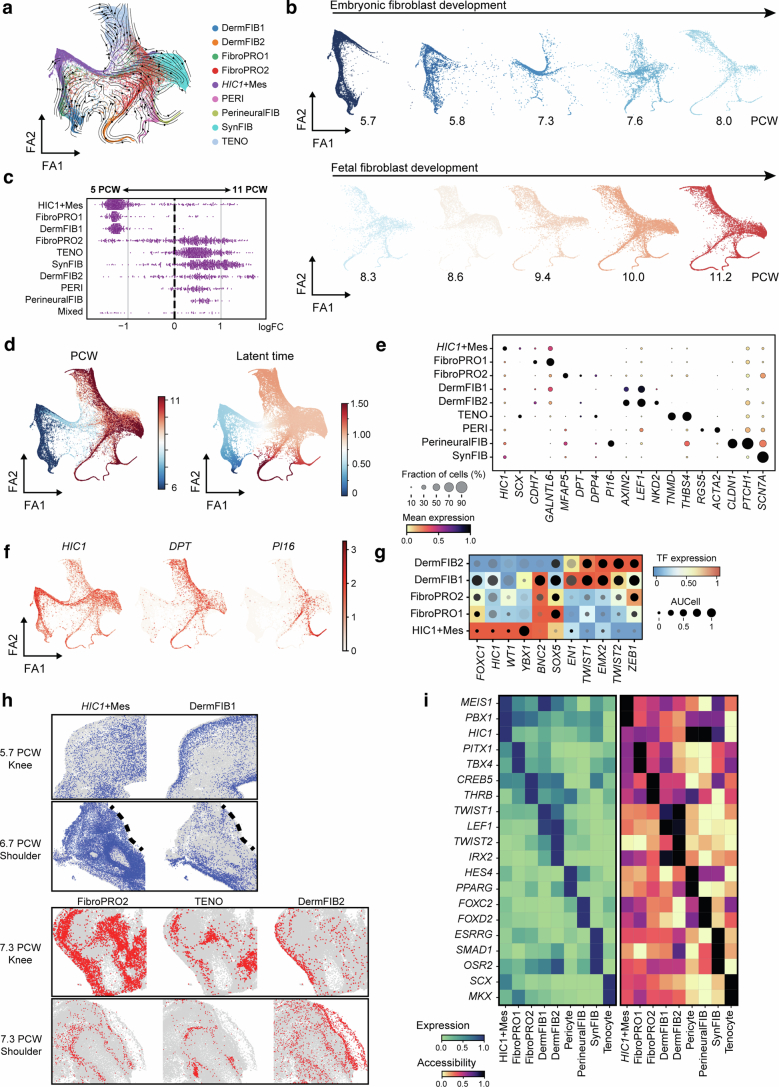
Fibroblast cell states in the appendicular joints. **a**. Force-directed embedding of fibroblast cell states. Arrows show RNA velocity. **b**. Developmental stage (/PCW) displayed over force directed embedding. **c**. MILO cell type abundance swarm plot for fibroblast cell states over developmental time. **d**. Developmental stage (/PCW) and pseudotime (latent-time) on force-directed embedding of fibroblast clusters. **e**. Dotplot showing marker genes expression per cell state. **f**. Visualisation of marker genes on force-directed embedding. **g**. TF activity across select fibroblast clusters. Colour shows normalised expression, dot size shows target gene accessibility (ATAC AUCell) and dot shade (grayscale) shows target gene expression (GEX AUCell). **h**. Spatial location of the imputed fibroblast clusters in the embryonic limb (<7 PCW, top) and foetal limb (>7 PCW, bottom). Dotted line shows sectioning artefact on one side of the 6.7 PCW shoulder sample. **i**. Heatmap showing the gene expression and accessibility (gene score from ArchR) of representative TFs in each cell type.

Fibroblast lineage cell states in the embryonic mouse limb arise from a master *HIC1*^+^ precursor population^21^ that contributes minimally to osteochondral components and gives rise to a postnatal ‘universal’ *PI16*^+^ population, also identified in the adult human across tissues^21,22^. We sought to uncover the taxonomy of the fibroblast lineage in first trimester human joints. We identified a fibroblast progenitor (FibroPRO1), *HIC1*^+^ mesenchyme (*HIC1*^+^Mes) and dermal fibroblasts (DermFIB1 and DermFIB2; see Discussion in Supplementary Information) in the appendicular joints during the embryonic period (less than 8 PCW), surrounding the nascent joint, and with diffuse distribution in the limbs, respectively (Extended Data Fig. 5a–d). At approximately 8 PCW, FibroPRO2 expresses *PI16* and *DPT*, which are markers of pan-tissue adventitia-associated fibroblasts in postnatal health (Extended Data Fig. 5e,f). *BNC2*, a myofibroblast-associated transcription factor^23^, had high activity in FibroPRO1 and FibroPRO2, consistent with its postnatal expression in the vascular adventitia^22^. In addition, *YBX1*, a transcription factor that has been shown to drive proliferation of mouse embryonic fibroblasts, was also enriched. On the basis of developmental time and RNA velocity (see Methods), we predicted *HIC1*^+^Mes as an early fibroblast progenitor during the embryonic phase at less than 8 PCW (Extended Data Fig. 5a,b). Here it was inferred to give rise to tenocytes, synovial fibroblasts, dermal fibroblasts and myofibroblasts (Extended Data Fig. 5a,b). *HIC1*^+^Mes showed high activity of numerous proliferation-associated transcription factors including *WT1*, *SOX5* and *FOXC1*, which are associated with an invasive and activated synovial fibroblast phenotype^24–27^ (Extended Data Fig. 5g), and mapped to the embryonic limb on our RNA-ISS data (Extended Data Fig. 5h). *HIC1*^+^Mes also demonstrated moderate accessibility in tenogenesis transcription factors such as *SCX* and *MKX* (Extended Data Fig. 5i), which is suggestive of tenogenic potential, consistent with fate-mapping in mice^28^ and our trajectory analysis (Extended Data Fig. 5a–d), and is a finding that may warrant functional exploration in future work.

## Formation of the cranial sutures

**Extended Data Fig. 6:**
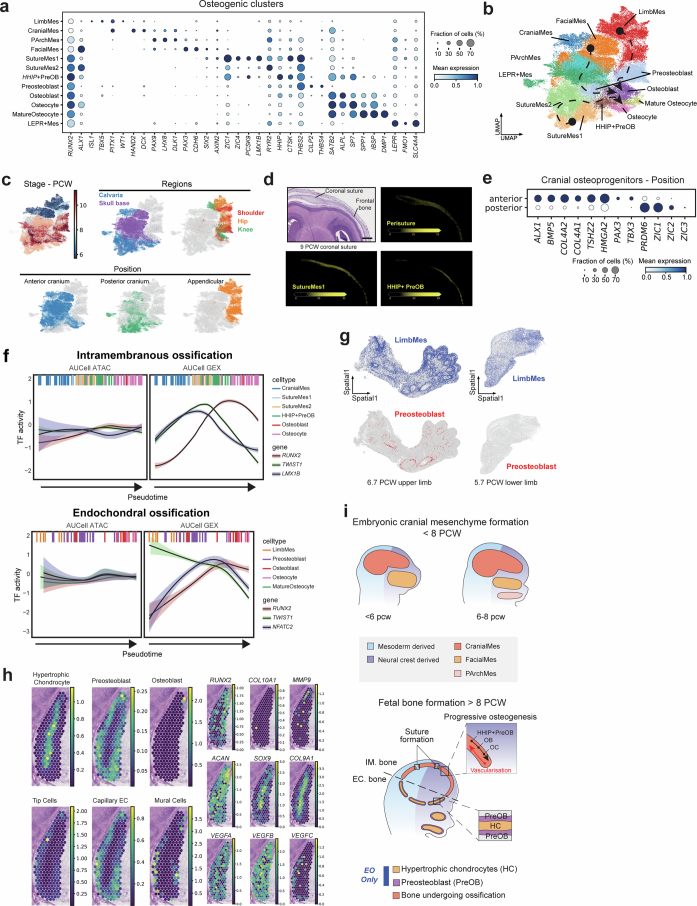
Osteogenesis cell states. **a**. Dotplot of marker genes including those previously described in mouse suture cells across osteogenic clusters. **b**. UMAP embedding of osteogenesis lineage clusters. Arrows show manually annotated paths from progenitor to mature osteogenesis clusters. **c**. Stage (PCW), region and position metadata overlaid on UMAP embedding. **d**. Cell2location cell state enrichment in the coronal suture and frontal bone. Scale bar = 2000 µm. **e**. Dotplot with marker genes in relative anatomical (anterior-posterior) positions in the cranial osteogenic cell states. **f**. Line plot of GEX and ATAC AUC scores across pseudotime for selected TFs and clusters in endochondral and intramembranous osteogenesis lineages. Bands show the 95% CI. Top: higher GEX and ATAC of inhibitory TFs *TWIST1* and *LMX1B* is observed at the beginning of the trajectory (CranialMes), while *RUNX2* shows higher expression and accessibility towards the end of the trajectory. The crossing point at SutureMes1/2 suggests a critical regulatory balance at this cell state. **g**. Imputed osteogenic cell states in the appendicular ISS data **g**. Schematic showing cranial progenitor maturation across embryonic (top) and foetal (bottom) stages. **h**. Cell2location cell cluster enrichment in the sphenoid bone of the skull base (endochondral niche) (Left), gene expression in the same region (right). **i**. Schematic of cranial formation in the embryonic and fetal cranium, showing suture formation and progressive osteogenesis in the fetal cranium, with hypertrophic chondrocytes in the skull base. EO: endochondral osteogenesis, IO: intramembranous osteogenesis.

In the cranium, the incipient suture mesenchyme matures from 7 PCW, forming suture joints^29^. We identified calvaria-abundant early cranial progenitors (CranialMes, FacialMes and PArchMes; Extended Data Figs. 4a and 6a–c and Discussion in Supplementary Information) and *RUNX2*-expressing SutureMes1 and SutureMes2, which also expressed *CTSK*, *SIX2* and *AXIN2*, consistent with mouse cranium progenitors^30,31^, suggesting that they form part of the intramembranous osteogenic lineage (see Discussion in Supplementary Information). Classical markers of fetal mouse cranial sutures (*TWIST1*, *ZIC1* and *ZIC4*) and *THBS2* were enriched in both SutureMes populations^31^. Using Cell2location, we localized these to the developing coronal suture joint (Fig. 2e). Osteoprogenitors (*HHIP*^+^PreOB) emerged at the opposing frontal and parietal bone boundaries of the SutureMes populations (Fig. 2e and Extended Data Fig. 6d). *ALX1*, a transcription factor required for cranium formation in the mouse^32^ and neural crest cell (NCC) migration in human-derived cells, and numerous NCC transcription factors (*PAX3*, *BMP3* and *TSHZ2*) were found to be differentially enriched in cells captured from anterior regions of the cranium (Fig. 1a and Extended Data Fig. 6b,c,e). Although these data suggest that *ALX1*^+^ cells may potentially have a neural crest origin, owing to the transient presence of NCCs^33^, we did not capture bona fide early embryonic *SOX10*^+^ NCCs. Analogous to osteogenic repressors expressed by the articular interzone, SutureMes showed high activity for anti-osteogenesis transcription factors also enriched in mouse cranial sutures (*TWIST1*, *LMX1B* and *NFATC2*)^34^. Simultaneously, osteogenic (*SP7* and *FOXO1*) transcription factor activity was also high, suggesting a GRN primed for bone formation. Comparable with molecular gradients of the embryonic knee (Fig. 2f,g), we observed *LMX1B* and *TWIST1* expression within the suture region, dissipating towards the flanking bone edges concurrent to enrichment of *RUNX2* (Fig. 2f and Extended Data Fig. 4e), suggesting similar mechanisms in sustaining the non-ossifying joint space in both the limb and the cranium. To reveal the enhancer-driven GRN of the loci surrounding the key osteogenic transcription factors *RUNX2* and *HHIP*, we visualized ATAC coverage in combination with SCENIC+-predicted transcription factor–peak and peak–gene links across clusters (Fig. 2h and Supplementary Fig. 3). Both *RUNX2* and *HHIP* were predicted to be inhibited by a shared set of anti-osteogenic transcription factors, including *LMX1B*, *TWIST1* and *ALX4*, via intermediate repressors targeting enhancers around their loci, illustrating the relationships maintaining the balance of osteogenic initiation. *HHIP* was highly accessible in *HHIP*^+^PreOB and was indirectly repressed by *LMX1B* via *TWIST1*. *RUNX2* was most accessible in *HHIP*^+^PreOB (intramembranous ossification) and preosteoblast (endochondral ossification) and was indirectly targeted by the same repressors via *TCF12* and *PRRX2*. Overall, this network illustrates the coherent regulation of bone-adjacent non-ossifying niches by key osteogenic regulators via multiple redundant paths.

## Trajectories of skeletal osteogenesis

**Fig. 3:**
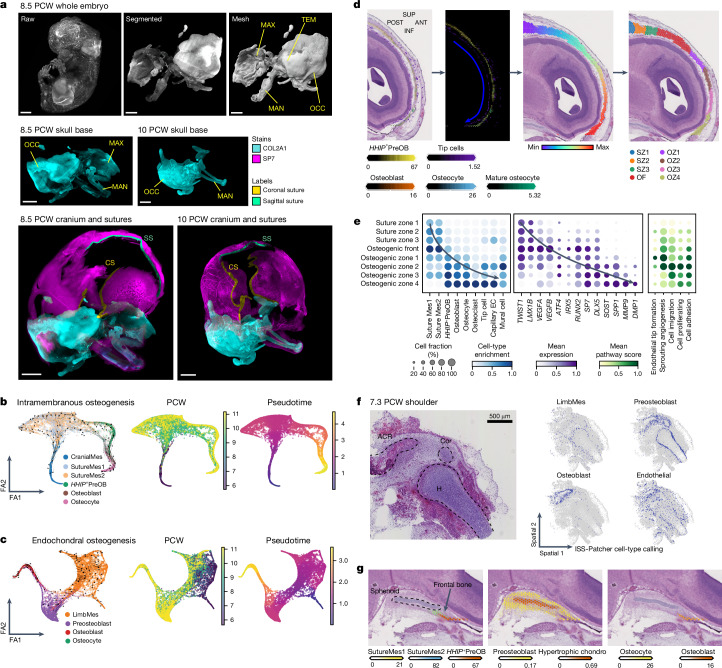
Intramembranous and endochondral osteogenic niches. **a**, Light-sheet fluorescence microscopy cleared images of the embryo and cranium at 8.5 and 10 PCW. A 3D view of rendered images of the 8.5 PCW embryo, the segmented chondrocranium and a mesh image for 3D rendering (top row); COL2A1 staining in the chondrocranium for both samples (middle row); and COL2A1 and osterix (SP7) staining overlaid across the whole cranium, with evident overlap in COL2A1 and SP7 staining in the skull base, but limited COL2A1 expression in the calvarium (bottom row) are shown. Coronal sutures (CSs) and sagittal sutures (SSs) are coloured according to the label legends. Images are representative of two donors. Scale bars, 2,000 μm. MAN, mandible; MAX, maxilla; OCC, occipital; TEM, temporal. **b**, Force-directed (FA) embedding showing the trajectory of intramembranous ossification in the calvaria suture mesenchyme. Embeddings show RNA velocity-predicted pseudotime trajectory arrows, PCW metadata and scFates-computed pseudotime, respectively (left to right). **c**, As in panel **b**, of a force-directed embedding showing the trajectory of endochondral ossification in the limb mesenchyme and preosteoblast. **d**, Spatial binning of a 10X Visium CytAssist image of the frontal bone (sagittal section) using OrganAxis. The axis spans the coronal suture to the frontal bone (posterior–anterior). From left to right: haematoxylin and eosin staining image of the frontal bone, cell-cluster enrichment from Cell2location, axis values (rainbow scale) and manual bins based on histological features. Images are representative of three sections from one donor. ANT, anterior; INF, inferior; OF, osteogenic front; OZ, osteogenic zone; POST, posterior; SUP, superior; SZ, suture zone. **e**, Enrichment within spatial bins defined in panel **d**, for intramembranous cell states (left), selected marker gene expression (middle) and pathway enrichment (right). EC, endothelial cell. **f**, A 7.3 PCW humerus (H; coronal section) and imputed cell clusters showing endochondral ossification as a sequence of mapped cell states. Images are representative of sections from three donors. Cor, coracoid process. **g**, Sagittal section of the sphenoid bone and cell-state enrichment from Cell2location. Images are representative of one donor. Source data

**Extended Data Fig. 7:**
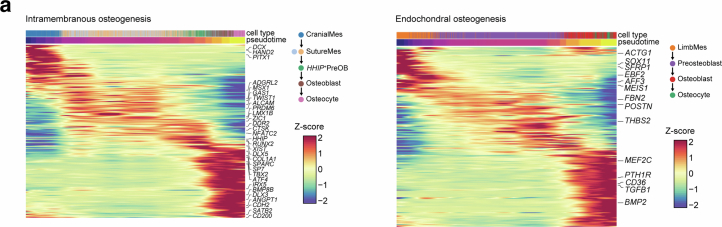
Gene changes across osteogenesis. **a**. Heatmaps of top differentially expressed genes along the Intramembranous and Endochondral trajectory pseudotime (spatial autocorrelation test with monocle3, see Methods).

**Extended Data Fig. 8:**
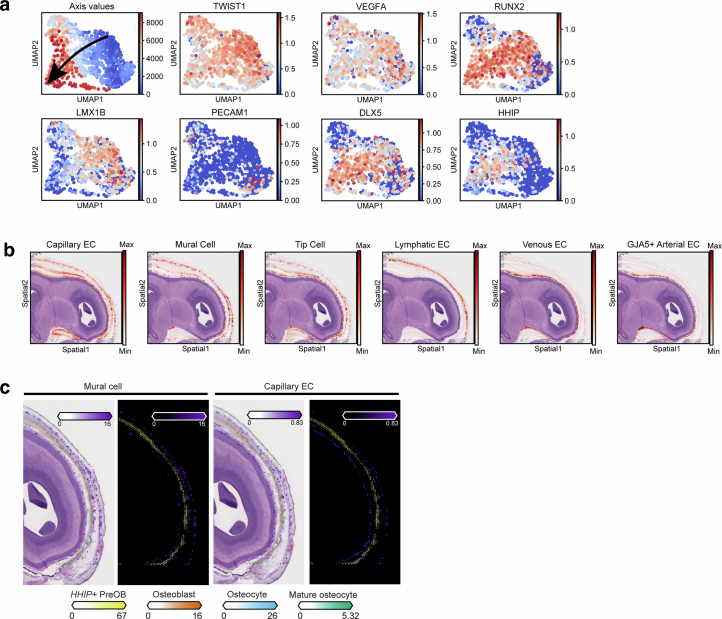
Cellular interactions within the suture niche. **a**. Clustering of Visium voxels with organ-axis values and gene expression. **b**. Cell2location endothelial cell state enrichment in the anterior portion of the cranium **c**. Cell2location enrichment of osteogenic cell states alongside mural (left) and capillary EC (right) on the anterior portion of the frontal bone.

Osteoblastogenesis commenced from approximately 7 to 8 PCW and was apparent in the cranium by 8 PCW (Fig. 3a, Extended Data Fig. 4a and Supplementary Videos 1 and 2). To study this, we inferred two major osteoblastogenic trajectories from distinct osteoprogenitors, which enriched osteogenic transcription factors and downregulated anti-osteogenic transcription factors along pseudotime (Fig. 3b,c, Extended Data Figs. 6f and 7a, Supplementary Tables 6 and 7 and Discussion in Supplementary Information). Endochondral ossification of the limb was predicted to stem from limb mesenchyme (LimbMes), a cluster sampled from, and mapping to, both forelimbs and hindlimbs and is transcriptionally similar to the lateral plate mesoderm (*WT1*) in the fetal human limb bud^5,22^ (Fig. 3c, Extended Data Figs. 6a–c and 7a and Discussion in Supplementary Information). CranialMes and FacialMes were differentially abundant in the anterior portion of the calvarium and skull base, respectively (Extended Data Fig. 4a), and expressed the NCC-derived mesenchymal regulators *PAX3* and *ALX1* (ref. ^35^) (Extended Data Fig. 6a,e). We hypothesize that these clusters constitute previously undescribed human NCC-derived osteogenic populations (see Discussion in Supplementary Information). Suturogenesis was predicted to occur from CranialMes differentiating into SutureMes1 and/or SutureMes2 (Fig. 3b and Extended Data Fig. 7a), forming a predicted trajectory towards *HHIP*^+^PreOB. *HHIP* marks the osteogenic coronal suture mesenchyme in mice^36^, and we demonstrate here that they enrich in a distinct population progeny to *TWIST1*-enriched SutureMes1 and/or SutureMes2 and is distributed in the ossifying cranial bone in human fetal development (Fig. 2f). Following suture formation, progressive waves of oriented differentiation emanate from the cranial sutures towards the developing bone front^31^. We applied OrganAxis (see Methods) to define a continuous maturation axis spanning the coronal suture to regions of the maturing frontal bone. Using zonal bins based on histological features, we evaluated cell-state mapping along the anterior–posterior axis (Fig. 3d). Enrichment of *TWIST1*^+^ SutureMes1 and/or SutureMes2 was observed in the suture zones (1–3; Fig. 3e). Within the osteogenic front, histological features of osteoprogenitors emerged along with *HHIP*^+^PreOB enrichment. Establishment of the osteogenic zones coincided with downregulation of anti-osteogenic (*LMX1B* and *TWIST1*) and upregulation of pro-osteogenic (*RUNX2*, *DLX5* and *SP7*) transcription factors, signifying a spatial molecular switch that zonates territories of the suture (Figs. 2f,g and 3d,e and Extended Data Fig. 6f). Osteoblast markers including *IRX5*, *SOST*, *SPP1*, *MMP9* and *DMP1* peaked towards the distant osteogenic zones^37^, and enrichment of osteogenic transcription factors aligned with axis values away from the coronal suture (Fig. 3e and Extended Data Fig. 8a). In the limb ISS data, we applied ISS-Patcher and identified spatial localization of LimbMes, which is predicted to differentiate to preosteoblasts in the epiphysis and osteoblasts in the incipient bone from 7.3 PCW (Fig. 3f and Extended Data Fig. 6g). In the more mature skull base at 9 PCW, a comparable pattern was detected in the sphenoid where hypertrophic chondrocytes of the cartilage scaffold were surrounded by preosteoblasts (Fig. 3g).

## Angiogenesis in the osteogenic niches

**Extended Data Fig. 9:**
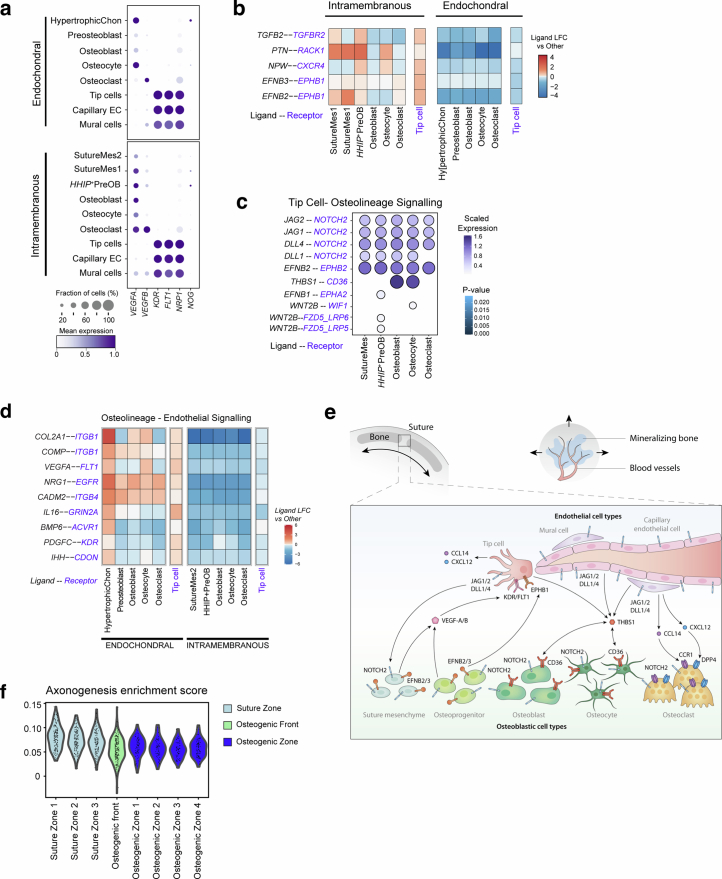
Cellular interactions within the suture niche. **a**. Dotplot of gene expression of receptor-ligands related to angiogenesis in IM and EC clusters. **b**. NicheNet predictions of differentially enriched cell-cell interaction pairs between endothelial tip cells and osteolineage cells in the IM pathway, compared to the EC pathway. **c**. CellphoneDB predictions of Ligand-receptor pairs between endothelial tip cells and cells of the osteogenic lineage. **d**. NicheNet inferred differential cell-cell interactions between osteogenic cell states and Tip cells in the EC and IM niches. **e**. Schematic of endothelial and osteogenic cell state interactions at the boundaries of the suture mesenchyme. **f**. Enrichment of axonogenesis gene set from the Gene-ontology 2023 database across the organ-axis spatial bins.

**Extended Data Fig. 10:**
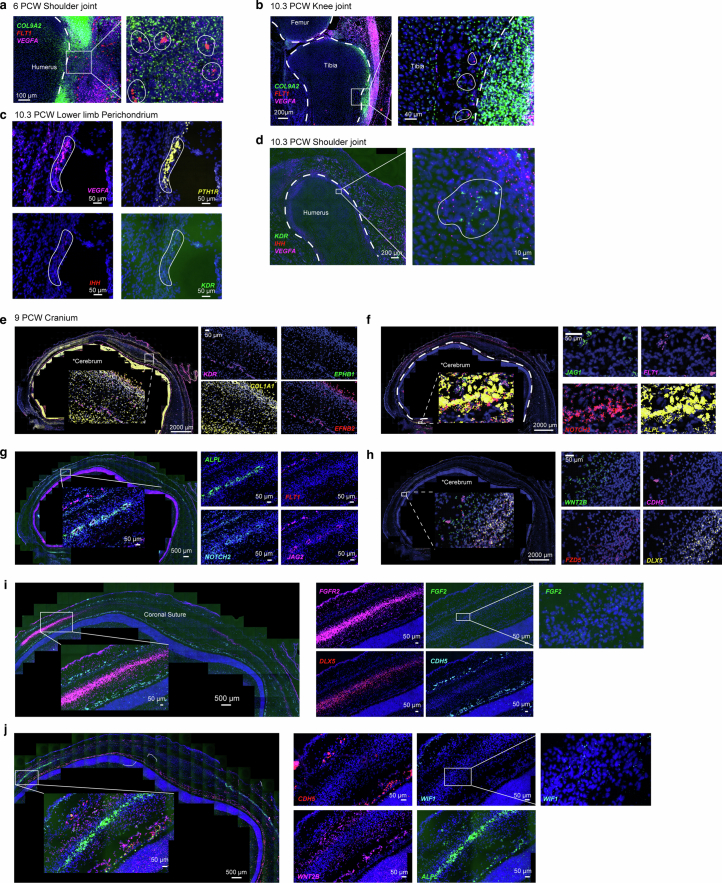
Cell-Cell interactions in the skeletal joints. RNA-ISH of tissue sections from **a**. shoulder joint, **b**. knee joint, **c**. lower limb, **d**. shoulder joint, **e-j**. cranium, showing expression patterns of *COL9A2*, *FLT1*, *VEGFA*, *PTH1R*, *IHH*, *KDR*, *EPHB1*, *EFNB2*, *COL1A1*, *JAG1*, *NOTCH2*, *ALPL*, *JAG2*, *WNT2B*, *CDH5*, *FZD5*, *DLX5*, *FGFR2*, *FGF2*, and *WIF1*. Images are representative of sections from three donors (**a**-**j**).

The basis for endothelia sprouting, which drives osteogenesis within the human intramembranous ossification niche, remains undefined^38^. Within the intramembranous niche, tip cells, mural cells and capillary endothelial cells progressively co-enriched along the osteogenic zones with osteoblast and osteocytes (Fig. 3e, Extended Data Fig. 8a–c and Discussion in Supplementary Information). Accordingly, SutureMes1 and/or SutureMes2 and *HHIP*^+^PreOB highly expressed *VEGFA* and *VEGFB*, which are modulators of vessel sprouting, and localized to the osteogenic front where endothelial cells highly expressed *VEGF* receptor genes (*FLT1*, *KDR* and *NPR1*; Extended Data Fig. 9a). These suggest that SutureMes1 and/or SutureMes2 and *HHIP*^+^PreOB may promote vascular invasion in intramembranous niches, akin to chondrocytes in endochondral ossification niches^39,40^. Through RNA in situ hybridization (RNA-ISH), we observed *VEGFA* and *KDR*–*FLT1* coexpression in the appendicular cartilage (Extended Data Fig. 10a–d). Our Visium data also demonstrated enrichment of *VEGFA* expression in the hypertrophic cartilage of the skull base, colocalizing with capillary endothelial cells mapped by Cell2location (Extended Data Fig. 6h). We observed a spatial gradient of angiogenesis along the cranial frontal bone maturation axis by scoring for enrichment in sprouting angiogenic pathways (Fig. 3e), suggesting an association between angiogenesis and osteogenesis. Using NicheNet, we predicted osteolineage–tip cell interactions specific to the intramembranous niche and identified colocalization of these pairs using RNA-ISH (Extended Data Figs. 9b and 10e–j and Discussion in Supplementary Information). We used CellphoneDB to predict signalling interactions from ligands in the endothelial cell-to-osteogenic cell states^41^. Tip cells were predicted to signal via *NOTCH*, including JAG1/JAG2–NOTCH2 (Extended Data Fig. 9c and Discussion in Supplementary Information), which have been reported to promote differentiation of postnatal perivascular osteoprogenitors^42^. Mural and capillary endothelial cells expressed the ligand genes *FGF2* and *RSPO3*, which have been previously described to facilitate osteoblast differentiation via FGF2–FGFR2 (ref. ^43^) and RSPO3–LGR5 (Extended Data Fig. 9d and Discussion in Supplementary Information), and THBS1–CD36 (ref. ^44^). Endothelial cells also expressed *CCL14* and *CXCL12* — encoding the ligands for CCR1 (ref. ^45^) and DPP4 (ref. ^46^), respectively — which support in vitro osteoclast recruitment and differentiation. These spatially defined interactions suggest tip cell recruitment by VEGFA and EPHB2 from osteolineage cells in the bone front. We theorize that the vascularizing endothelial cells then promote osteoblastic differentiation, osteocyte mineralization and osteoclast recruitment in the maturing bone (Extended Data Figs. 6i and 9e). Other lineages, such as neurons, that may modulate osteogenesis were not captured in our droplet data, and future studies of the innervating neurons may shed light on the potential neuron–osteolineage interactions at work (Extended Data Fig. 9f and Discussion in Supplementary Information).

## Developmental chondrocyte heterogeneity

**Fig. 4:**
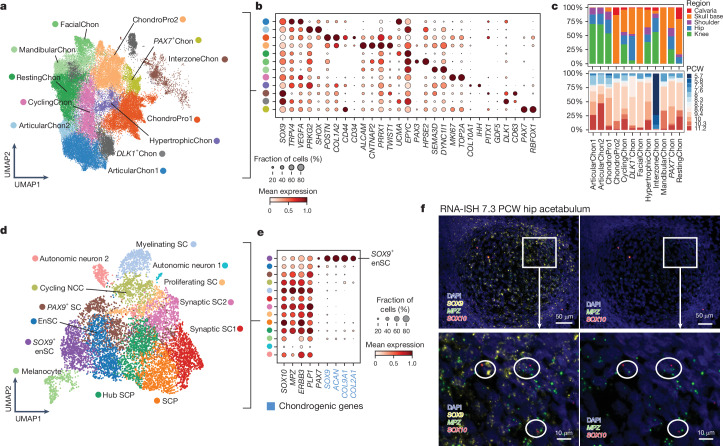
Chondrogenesis across anatomical regions. **a**, UMAP embedding of chondrocytes coloured by subtypes. **b**, Dot plot showing the mean expression (dot colour) and fraction of expressing cells (dot size) of selected marker genes. **c**, Proportion of chondrogenic cell clusters across anatomical regions and developmental age (PCW). **d**, UMAP embedding of Schwann lineage cell states. SC, Schwann cell; SCP, Schwann cell precursor. **e**, Dot plot showing mean expression (dot colour) and fraction of expressing cells (dot size) of chondrogenesis-associated genes across Schwann cell states. The gene names in blue indicate markers of *SOX9*^*+*^ enSC. **f**, Three-plex RNA-ISH (RNAscope) of marker genes in the 7.3 PCW hip acetabulum demonstrating enrichment and colocalization of markers of *SOX9*^*+*^ enSC (*MPZ*^*+*^*SOX9*^*+*^) and Schwann cells (*MPZ*^*+*^*SOX10*^*+*^). Images are representative of sections from one donor.

**Extended Data Fig. 11:**
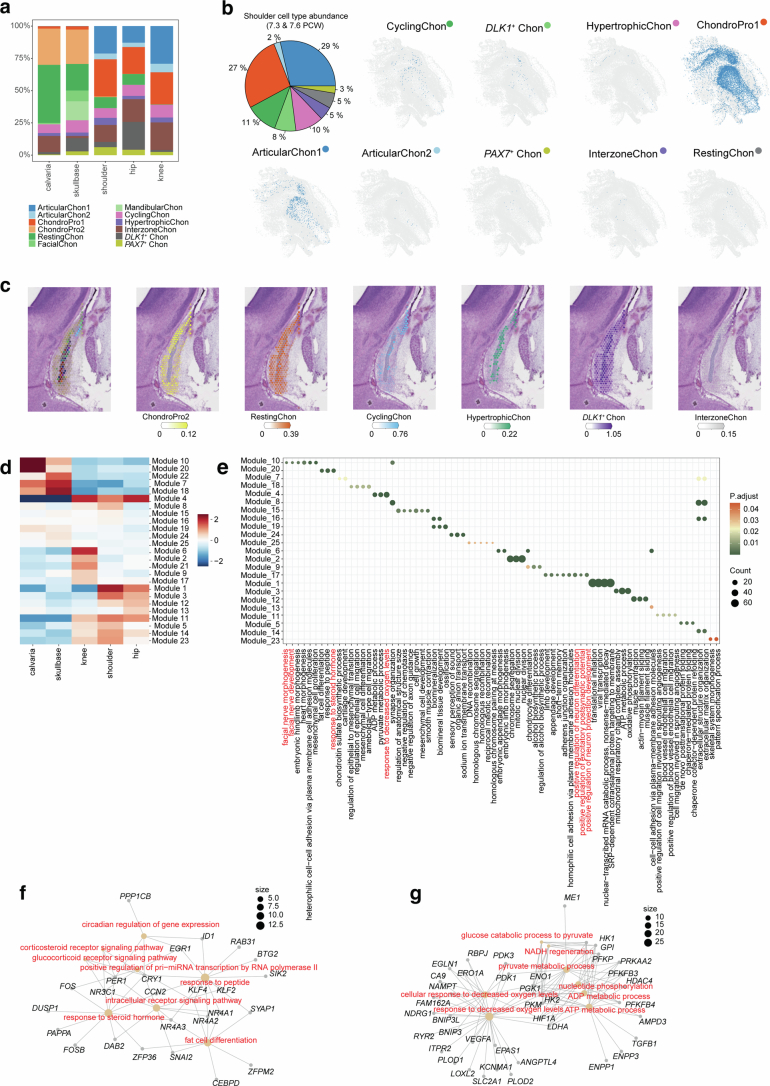
Spatial locations and gene signatures of chondrocytes. **a**. Barplot showing the distribution of cell cluster abundance of chondrocytes per anatomical region and per PCW. **b**. Pie chart (left) showing the percentage of cell clusters in the shoulder at 7.3 and 7.6 PCW. Spatial plots (right) showing predicted cell clusters from ISS-Patcher cell cluster imputation. Colours represent the corresponding cell clusters. **c**. Histological view and annotations of chondrocytes in the sphenoid sections from 10x Cytassist Visium. **d**. Heatmap showing kernel values of module genes per anatomical region. **e**. Top 3 enriched biological process GO terms for each module. The GO terms were selected based on gene number, and terms of the same gene number remain. Colour represents the adjusted p-values, while the dot size represents the gene number of each GO term (see Supplementary Table 11). The statistical test was performed using a two-sided hypothesis. P values were adjusted with the Benjamini–Hochberg method. **f**. Network visualisation of enriched genes of calvaria and skull base-specific GO terms. **g**. Network visualisation of enriched genes of appendicular-specific GO terms.

**Extended Data Fig. 12:**
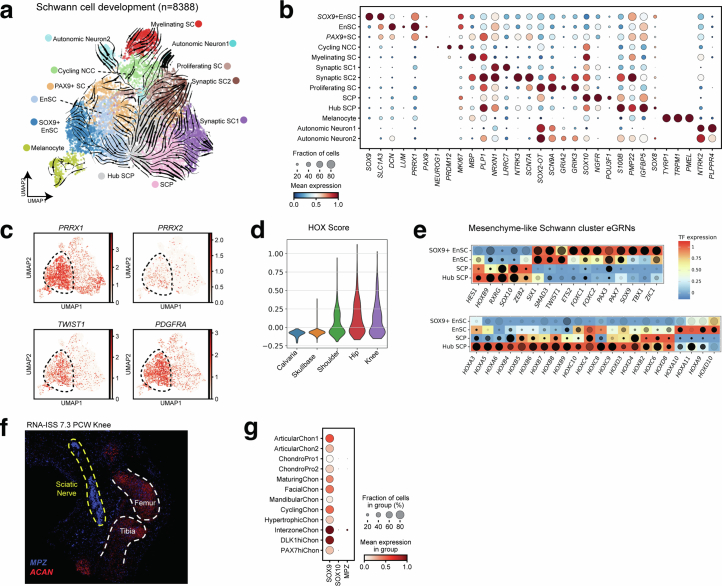
Schwann cell lineage development. **a**. RNA-velocity streamlines displayed on embedding of Schwann lineage cell states, with numerous cell states emerging from Hub SCP (Schwann cell precursor). **b**. Dotplot showing marker gene expression across Schwann cell states. **c**. Expression of mesenchyme associated genes in the Schwann UMAP. **d**. Enrichment of *HOX* genes per anatomical region of the Schwann compartment. **e**. TF expression across the putative chondrogenic lineage of the Schwann clusters. Colour shows normalised expression, dot size shows target gene accessibility (AUCell) and dot colour (grayscale) shows target gene expression (GEX AUCell). **f**. Marker genes of ISS RNA probes in the ISS image of the knee joint demonstrating nerve-associate enrichment of Schwann markers, and the presence of *MPZ* staining in the developing bone. Images are representative of sections from three donors. **g**. Dotplot showing gene expression of Schwann cell markers across chondrocyte clusters.

Various types of cartilage, including hyaline, fibrous and elastic cartilage, are formed during development. Our data identified diverse chondrocyte clusters that exhibited strong region-specific abundance and gene modules^47^ (Fig. 4a–c, Extended Data Fig. 11a–g, Supplementary Tables 3 and 8 and Discussion in Supplementary Information). Along with previously described populations, we identified new clusters: two region-specific chondrocyte progenitors (ChondroPro1 and ChondroPro2; Discussion in Supplementary Information) and *DLK1*^+^ chondrocytes (*DLK1*^+^Chon: *DLK1* and *CD63*). ChondroPro1 and ChondroPro2 were enriched in appendicular joints and the skull base, respectively, and expressed fibroblast differentiation markers (*POSTN*, *COL1A1*, *PRRX1* and *TWIST1*), consistent with findings in early chondrocyte progenitors in mice^17^. *DLK1*^+^Chon was enriched in ribosomal genes and *CD63*, which has been identified in the pre-hypertrophic layer in the limb, whereas *DLK1* itself is a marker for embryonic lineage progression from proliferative to pre-hypertrophic phenotypes^48^. Spatially, CyclingChon, *DLK1*^+^Chon and HyperChon were organized sequentially within the nascent bone, spanning from the epiphysis towards the diaphysis, the incipient primary ossification centre (Extended Data Fig. 8b), suggesting a transitional state within *DLK1*^+^Chon, between proliferative and pre-hypertrophic chondrocyte phenotypes. We also characterized previously undescribed craniofacial populations including facial (FacialChon) and mandibular chondrocytes (MandibularChon), which highly expressed *PAX3* and *SEMA3D*, respectively, implying potential origins from the neural crest^49–51^. Lineage tracing in zebrafish and mice^52^ has shown that Schwann cells can differentiate into chondrocytes during embryogenesis. Our data captured a diverse Schwann compartment, and revealed *SOX9*^+^ endoneurial Schwann cells (*SOX9*^+^ enSC) characterized by the expression of chondrocyte (*SOX9*, *COL9A1*, *ACAN* and *COL2A1*) and classical Schwann cell markers (*MPZ* and *SOX10*; Fig. 4d,e and Extended Data Fig. 12a–c). *SOX9*^+^ enSC represented an end point in the trajectory analysis predictions, stemming from Schwann cell precursors, and expressed mesenchymal (*PRRX1*, *PRRX2*, *PDGFRA* and *TWIST2*) and HOX (*HOXA9*, *HOXA10*, *HOXA11* and *HOXD10*) signatures (Extended Data Figs. 12d,e). Through RNA-ISH and RNA-ISS, we observed widespread colocalization of Schwann marker *MPZ* and *SOX9* transcripts within the hip cartilage of the developing hindlimb and a smaller number of cells that coexpress *SOX10* (Fig. 4e,f, Extended Data Fig. 12f and Supplementary Table 9). Accordingly, our chondrocyte clusters do not express *SOX10* or *MPZ* (Extended Data Fig. 12g), indicating that *SOX9*^*+*^ Schwann populations are present within the cartilage. A recent study has also identified Schwann cells within the cartilage of the developing hindlimb digits^53^. Although lineage-tracing experiments are needed to investigate further, we theorize that akin to mice, Schwann lineage cells may be a non-canonical source of chondrocytes in human development. Our droplet data also captured a *PAX7*^*+*^ chondrocyte cluster, which co-expressed markers and gene modules of chondrocytes and muscle cells, and persisted following computational quality control against doublets and ambient RNA (Supplementary Figs. 4–8 and Methods and Discussion in Supplementary Information). Although RNA-ISH demonstrated potential localization of *PAX7* transcripts in chondrocytes of the limb, the signal intensity was relatively modest. Future work, involving post-FACS transcriptional analysis, is needed to investigate the validity of this population.

## Developmental links to complex traits

**Fig. 5:**
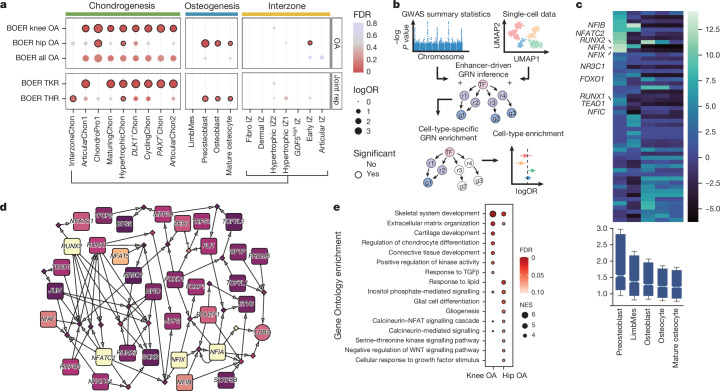
Links to complex diseases of the skeleton. **a**, Enrichment of GWAS signals (fGWAS) for osteoarthritis (OA) and its surrogate phenotypes (replacement (rep) of knee and hip joints), showing how patterns of cell-type enrichment differ between hip-associated and knee-associated single-nucleotide polymorphisms. Enrichment was tested for each cluster-specific logistic regression (Wald test β1 ≠ 0, d.f. = 1, Benjamini–Hochberg (BH) correction, false discovery rate (FDR) < 0.1). BOER, Boer et al.^62^; logOR, log odds ratio; THR, total hip replacement; TKR, total knee replacement. **b**, Schematic representation of the SNP2Cell method; scores were derived from GWAS summary statistics, and cell cluster marker scores are mapped and integrated across a GRN, highlighting enriched modules that are predicted to have a cluster-specific role in disease. **c**, Enrichment scores for hip osteoarthritis of the top ten genes per cluster across the osteogenic cluster (heatmap; top), and a boxplot showing the median enrichment scores, with lower and upper quartiles and whiskers at a maximum of 0.1 the interquartile range across *n* = 1,967 genes and peaks with a *z*-score of more than 2 (bottom). Notches (95% confidence interval around the median) are approximate guides of significant differences between clusters based on their overlap. **d**, Preosteoblast-specific and hip osteoarthritis-specific subnetwork of the most enriched genes and peaks. The brighter colours correspond to greater enrichment scores, relative to scores obtained from random permutations. **e**, Gene set enrichment analysis for Gene Ontology biological process terms across ArticularChon knee osteoarthritis and preosteoblast hip osteoarthritis enrichment scores. NES, normalized enrichment score. Source data

**Extended Data Fig. 13:**
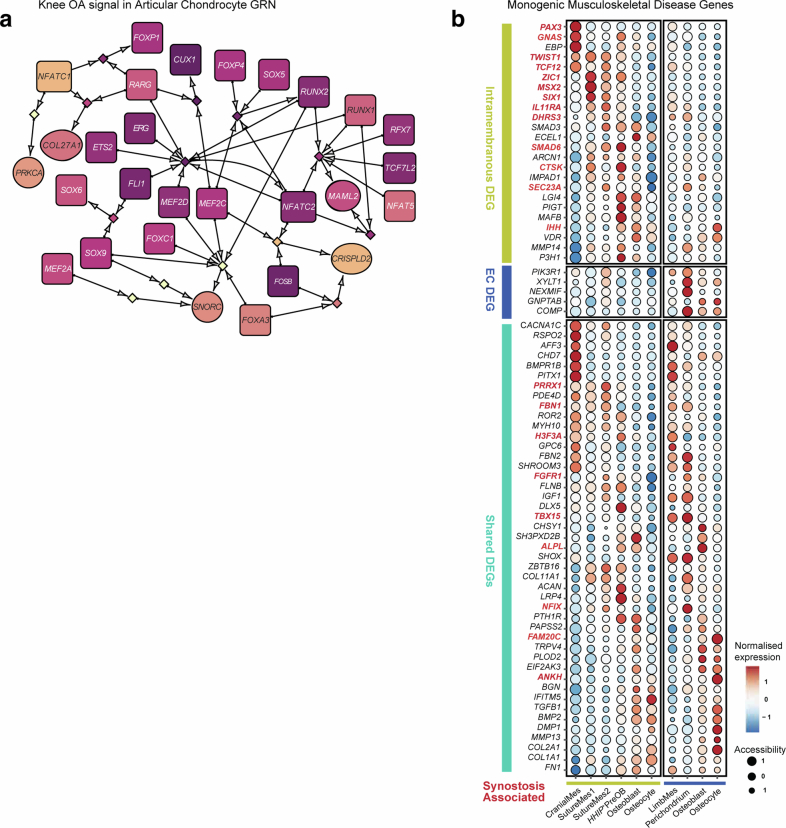
Musculoskeletal disease gene regulation. **a**. eGRN extracted from SNP2Cell (see Methods) showing TFs, target genes and enhancers enriched for knee OA in articular chondrocytes. Brighter colours correspond to a stronger enrichment. **b**. Genes with known mutations in musculoskeletal monogenic conditions that are differentially expressed along the intramembranous, endochondral, or both ossification trajectories. The expression of the genes and accessibility of linked enhancers are shown. Marked in red: genes with a known role in craniosynostosis, the premature fusion of bone plates in the skull.

Numerous conditions of the ageing skeleton have been linked to disrupted joint and bone changes during the embryonic stages of life. Of note, enhancer-associated variants associated with adult osteoarthritis appear to act on anatomical region-specific regulatory networks to influence synovial joint morphology during development^54^. Using functional genome-wide association study (fGWAS; see Methods)^55^, we found knee osteoarthritis signals distinctly enriched in chondrogenic states, except for InterzoneChon (Fig. 5a and Supplementary Table 10). By contrast, hip osteoarthritis enrichment was observed in only two chondrocyte populations (ChondroPro1 and HypertrophicChon), but was enriched in preosteoblasts, osteoblasts and osteocytes. Previous genetic studies have theorized that early bone development affects subsequent risk of hip osteoarthritis, potentially through modulating hip shape and consequent mechanical forces^56^, whereas the knee-specific findings point towards alterations to chondrogenesis. Deriving insights from clusters implicated in fGWAS, we used SNP2Cell to identify cluster-specific sub-GRNs enriching for osteoarthritis signals (see Methods; Fig. 5b and Discussion in  Supplementary Information). Preosteoblasts expectedly displayed the largest average enrichment across the osteogenic pseudotime trajectory for hip osteoarthritis (Fig. 5c), consistent with fGWAS results. We also identified subnetworks for hip osteoarthritis and knee osteoarthritis, prioritizing preosteoblasts (Fig. 5d) and articular chondrocytes (Extended Data Fig. 13a), respectively, and revealed similar regulatory pathways that balance chondrogenic and osteogenic functions. In the articular chondrocyte–knee osteoarthritis network, several non-transcription factor genes with roles in cartilage makeup and chondrocyte differentiation (*COL27A1*, *PRKCA*, *SNORC* and *CRISPLD2*) were predicted to be regulated by *NFATC1* and *FOXA3* (Discussion in Supplementary Information). For hip preosteoblast in hip osteoarthritis, the osteogenic regulator *RUNX2* showed significant enrichment, along with multiple NFAT genes (*NFATC1*, *NFATC2*, *NFATC4* and *NFAT5*), which in conjunction with additional transcription factors (*ZEB1*, *MAF* and *TEAD1*), implicated calcineurin and WNT signalling pathways, which are known to have a role in hip shape formation and osteoarthritis (Discussion in Supplementary Information). Pathway analysis also showed that cellular responses to lipids were enriched in hip preosteoblasts (Fig. 5e and Discussion in Supplementary Information). Overall, through application of fGWAS and our new tool SNP2Cell, we identified differential enrichment of knee and hip osteoarthritis GWAS signals in developmental chondrogenic and osteogenic single-cell profiles, respectively.

## Deciphering monogenic craniosynostosis

**Fig. 6:**
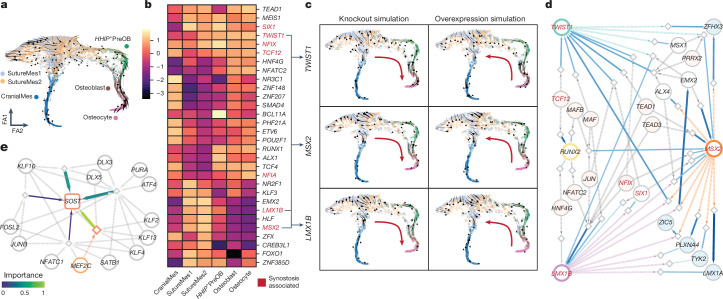
Monogenic conditions affecting bone development. **a**, Force-directed embedding of the intramembranous ossification trajectory. The arrows show directional prediction of differentiation with cytoTRACE, assuming that cells at an earlier time of the trajectory express more genes on average. Consistent with RNA velocity, differentiation from CranialMes to osteocytes is predicted. The arrows on the left side of the SutureMes cluster pointing away from the main trajectory may reflect suture maintenance. **b**, Heatmap of in silico transcription factor-knockout perturbation scores per cell cluster showing the top five transcription factors with the highest scores per cluster. A higher score indicates that transcriptomic changes induced by the perturbation are promoting osteogenesis. The top transcription factors include several genes with a known role in craniosynostosis (marked in red). **c**, Transcription factor perturbation vectors showing the direction of induced transcriptomic changes on the force-directed embedding for three selected genes encoding transcription factors: *TWIST1*, *MSX2* and *LMX1B*. Knockout simulation of all three genes promotes osteogenesis, whereas overexpression inhibits it, suggesting that the transcription factors regulate suture maintenance. **d**, Enhancer-driven GRN showing inter-regulation between *TWIST1*, *MSX2* and *LMX1B*, inferred using SCENIC+. The circles and diamonds represent genes and regions with transcription factor-binding sites, respectively. Region–gene links are coloured and scaled according to peak2gene importance, whereas transcription factor–region links are coloured by transcription factor. The blue circles represent regulated targets, the cream circles indicate regulators and the white circles represent intermediate genes. Transcription factors with a known role in craniosynostosis are marked in red text. The densely connected network contains several of such genes and suggests a potentially similar function for others. **e**, Enhancer-driven GRN showing predicted regulation of *SOST*. A region (orange diamond) containing the enhancer known to be mutated in Van Buchem disease and affecting the regulation of *SOST* is highlighted in orange. Region–gene links are coloured and scaled according to peak2gene importance. Source data

**Extended Data Fig. 14:**
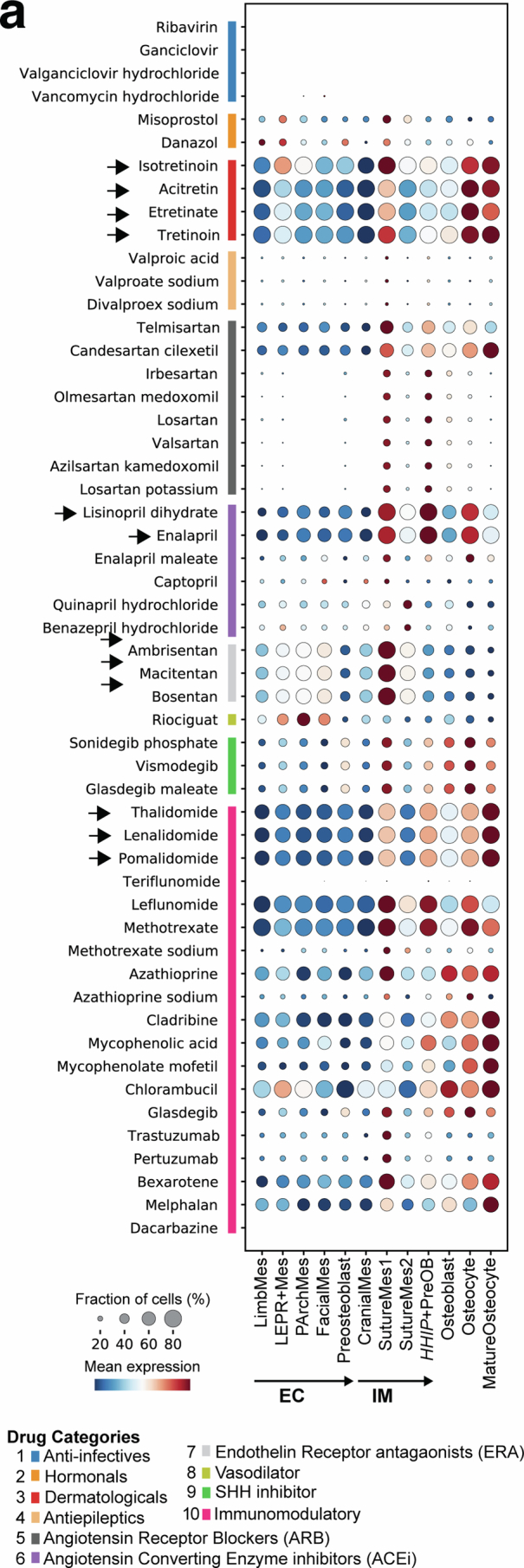
Drug targets in osteogenesis cell states. **a**. Dotplot with drug2cell (chEMBL) enrichment of teratogenic drug target gene expression across cell states of the osteogenic trajectory. Selected highly enriched drugs target gene expression are marked with arrows (y-axis) and cell states unique to either endochondral (EC) or intramembranous (IM) ossification are marked on the x-axis. Patterns of different drugs predicted to affect target genes at different parts of the trajectory are visible.

Craniosynostosis is a congenital condition that involves disturbances in cranial ossification and suture formation during fetal and postnatal development, leading to premature cranial suture fusion and depletion of osteoprogenitor pools (Discussion in Supplementary Information), resulting in global developmental consequences. To predict normal developmental cell states that enrich genes affected in craniosynostosis, we cross-referenced pseudotime-associated differentially expressed genes enriched in the intramembranous and endochondral ossification pathways against a candidate database of over 2,700 genes known to cause congenital conditions in humans^5^ (Extended Data Fig. 13b, Supplementary Tables 6 and 11 and Discussion in Supplementary Information). The majority of enriched craniosynostosis (*n* = 13 of 22) genes were observed in progenitor populations of the intramembranous pseudotime trajectory (SutureMes1, SutureMes2 and *HHIP*^*+*^PreOB). Most of these enriched genes were also highly accessible, apart from *IHH*, suggesting that the embryonic period may be affected in craniosynostosis. To simulate effects of candidate craniosynostosis-associated transcription factor perturbation during normal osteogenesis, we applied CellOracle to predict velocity shifts within the intramembranous trajectory in silico for 485 detected transcription factors. Knockout simulations for *TWIST1*, *MSX2* and *LMX1B* were predicted to lead to high-velocity shifts in SutureMes2 (Fig. 6a–c). *TWIST1* and *LMX1B* also showed spatial enrichment in the coronal suture (Fig. 2f). The direction of inferred velocity changes was also consistent with knockout leading to osteogenesis (Discussion in Supplementary Information). Overall, these predictions may help to inform potential transcriptional effects associated with pathogenic features of transcription factor-mediated craniosynostosis. Future functional studies will be required to reveal mechanistic effects for each gene. We next reconstructed interaction networks across the prioritized transcription factors (*TWIST1*, *MSX2* and *LMX1B*) to resolve their co-regulatory relationship (Fig. 6d). The predictions revealed inter-regulation of shared coding and non-coding targets across these transcription factors. Of the connected nodes, numerous transcription factors (*SIX1*, *TCF12*, *NFIX* and *ALX4*) are known to be associated with craniosynostosis through loss-of-function mutations, suggesting a tightly regulated network conferring suture patency in this region. Of note, *TCF12* has been reported to govern coronal suture development through heterodimer formation with *TWIST1* (Discussion in Supplementary Information), and severe phenotypes are observed in mice with doubly deleterious mutations^57^. We also explored enhancer-mediated regulation of normal development centred around *SOST* to evaluate the role of the ECR5 enhancer, which when mutated leads to Van Buchem disease, a cause of sclerosing dysplasia of bone (Fig. 6e). These predictions of normal development may inform cellular models of transcription factor and enhancer-driven monogenic conditions of the bone lineage. To explore potential cell-extrinsic influences on fetal bone development, we applied Drug2Cell^58^ to score enrichment of genes for teratogenic drug targets within our osteogenic clusters (see Methods; Supplementary Table 12). This revealed overall greater target enrichment of known teratogenic drug targets within intramembranous progenitors and downstream osteoblast or osteocyte cell states, for example, SutureMes1 and *HHIP*^*+*^PreOB, than endochondral progenitors (Extended Data Fig. 14a and Discussion in Supplementary Information). Our analyses allow identification of the bone-lineage clusters that express genes of targets for teratogenic drugs during normal development, and may help to inform the design of future functional studies.

## Discussion

We present a multi-omic cell atlas that captures the spatially resolved cellular taxonomy of human synovial and suture joint formation across the first trimester. In mice, *GDF5*^*+*^ cells are proposed to give rise to all cellular components within the joint, including ligaments and tendons. Here we infer that fibroblasts and tenocytes of the synovial joints arise from embryonic *HIC1*^*+*^ fibroblasts, a population previously reported in the mouse embryonic limb^21^, and a later (*PI16*^*+*^) fibroblast progenitor, which is transcriptionally comparable with human postnatal universal fibroblasts^22^. Together, these shed new light on the lineage origins of the developing human embryonic synovial joint in the first trimester. We identified numerous previously undescribed human cranial embryonic osteolineage cell states including CranialMes (*HAND2*)^59,60^, FacialMes (*PAX3*)^35^ and PArchMes (*LHX8*)^61^, which express comparable markers reported in mice. We defined two spatially resolved *TWIST1*^*+*^ SutureMes populations, and *HHIP*-expressing preosteoblast, which mirror populations of the fetal mouse suture^22,36^ and infer their developmental trajectory in spatial niches. Using OrganAxis, we illustrated the shift in the osteogenic populations across the cranium, uncovering the association between endothelial recruitment and intramembranous ossification. We have shown that the endochondral niche in the limb cartilage scaffold derives from an undifferentiated progenitor expressing *ISL1* or *TBX5*, which marks an equivalent population in the human limb bud^5^, and forms preosteoblasts and osteoblasts. These inferred trajectories form a valuable reference for studies of human bone development in the first trimester (Discussion in Supplementary Information). Through our droplet and spatial data, which capture Schwann cell development in human embryonic bone and joint, we inferred that Schwann cells may confer chondrogenic potential, mirroring previously described mouse development^52^. In future, focused functional investigations that involve isolation of these human cell lineages will further inform their developmental biology. Leveraging our dataset, we uncovered developmental links to both monogenic and complex musculoskeletal diseases. We observed marked differences in the enrichment of hip and knee osteoarthritis GWAS signals across developmental clusters, implicating osteogenesis in hip osteoarthritis and chondrogenesis in knee osteoarthritis. Lastly, by systematically simulating in silico knockout of transcription factors known to be associated with craniosynostosis, we identified a network of regulators inhibiting osteogenesis in the sutures, which provides mechanistic insights of how previously reported monogenic loss-of-function mutations may act. This approach, applied to the osteogenic trajectories, has potential value in aiding exploration of gene effects across other diseases involving embryonic osteogenesis. Our cross-region multimodal human developmental skeletal atlas is a fundamental resource for the understanding of human cartilage and bone development in the first trimester. It also has the potential to inform in vitro endeavours to differentiate osteoblast and other mesenchymal cell states.

## Methods

### Sample acquisition and ethics

Developing human limb and cranium tissue samples were obtained from elective terminations under REC 96/085 with written and informed consent obtained from all sample donors (East of England, with full approval from the Cambridge Central Research Ethics Committee). In brief, samples were kept suspended in PBS and at −4 °C on ice during dissection. Shoulder, hip and knee joints were dissected en-bloc from the limbs. For the shoulder joint, a proximal incision was made at the distal third of the clavicle, and a distal incision was created at the neck of the humerus. For embryonic shoulder samples where distinctive bone features had not formed, approximations were made to capture the entirety of the glenohumeral and acromioclavicular joints. For the cranium samples (less than 8 PCW), two regions were dissected for each of the calvaria and skull base, separated at the posterior border of the frontal bone in both cases. For older cranial samples (more than 8 PCW), tissues were dissected to separate the frontal, parietal, sphenoid, ethmoid, occipital and temporal bones where feasible. Samples were initially embedded in optimal cutting temperature medium and frozen at −80 °C on an isopentane-dry ice slurry. Cryosections were then cut at a thickness of 10 μm using a Leica CM1950 cryostat and placed onto SuperFrost Plus slides (VWR) for ISS or Visium CytAssist, or used directly for single-nucleus processing. For samples used in whole-mount immunostaining, samples were obtained from terminations of pregnancy with written and informed consent obtained from all sample donors. Samples were provided by INSERM’s HuDeCA Biobank and utilized in compliance with French regulations. Full authorization to use these tissues was granted by the French agency for biomedical research (Agence de la Biomédecine, Saint-Denis La Plaine, France; PFS19-012) and the INSERM Ethics Committee (IRB00003888).

### ISS and high-resolution imaging

ISS was performed using the 10X Genomics CARTANA HS Library Preparation Kit (1110-02, following protocol D025) and the In Situ Sequencing Kit (3110-02, following protocol D100), which comprise a commercialized version of HybRISS^63^. Probe panel design was based on fold-change thresholds in cell states of the limbs (Supplementary Table 3). In brief, cryosections of developing limbs were fixed in 3.7% formaldehyde (252549, Merck) in PBS for 30 min and washed twice in PBS for 1 min each before permeabilization. Sections were briefly digested with 0.5 mg ml^−1^ pepsin (P7012, Merck) in 0.1 M HCl (10325710, Fisher) at 37 °C for 15 s (5 PCW) or 30 s (6 PCW and older), then washed twice again in PBS, all at room temperature. Following dehydration in 70% and 100% ethanol for 2 min each, a 9-mm diameter (50 μl volume) SecureSeal hybridization chamber (GBL621505-20EA, Grace Bio-Labs) was adhered to each slide and used to hold subsequent reaction mixtures. Following rehydration in buffer WB3, probe hybridization in buffer RM1 was conducted for 16 h at 37 °C. The 158-plex probe panel included 5 padlock probes per gene, the sequences of which are proprietary (10X Genomics CARTANA). The section was washed with PBS-T (PBS with 0.05% Tween-20) twice, then with buffer WB4 for 30 min at 37 °C, and three times again with PBS-T. Probe ligation in RM2 was conducted for 2 h at 37 °C, and the section was washed three times with PBS-T, then rolling circle amplification in RM3 was conducted for 18 h at 30 °C. Following PBS-T washes, all rolling circle products (RCPs) were hybridized with LM (Cy5-labelling mix with DAPI) for 30 min at room temperature, the section was washed with PBS-T and dehydrated with 70% and 100% ethanol. The hybridization chamber was removed and the slide mounted with SlowFade Gold Antifade Mountant (S36937, Thermo). Imaging of Cy5-labelled RCPs at this stage acted as a quality control step to confirm RCP (‘anchor’) generation and served to identify spots during decoding. Imaging was conducted using a Perkin Elmer Opera Phenix Plus High-Content Screening System in confocal mode with 1-μm *z*-step size, using a 63× (NA 1.15, 0.097 μm pixel^−1^) water-immersion objective. For channels: DAPI (excitation of 375 nm and emission of 435–480 nm), Atto 425 (excitation 425 nm and emission 463–501 nm), Alexa Fluor 488 (excitation 488 nm and emission 500–550 nm), Cy3 (excitation 561 nm and emission 570–630 nm) and Cy5 (excitation 640 nm and emission 650–760 nm). Following imaging, each slide was de-coverslipped vertically in PBS (gently, with minimal agitation such that the coverslip ‘fell’ off to prevent damage to the tissue). The section was dehydrated with 70% and 100% ethanol, and a new hybridization chamber was secured to the slide. The previous cycle was stripped using 100% formamide (AM9342, Thermo), which was applied fresh each minute for 5 min, then washed with PBS-T. Barcode labelling was conducted using two rounds of hybridization, first an adapter probe pool (AP mixes AP1-AP6, in subsequent cycles), then a sequencing pool (SP mix, customized with Atto 425), each for 1 h at 37 °C with PBS-T washes in between and after. The section was dehydrated, the chamber removed, and the slide mounted and imaged as previously described. This was repeated another five times to generate the full dataset of seven cycles (anchor and six barcode bits).

### Whole-mount immunostaining, tissue clearing and image analysis

Specimens were decalcified by incubation during 1 week in EDTA 0.5 M under agitation at room temperature. The solution was renewed halfway through the incubation period. The samples were washed twice in PBS 1X during 1 day. Samples were dehydrated for 1 h at room temperature in ascending concentrations of methanol in H_2_O (20%, 40%, 60% and 80%). Then, samples were placed overnight under white light (11 W and 3,000 K°) and rolling agitation (004011000, IKA) with a 6% hydrogen peroxide solution in 100% methanol. Samples were rehydrated for 1 h at room temperature in descending concentrations of methanol (80%, 60%, 40% and 20%), washed twice and blocked in 0.2% PBS-gelatin with 0.5% Triton X-100 (PBSGT) solution during 1 week. Samples were transferred to a solution containing the primary antibodies (osterix, 1/500; ab209484, Abcam and collagen2, 1/500; ab185430, Abcam) diluted in PBSGT and were incubated at 37 °C with agitation at 20 rpm for 14 days. This was followed by six washes of 1 h in PBSGT at room temperature. Next, secondary antibodies were diluted in PBSGT and passed through a 0.22-μm filter. Samples were incubated at 37 °C in the secondary antibody solution for 7 days and washed six times during 1 h in PBSGT at room temperature.

The iDISCO+ protocol was used to clear the samples^64^. Samples were placed in TPP (Techno Plastic Products) tubes, dehydrated for 1 h in methanol (20%, 40%, 60%, 80% and 100% (2x)) under rotating agitation (SB3, Stuart). Methanol volumes were equal to about five times the sample volume. The samples were next incubated in a solution of 67% DCM and 33% MeOH overnight followed by 100% DCM for 30 min at room temperature on a rotator, then put in 100% DBE. Cleared samples were imaged with a Blaze light-sheet microscope (Miltenyi Biotec) equipped with sCMOS camera 5.5MP (2,560 × 2,160 pixels) controlled by Imspector Pro 7.5.3 acquisition software (Miltenyi Biotec). The light sheet, of 4 µm thickness, was generated by lasers at four different wavelengths (488 nm, 561 nm, 639 nm and 785 nm). 1× or 4× objectives with different magnification lenses of ×0.6, ×1 and ×1.66 were used. Samples were supported by a sample holder from Miltenyi and placed in a tank filled with DBE and illuminated by the laser light sheet from one or both sides depending on the size of the samples. LightSpeed Mode was used during acquisition to acquire these images in a reasonable time and at a suitable resolution. Mosaics of 3D image tiles were assembled with an overlap of 10% between the tiles. The images were acquired in a 16 bits TIFF format. Images were initially processed using MACS iQ View Software, which performed automatic alignment of the tiles. Stack images were converted to imaris file (.ims) using ImarisFileConverter. To isolate a specific structure in Imaris, we used the surface tool with manual selection, and then used the surface to mask the image. Images and videos were taken by using either the function Snapshot and Animation in Imaris. Adobe Photoshop (v25.2) was used to colour the suture areas.

### Multiplexed smFISH

Cryosections were processed using a Leica BOND RX to automate staining with the RNAscope Multiplex Fluorescent Reagent Kit v2 assay (Advanced Cell Diagnostics and Bio-Techne), according to the manufacturers’ instructions. Probes can be found in Supplementary Table 7. Before staining, fresh frozen sections were post-fixed in 4% paraformaldehyde in PBS for 45 min at 4 °C, then dehydrated through a series of 50%, 70%, 100% and 100% ethanol for 5 min each. Following manual pre-treatment, automated processing included digestion with Protease III for 15 min before probe hybridization. Tyramide signal amplification with Opal 520, Opal 570 and Opal 650 (Akoya Biosciences), TSA-biotin (TSA Plus Biotin Kit, Perkin Elmer) and streptavidin-conjugated Atto 425 (Sigma-Aldrich) was used to develop RNAscope probe channels. Stained sections were imaged as for ISS above.

### Flow cytometry cell sorting

We applied whole-cell dissociation of fresh donor tissue as previously described^5^. Before cell extraction, the sample tissues (approximately 9 PCW shoulder joints) were dissected to obtain bone samples, and soft tissues were microdissected away. The resultant cell suspension was stained with DAPI (Invitrogen) for live-viability, FGFR3 antibody (1:50; MA5-38521, Thermo Fisher Scientific) and TACR3 antibody (1:50; BS-0166R, Thermo Fisher Scientific), and secondary antibodies. DAPI-positive singlet cells were gated for DAPI staining by FACS using a BigFoot Spectral Cell Sorter (Thermo Fisher Scientific) and its proprietary software. Sequential gating for FGFR3 and TACR3 was then conducted to identify double-positive cells. Positive controls for FGFR3 and TACR3 were conducted using human peripheral blood mononuclear cells, and unstained cells were used as negative controls.

### Image-based ISS decoding

We used the ISS decoding pipeline outlined in Li et al.^65^. This pipeline consists of five distinct steps. First, we performed image stitching using Acapella scripts provided by Perkin Elmer, which generated two-dimensional maximum intensity projections of all channels for each cycle. Next, we used Microaligner^66^ (v1.0.0) to register all cycles based on DAPI signals using the default parameters. For cell segmentation, we utilized a scalable algorithm that leverages CellPose^67^ (v3.0) as the segmentation method. The expected cell size is set to 70 pixels in diameter and further expanded 10 pixels to mimic the cytoplasm. To decode the RNA molecules, we used the PoSTcode algorithm^68^ (v1.0) with the following parameters: rna_spot_size = 5, prob_threshold = 0.6, trackpy_percentile = 90 and trackpy_separation = 2. Furthermore, we assigned the decoded RNA molecules to segmented cells using STRtree (v2.0.6) and subsequently generated AnnData objects following the approach described by Virshup et al.^69^. Finally, only cells with more than four RNA molecules were kept for downstream analysis.

### Visium processing and library preparation

Visium CytAssist Spatial Gene Expression for Fresh Frozen (10x Genomics) was performed following the manufacturer’s protocol. Regions of interest were selected based on the presence of microenvironments of bone formation relevant to the droplet data (for example, coronal suture) and aligned to the CytAssist machine gasket accordingly. Images were captured using a Hamamatsu S60 slide scanner at ×40 magnification before conducting the Visium CytAssist protocol for subsequent alignment. Libraries were mapped with SpaceRanger (10X Genomics).

### Single-nucleus isolation and library preparation

Single nuclei were isolated from fresh frozen samples through cryosectioning followed by mechanical dissociation as described in previous work^70^. In brief, 10-μm sections were homogenized in homogenization buffer (250 mM sucrose, 25 mM KCl, 5 mM MgCl_2_, 10 mM Tris-HCl, 1 mM dithiothreitol, 1× protease inhibitor, 0.4 U μl^−1^ RNaseIn, 0.2 U μl^−1^ SUPERaseIn and 0.1% Triton X-100 in nuclease-free water) using a glass Dounce tissue grinder set (Merck). Samples were dissociated with 10–20 strokes of a loose pestle ‘A’ followed by 10 strokes of a tight pestle ‘B’ when tissue fragments remained. The resulting mixture was passed through a 50-μm cell strainer, followed by centrifugation (500*g* for 5 mins), the pellet was then resuspended in 300 μl of storage buffer (1× PBS, 4% BSA and 0.2 U μl^−1^ Protector RNaseIn) and passed through a 20-μm cell strainer. Nuclei were visualized and assessed for viability under microscopy following staining with trypan blue solution and were further processed for 10X Genomics Chromium Single Cell Multiome ATAC + Gene Expression according to the manufacturer’s protocol. Nucleus suspensions were loaded with a targeted nuclei recovery of 16,000 droplets per reaction. For some of the nucleus samples, mixtures of samples from different sample donors were pooled within one reaction and later demultiplexed by genotype. Quality control of cDNA and final libraries was done using Bioanalyzer High Sensitivity DNA Analysis (Agilent). Libraries were sequenced using a NovaSeq 6000 (Illumina) with a minimum sequencing depth of 20,000 read pairs per droplet.

### Data preprocessing

Sequencing data were aligned to the human reference genome (GRCh38-2020-A-2.0.0) using CellRanger-ARC software (v2.0.0). The called barcodes from 10X Multiome lanes with pooled genotypes from multiple sample donors were demultiplexed per genotype using BAM outputs through Souporcell (v2.0)^71^. Subsequently, the Souporcell outputs were clustered by genotype for metadata assignment to each barcode. Visium data were mapped to SpaceRanger (v1.1.0) using default input settings, and low-resolution CytAssist images were aligned to hi-resolution microscopy images of the processed slides using 10X Genomics LoupeBrowser (v7.0) according to capture frame marker regions. For gene expression data, SoupX (v1.6.0)^72^ was applied to remove background ambient RNA. For CellRanger-ARC called matrices that contained more than 16,000 droplets (exceeding the number expected from targeted droplet recovery), we increased the estimated global rho value by 0.1 to account for the potential of additional ambient RNA. Droplets were filtered for more than 200 genes and less than 5% mitochondrial and ribosomal reads. Doublet removal is described below. For single-cell ATAC-seq, we applied ArchR^73^ (v1.0.2) to process the outputs from CellRanger-ARC. Initial per-droplet quality control was performed considering the number of unique nuclear fragments, signal-to-background ratio and the fragment size distribution. Moreover, droplets with transcription start site enrichment score < 7 and number of fragments < 1,000 were removed. Doublets were discarded using the default settings. Initial clustering was performed at a resolution of 0.2 with the top 40 dimensions from iterative latent semantic indexing. Then, pseudo-bulk replicates were made for each broad cluster per region from the initial clustering results. Peak calling (501-bp fixed-width peaks) was performed based on pseudo-bulk coverages by MACS2 (v2.2.7.1). Then, a cell-by-peak count matrix was obtained and exported. We applied muon^74^ (v0.1.2) for normalization, latent semantic indexing dimension reduction and clustering analysis using BBKNN^75^ (v1.5.1) to correct for batch effects from anatomical regions and sample donors to obtain an ATAC embedding. Gene scores based on chromatin accessibility around gene bodies were calculated. We then applied MultiVI^76^ (via scVI v0.6.8) to construct a joint embedding for snRNA-seq and single-cell ATAC-seq. We also applied EmptyDropMultiome^77^ (v1.0.0) to repeat droplet calling to identify nucleus-containing droplets in our Multiome data to reduce the ambient RNA noise (‘soup’). By generalizing EmptyDrops to the multi-omic setting, we used the smallest droplets to create an RNA and an ATAC ambient RNA ‘soup’ profile, and then tested each droplet for statistical deviations from each of these two profiles, retaining only droplets that were statistically significantly different from the soup profile.

### Doublet detection

All potential doublets detected in both RNA and ATAC modalities were removed from our data. For RNA data, Scrublet (v0.2.3)^78^ was applied to estimate doublet probability, and a score of more than 0.3 was used as a cut-off value. To apply a stringent doublet threshold, we conducted an adapted Scrublet workflow as previously described^79^. In brief, per-droplet Scrublet scores were first determined for CellRanger-ARC count matrices from each 10X Multiome (gene expression) lane independently. The droplets were then overclustered through the standard scanpy workflow using default parameters up to Leiden clustering. Each individual cluster was further clustered. A per-cluster median of Scrublet scores was computed. A normal distribution of doublet score, centred at the score median with a standard deviation estimated from the median absolute deviation, was used to compute *P* values for each of the clusters. After false discovery rate adjustment using Benjamini–Hochberg correction, a *P* > 0.65 was deemed as a cut-off value of good-quality cells, as doublets were significant outliers. For ATAC data, we first applied doublet detection methods from ArchR to remove putative ATAC doublets. In addition, homotypic and heterotypic doublets were characterized by running AMULET (v1.1.0) on individual snATAC-seq libraries, and droplets with *q* < 0.01 were removed.

### Droplet cluster annotation

We adopted a hierarchical clustering approach by first conducting Leiden clustering on the global integrated scVI (v0.9.1; hidden layers = 256, latent variables = 52, dispersion = ‘gene-batch’) RNA embeddings to obtain broad clusters. To validate these, we used Celltypist to train a model on cell states in the embryonic limb bud^5,80,81^, and transferred labels onto our embedding for inspection. We utilized this information in addition to canonical marker genes to annotate broad clusters and subset sublineages. For sublineages (chondrocytes, fibroblasts, osteogenesis-related clusters, Schwann cells, immune cells and endothelial cells), we further embedded each subset using scVI (hidden layers = 256, latent variables = 52 and dispersion = ‘gene-batch’) and conducted Leiden clustering (resolution = 0.6), followed by differentially expressed gene (DEG) analyses (method = ‘wilcoxon’) to obtain cluster markers. We additionally utilized the inferred spatial location of cell states (described below) to inform annotations.

### Differential abundance testing

We applied the Python implementation of the MILO package (v0.1.1) for differential abundance testing (http://github.com/emdann/milopy)^82^. We used the scVI latent representation to create a *k*-nearest neighbour graph of droplets in the relevant compartment and subsequently applied milopy to allocate droplets to overlapping neighbourhoods, with these droplets originating from multiple samples (brc_code). Each neighbourhood was then annotated as a cluster based on majority voting. We binarized values for anterior–posterior positions and calvarium-appendicular covariates to allow testing across these variables. We then determined log fold-change values for differential abundance and false discovery rate values based on the Bejanmini–Hochberg correction.

### Spatial mapping using Cell2location

We performed Cell2location (v0.1.4) for deconvolution of Visium CytAssist voxels using our annotated Multiome data as inputs. Sample donor was used as the batch variable, and each library was considered a covariate in the regression model. For spatial mapping, we estimated 30 cells per voxel based on histological data, and set a hyperparameter detection alpha value of 20 for per-voxel normalization.

### ISS-Patcher

ISS-Patcher is a package for approximating features not experimentally captured in low-dimensional data based on related high-dimensional data. It was developed as an approach to approximate expression signatures for genes missing in ISS data using matched snRNA-seq data as a reference in this study. First, a shared feature space between both datasets was identified by subsetting the 155–158 genes present in the ISS pool, followed by separate normalization to median total cell counts, log-transformation and *z*-scoring for both modalities. Then, the 15 nearest neighbours in scRNA-seq space were identified for each ISS cell with the Annoy Python package, and the genes absent from ISS were imputed as the average raw counts of the scRNA-seq neighbours.

### Visium axis annotation using OrganAxis

Our Visium cranium sample was annotated with TissueTag^8^ using a semi-automatic mode to generate a one-dimensional maturation axis. Regions of the developing bone were first manually annotated based on haematoxylin and eosin features. Tissue regions that did not include bone-forming niches were excluded from annotation. The annotation categories that were stored included multiple regions of the coronal suture (level 0 to level 2 annotation), stemming from the central-most portion, an osteogenic front (level 3 annotation) with histological features of osteoprogenitors and osteogenic zones (level 4 to level 7 annotation) from the emergence of histological osteoblasts. All annotations were saved as TissueTag output format, which logs the annotation resolution, the pixels per micrometre and the pixel value interpretation of annotation names (for example, 0 = ‘suture’) and colours (for example, ‘osteogenic front’: ‘red’). To robustly and efficiently migrate TissueTag annotations to the Visium objects, we first transferred TissueTag annotations from pixel space to a high-resolution hexagonal grid space (15-µm spot diameter and 15-µm point-to-point centre distance with no gap between spots) based on the median pixel value of the centre of the spot (radius/4) in the annotated image. Next, to generate continuous annotations for Visium data, for each spot in the hexagonal high-resolution grid, we measured the mean Euclidean distance to the ten nearest points from each annotated structure in the level 0 annotation and the distance from the closest point for structures in level 1 annotation. All annotations were mapped to the Visium spots by proximity of the spot annotation grid to the nearest corresponding spot in the Visium array.

### GRN analysis

The SCENIC+^83^ (v1.0.0) pipeline was used to predict transcription factors and putative target genes as well as regulatory genomic regions with binding sites. The fragment matrix of peaks called with MACS2 and processed within ArchR^73^ together with the corresponding RNA count matrix were used as inputs. Meta-cells were created by clustering droplets into groups of around 10–15 droplets based on their RNA profiles and subsequent aggregation of counts and fragments. The pipeline was applied to subsets of the dataset corresponding to individual lineages: first, CisTopic (pycistopic v1.0.2) was applied to identify region topics and differentially accessible regions from the fragment counts as candidate regions for transcription factor binding. CisTarget (pycistarget v1.0.2) was then run to scan the regions for transcription factor-binding sites, and GRNBoost2 (arboreto v0.1.6)^84^ was used to link transcription factors and regions to target genes based on co-expression or accessibility. Enriched transcription factor motifs in the regions linked to target genes were used to construct transcription factor–region and transcription factor–gene regulons. Finally, regulon activity scores were computed with AUCell based on target gene expression and target region accessibility, and regulon specificity scores derived from them. Networks of transcription factors, regions and target genes (enhancer-driven GRNs) were constructed by linking individual regulons. Transcription factor–enhancer–gene links for all subsets (osteogenesis, chondrogenesis, fibrogenesis, early joint progenitors, immune and Schwann) can be found in Supplementary Table 9.

### Trajectory analysis

For pseudotime trajectory construction in the osteogenic subcompartment, non-cycling droplets were subsetted, and X_scVI was used as projections for palantir to obtain multiscale diffusion space. A neighbourhood graph was generated on the diffusion space using sc.pp.neighbors, and the first two principal components were used as initial positions to create ForceAtlas2 embeddings using sc.tl.draw_graph. scFates^85^ (v1.0.3) was used to predict a principle graph that captures the differentiation path. The force-directed embeddings and principle graph were exported into R, and monocle3 (v1.0.0)^86,87^ was used to compute differentially expressed genes along pseudotime using a graph-based test (morans’ I)^87,88^, which allows identification of genes upregulated at any point in pseudotime. The results were visualized with heatmaps using the complexHeatmap (v2.6.2)^89^ and seriation (v1.3.0)^90^ packages, after smoothing gene expression with smoothing splines in R (smooth.spline; d.f. = 12). Velocity analysis^91^ was performed using scvelo^92^ (v0.2.3). Spliced and unspliced read counts were computed with velocyto (v0.17.17) from the unprocessed data, before using scvelo.pp.moments, scvelo.tl.velocity and scvelo.tl.velocity_graph to compute velocities for the preprocessed droplets. cytoTRACE^93^ was used (through the CellRank^94^ (v2.0.2) implementation) to obtain another prediction of directionality, independent of RNA velocity (based on the assumption that the number of expressed genes decreases throughout differentiation).

### Cavitation enrichment score

To approximate the timing of cavitation onset, we computed a cavitation enrichment score using sc.tl.score_genes() in scanpy on a specific gene set within the mesenchymal and muscle compartments of the hip, shoulder and knee joints comprising *CD44*, *HAS2*, *ABCC5*, *HMMR*, *MSN* and *UDPGD*, derived from literature and Gene Ontology terms, which encompass hyaluronan biosynthetic processes and hyaluronan synthase activity. We excluded genes with low expression levels in our data, such as *HAS3*. For pathway analysis, we retrieved gene sets corresponding to all 18,640 Gene Ontology terms, and computed the correlation between their enrichment scores and cavitation enrichment scores.

### In silico transcription factor perturbations

CellOracle^95^ (v0.12.0) was used with the osteogenesis trajectory created with scFates^85^, and the regulons predicted with SCENIC+^83^ for the same cells were imported into CellOracle as a base GRN. Cells were aggregated into meta-cells of 10–15 cells, and linear models explaining transcription factor from target gene expression were fitted with CellOracle per cell cluster. Regulon-based transcription factor perturbation vectors were inferred using the cell cluster-specific models to predict effects of transcription factor overexpression and knockout. Diffusion pseudotime^96^ was then computed for intramembranous and endochondral ossification lineages separately by selecting corresponding starting points. The pseudotime gradients were used to derive pseudotime-based differentiation vectors, and the pseudotime-perturbation vector cross-product was computed to obtain perturbation scores. These perturbation scores indicate whether the in silico perturbation of a transcription factor is consistent with or opposes differentiation along a lineage (osteogenesis). The simulations were carried out systematically, overexpressing and knocking out all transcription factors in the GRN. For each transcription factor and condition, the perturbation scores were then averaged per cell cluster and summarized in a table to screen for transcription factors promoting or inhibiting osteogenesis.

### fGWAS analysis

fGWAS analysis^97^ was applied to identify disease-relevant cell clusters as described in detail^55^ (https://github.com/natsuhiko/PHM). The model makes use of full summary statistics from GWAS, linking single-nucleotide polymorphisms (SNPs) to genes, and captures a general trend between gene expression and disease association of linked loci for each cell cluster. At the same time, the model also corrects for linkage disequilibrium and other relevant factors. We used full GWAS summary statistics obtained from the EBI GWAS Catalog, open targets, and knee and hip osteoarthritis as well as total knee and hip replacement from ref. ^62^ (https://msk.hugeamp.org/downloads.html; Supplementary Table 8).

### SNP2Cell

We used a network propagation^98^ approach to integrate GWAS summary statistics and cell cluster marker gene-based scores for prioritizing disease-relevant and cell cluster-specific subunits of our transcription factor network. First, scores per SNP were computed from downloaded summary statistics and weighted by linkage disequilibrium. Then, the scores were mapped to a GRN, here an enhancer-driven GRN computed with SCENIC+ for the corresponding lineage. As the used networks contain transcription factors and target genes, and also regions with transcription factor-binding sites as nodes, SNP scores were mapped to both genes and regions, representing distal regulatory elements. The scores were then propagated across the network using a random walk with restart (or personalized page rank) process. This integrates the contribution of individual SNPs, with signals converging around relevant network nodes. The procedure was repeated with 1,000 randomly permuted scores to compute permutation-test results and *z*-scores. Next, differential expression-based marker gene scores for each cell cluster were propagated in the same way, resulting in cell cluster specificity scores for each network node. The SNP and expression-based scores were then combined per cell cluster (as in ref. ^99^) by using the minimum for each node. The final scores were thresholded, and the resulting connected components were obtained as enriched subnetworks. The method has been compiled into a tool that we called SNP2Cell, which is available at https://github.com/Teichlab/snp2cell.

### Cell–cell interactions

Ligand–receptor interactions were inferred using ‘cpdb_analysis_method.call’ in CellPhoneDB (v4.0.0). We included genes expressed in more than 10% of cells within each cluster. Inferred interactions with a *P* > 0.001 were removed. We used NicheNet (v1.1.1) to identify different interactions between endochondral and intramembranous niches. We first calculated DEGs of osteogenic clusters and tip cells across the two niches using the Wilcoxon test implemented in Seurat, and minimum log fold change per cluster was used to summarize the differentially expressed ligands and receptors. The top 1,000 DEGs were used to calculate ligand activities. We prioritized the ligand–receptor links using default settings. The top ten ligands and their top-scoring receptors were visualized using heatmaps.

### Drug2Cell analysis

Drug and target gene information for humans (*Homo sapiens*) were gathered from the ChEMBL database (https://www.ebi.ac.uk/chembl/). For the teratogenic drugs targeting, we searched the clinically approved molecules that target genes encoding their reported targets and curated a list of 65 clinically approved drugs from the chEMBL database, which carried warnings of teratogenicity (Supplementary Table 6). Drug scores were calculated as previously described^58^. Subsequently, we introduced drug categories for each drug according to broad clinical utility. The Drug2Cell Python package is available at GitHub (https://github.com/Teichlab/drug2cell).

### CellHint label harmonization

First, fastq files from the Zhang et al.^5^ dataset were remapped using STARSOLO to a common genome reference (GRCh38-2020-A-2.0.0) as per the workflow performed for the Multiome data. Cellbender was applied to remove background counts represented as simulated ambient RNA. We intersected this matrix with barcodes from the post-quality control counts matrix from Zhang et al., and scVI was then used to integrate this with our snRNA-seq data, accounting for categorical covariates of sample donor and droplet technology (cell or nuclei), as well as continuous variables of total counts, the percentage of ribosomal and mitochondrial counts, and cell cycle scores ‘S_score’ and ‘G2M_score’ computed using the scanpy package. Latent variables obtained from this were then used to determine neighbourhoods followed by dimensionality reduction in UMAP. Cluster labels from Zhang et al. were then used as labels for CellHint harmonization in the cellhint.harmonize() alignment function. Cellhint.treeplot() was used to examine and semi-automatically align the labels across the two datasets. Gene expression profiles of marker genes were used to verify alignment of clusters across the two datasets.

### Reporting summary

Further information on research design is available in the Nature Portfolio Reporting Summary linked to this article.

## Online content

Any methods, additional references, Nature Portfolio reporting summaries, source data, extended data, supplementary information, acknowledgements, peer review information; details of author contributions and competing interests; and statements of data and code availability are available at 10.1038/s41586-024-08189-z.

## Supplementary information

Supplementary Methods and Discussion Details of methodology on functional GWAS enrichment analysis, and RNA spot detection analysis. Additional discussion text on cell extraction bias and on each sub-heading section of the main results.

Reporting Summary

Supplementary Figures

Supplementary Tables

Supplementary Video 1 **3D rendering of Light sheet fluorescence microscopy of the 8.5 PCW whole-embryo and cranium**. Segmentation demonstrating cranial osteogenesis, with purple labelling demonstrating Osterix (*SP7*), and blue label demonstrating collagen 2a1 (*COL2A1*) staining.

Supplementary Video 2 **3D rendering of Light sheet fluorescence microscopy of the 10 PCW cranium**. Segmentation demonstrating cranial osteogenesis, with purple labelling demonstrating Osterix (*SP7*), and blue label demonstrating collagen 2a1 (*COL2A1*) staining.

## Source data

Source Data Fig. 3

Source Data Fig. 5

Source Data Fig. 6

## Data Availability

High-throughput raw sequencing data in this study are available from ArrayExpress (www.ebi.ac.uk/arrayexpress) with the accession number E-MTAB-14385. Processed snRNA–scATAC-seq, Visium and ISS data are available for visualization and can be downloaded from https://developmental.cellatlas.io/skeleton-development. Source data are provided with this paper.

## Code availability

ISS-Patcher is available at https://github.com/Teichlab/iss_patcher. SNP2Cell is available at https://github.com/Teichlab/snp2cell. The custom code for the other analyses performed in this study is available at GitHub (https://github.com/Teichlab/skeletal_dev_atlas).
