# Supplementary material for: A multi-omic atlas of human embryonic skeletal development
Source: Nature. 2024 Nov 20;635(8039):657–67. doi: 10.1038/s41586-024-08189-z (PMC11578895; doi:10.1038/s41586-024-08189-z)
Supplement: Supplementary file 1 — Details of methodology on functional GWAS enrichment analysis, and RNA spot detection analysis. Additional discussion text on cell extraction bias and on each sub-heading section of the main results. [file 41586_2024_8189_MOESM1_ESM.docx]

# Supplementary Methods and Discussion: A Multi-omic Atlas of Human Embryonic Skeletal Development

Ken To*^1^, Lijiang Fei*^1^, J. Patrick Pett*^1^, Kenny Roberts^1^, Raphael Blain^2^, Krzysztof Polański^1^, Tong Li^1^, Nadav Yayon^1,3^ Peng He^1,3,4^, Chuan Xu^1^, James Cranley^1,5^, Madelyn Moy^1^, Ruoyan Li^1^, Kazumasa Kanemaru^1^, Ni Huang^1^, Stathis Megas^1,6^, Laura Richardson^1^, Rakesh Kapuge^1^, Shani Perera^1^, Elizabeth Tuck^1^, Anna Wilbrey-Clark^1^, Ilaria Mulas^1^, Fani Memi^1^, Batuhan Cakir^1^, Alexander V. Predeus^1^, David Horsfall^1^, Simon Murray^1^, Martin Prete^1^, Pavel Mazin^1^, Xiaoling He^7,8^, Kerstin B. Meyer^1^, Muzlifah Haniffa^1,9,10^, Roger A. Barker^7,8^, Omer Bayraktar^1^, Alain Chedotal^2,11,12^, Christopher D. Buckley^13^, Sarah A. Teichmann^#1,5,6,8^

*These authors contributed equally
^#^corresponding author: sat1003@cam.ac.uk

^1^Wellcome Sanger Institute, Wellcome Genome Campus, Hinxton, UK
^2^Sorbonne Université, INSERM, CNRS, Institut de la Vision, Paris, France.
^3^European Molecular Biology Laboratory, European Bioinformatics Institute (EMBL-EBI), Wellcome Genome Campus, Cambridge, UK
^4^Department of Pathology, University of California, San Francisco, San Francisco, US
^5^Department of Medicine, University of Cambridge, Cambridge, UK
^6^Cambridge Centre for AI in Medicine, Department of Applied Mathematics and Theoretical Physics, Cambridge, UK
^7^John van Geest Centre for Brain Repair, Department of Clinical Neurosciences, University of Cambridge, Cambridge, UK
^8^Wellcome-MRC Cambridge Stem Cell Institute, University of Cambridge, Cambridge, UK
^9^Newcastle University, Biosciences Institute, Newcastle University, Newcastle upon Tyne, UK
^10^Department of Dermatology and NIHR Newcastle Biomedical Research Centre, Newcastle Hospitals NHS Foundation Trust, Newcastle upon Tyne, UK
^11^Institut de pathologie, groupe hospitalier Est, hospices civils de Lyon, Lyon, France.
^12^University Claude Bernard Lyon 1, MeLiS, CNRS UMR5284, INSERM U1314, Lyon, France.
^13^Kennedy Institute of Rheumatology, University of Oxford, Oxford, UK

# Supplementary Methods

## **Functional GWAS enrichment analysis**

To explore potential causal links in foetal skeletal cell states for adult complex traits, we conducted fGWAS (Fig. 1c), which integrates full GWAS summary statistics with single cell epigenetic and transcriptomic signatures and computes cell-type specific enrichments for phenotypes. We used summary statistics for GWAS of total knee replacement (TKR), total hip replacement (THR) as a surrogate phenotype for knee, and hip osteoarthritis, respectively. This is consistent with definitions of OA across GWAS of knee and hip OA.

**RNA Spot Detection**RNA spot detection was performed using Spotiflow[^1^](https://paperpile.com/c/v7jyQI/0c8Vw), a deep-learning based peak detection algorithm, which identifies individual RNA spots within the image. Subsequently, CellPose[^2^](https://paperpile.com/c/v7jyQI/SAkFF), a deep-learning based cell segmentation tool, was used to segment individual cells from the background. This step enabled assignment of detected RNA spots to their respective segmented cells, allowing for single-cell resolution and spatial analysis of gene expression. To quantify regional differences in gene expression, three regions of interest were manually annotated on OMERO[^3^](https://paperpile.com/c/v7jyQI/LUzVM): (1) Subchondral regions containing putative *PAX7*-expressing chondrocytes, (2) Positive Control regions containing muscle, and (3) Negative Control regions containing neither. Expression levels of target RNAs were then reflected using RNAscope, with differential expression patterns observed across these distinct anatomical regions. In addition to the visualization of gene expression profiles in each region, we generated boxplots to compare expression levels of different genes across the annotated regions.

# Supplementary Discussion

## **Cell extraction bias**

We analysed variations in cell composition related to sampling locations and time points using a Poisson linear mixed model. Both anatomical region and time points influenced the abundance of different cell clusters. For instance, region-specific cell clusters such as perisuture dura and meningeal dura were enriched in the calvaria and skull base, while perichondrium and synovial fibroblasts were enriched in appendicular joints (Supplementary Fig. 1).

This variance can be attributed to two parts: intrinsic cellular differences related to region-specific or stage-specific cell types, and single-cell extraction biases. Here, we hypothesise that the spatial data probably more accurately captures cellular intrinsic differences and so the cell type abundance difference between spatial and single-nuclei data can be viewed as representative of the single-cell extraction bias. For the ISS datasets, we first subsetted anatomical areas corresponding to snRNA-seq data from ISS slides. Then, we inferred cell type identity for the segmented cell in the ISS slides using the snRNA-seq datasets. To reduce biases from snRNA-seq's cell type abundance, we used the geosketch method to downsample single-nuclei datasets multiple times, then used ISS-patcher to infer cell types for each segmented cell. We then evaluated cell type distribution differences using four metrics across regions and time: PCC (pearson correlation coefficient) and SSIM (structural similarity index) for quantifying similarity, JS (Jensen–Shannon divergence) and RMSE (root mean square error) for difference (RMSE/JS) between the predicted results and the ground truth.

When comparing single nuclei and ISS spatial data cell abundance in knee and shoulder samples, tissues at earlier time points (5.7 PCW and 6.7 PCW, respectively) showed more similar composition than later time points, which indicates less single nuclei exaction bias in earlier time points (Supplementary Fig. 1) We hypothesise this may relate to tissue property changes, such as stiffness and ECM as the reviewer suggested. Notably, there were significant extraction differences observed for chondral progenitors of knee at 7.3 PCW and chondral progenitors and myoprogenitors for shoulder at 7.3 PCW. When comparing single nuclei and visium spatial data cell abundance in the calvarial tissues, later time points (11PCW) showed greater correlation in composition across droplet and spatial modalities for similarity scores, but then demonstrated inconsistent results as later time points (11PCW) also showed greater dissimilarity. We hypothesise that this may be due to the less comparable visium data, which does not have single-cell resolution, and the lower number of samples at the earlier time points (<9PCW). We also include a table (Supplementary Table. 10) to demonstrate targeted and post-qc droplet recovery.

**Cellular taxonomy of joint development**

To facilitate the reconstruction of osteochondral lineage development, we profiled nuclei-droplets, and sampled across the first trimester. Prior human single-cell (scRNA-seq) atlases of developing limbs[^4–6^](https://paperpile.com/c/v7jyQI/1Hoxn+u9fyX+ICKjJ) profiled whole cells and captured low numbers of maturing osteoblast and *COL10A1*+ hypertrophic chondrocyte transcriptomes. Label harmonization (see methods) of our dataset against published single-cell data demonstrated comparatively more diverse chondrogenic and osteogenic populations in our data, enriching toward the end of the first trimester (Extended Data Fig. 2). We theorise that larger cells such as osteoblasts and hypertrophic chondrocytes may be comparatively less amenable to whole-cell profiling, due to upper limits of microfluidic channel size [^7–9^](https://paperpile.com/c/v7jyQI/iqAcj+4vU29+lYguI).

## **Zonation of the embryonic synovial joint**

We observed five populations enriched in the early appendicular joint including interzone chondrocytes (InterzoneChon; *GDF5*, *PITX2*), *HIC1*+ mesenchyme (*HIC1*+Mes; *HIC1*), fibroblast-progenitors (FibroPRO1, FibroPRO2; PI16, ) and dermal fibroblasts (DermFIB1, DermFIB2; *ADAM22*, *TWIST1*). While the co-existence of these clusters by 5-7 PCW may suggest that numerous early progenitors contribute to the chondrocyte-fibroblast lineages in the joint, it remains unclear whether they are preceded by a single master *GDF5*+ progenitor population in humans[^10^](https://paperpile.com/c/v7jyQI/frKef). To identify these progenitors, we applied differential abundance testing[^11^](https://paperpile.com/c/v7jyQI/GRfKq) (see methods) on the mesenchyme clusters across developmental time (5-11 PCW) and regions (shoulder, hip, knee) .

Within the broad InterzoneChon (*GDF5^+^ PITX2^+^*) population, except for the Early IZ population (main text), we found six other IZ subclusters. These carried lineage-related transcriptional signatures (Fibro IZ, Articular IZ, *GDF5*high IZ, Dermal IZ, Hypertrophic IZ1 and Hypertrophic IZ2) that were predicted to be preceded by this early cluster based on RNA velocity (Fig. 2a). Articular IZ was prevalent in the knee joint, whereas Fibro IZ enriched in the shoulder and hip (Fig. 2a), which may reflect earlier maturation of fibrocartilage components in the embryonic shoulder and hip, compared to the knee. In the hypertrophic IZ clusters, high *RUNX2* TF activity[^12–14^](https://paperpile.com/c/v7jyQI/o3VOJ+GQT5j+LIPgj), and low regulation of articular chondrocyte TFs (*CREB5*, *EGR1*) were observed (Fig. 2b). Hypertrophic IZ2 was predicted to differentiate from Hypertrophic IZ1 and expressed genes associated with pre-hypertrophic chondrocytes in mice (*BMP5*, *HAPLN1*, *ACAN* and *ZFHX3*)[^15,16^](https://paperpile.com/c/v7jyQI/Rj8wl+Z0kVB), suggesting they form the cartilage scaffold. Articular IZ, which was prevalent in the knee (Fig. 2a), expressed mouse articular chondrocyte progenitor markers (*COL2A1*, *ADGRG6*, *PIEZO2*, *LGR5*, *NOG*)[^17,18^](https://paperpile.com/c/v7jyQI/5cStj+GabeB), and also *ENPP1*, a negative regulator of bone and hypertrophic chondrocyte formation, suggesting a poised articular phenotype. While *GDF5*high IZ was predicted to highly express *CREB5* and *DBX2* and its target genes, which are associated with digital IZ formation regulated by *HOX* genes[^19,20^](https://paperpile.com/c/v7jyQI/guvCX+SOtqW), chondrogenesis TFs were not highly expressed, and accessibility for *RUNX2* was low. Overall, our *in vivo* data are consistent with *in vitro* observations of the propensity for human induced pluripotent stem cells (hiPSC) and mouse embryonic stem cells (mESC) derived *GDF5*+ cells to form an articular, rather than a hypertrophic phenotype[^21,22^](https://paperpile.com/c/v7jyQI/VSpGw+Y0xB4).

To investigate cavitation onset following zonation of the joint, we computed a cavitation enrichment score using a gene set associated with hyaluronan biosynthesis derived from literature[^23^](https://paperpile.com/c/v7jyQI/pWN22) and GO terms, comprising *CD44*, *HAS2*, *ABCC5*, *HMMR*, *MSN*, and *UDPGD*. We observed high cavitation score and *CD44* enrichment at 7.6 PCW in the shoulder and knee, and 8.3 PCW in the hip, which could be due to the later development of the hip (Supplementary Fig. 2). Additionally, pathways involved in the regulation of extracellular matrix assembly ranked among the top 10 correlated pathways in three regions. Conversely, cell death and apoptosis pathways showed weak correlations but positive regulation of monocyte aggregation emerged as the top-ranked pathway in all three regions (Supplementary Fig. 2).

## **Emergence of fibroblast lineages**

*TWIST1*, a known activator of postnatal fibrosis and *TWIST2* a regulator of postnatal dermal fibroblast proliferation[^24,25^](https://paperpile.com/c/v7jyQI/HaoYu+VkDQ1), showed high TF activity in DermFIB1,2, which localised to the skin region (Extended Data Fig.5g,i). Interestingly, *EN1*, a TF required for fibrosis and scarring during postnatal wound healing[^26^](https://paperpile.com/c/v7jyQI/xiwgC), was highly expressed in DermFIB1 (Extended Data Fig. 5g,h) but showed lower expression and target gene expression in the more mature DermFIB2, suggesting downregulation, consistent with observations of scarless wound healing *in utero*. No dorsal-ventral bias in gene expression was observed across these gene expression patterns in our data.

## **Formation of the cranial sutures**

We identified three early cranial progenitors (<8PCW), including facial (FacialMes) and pharyngeal mesenchyme (PArchMes) which were located in the skull base region and expressed markers of axial mesoderm (*PAX3* and *LHX8*), and a cranial mesenchyme (CranialMes) population which was abundant in the calvarium (Extended Data Fig. 4a, 5a-c).

We sought to delineate the contribution of later *RUNX2*-expressing osteogenic SutureMes1/2 populations to the bone-forming suture niche by clustering these early populations in combination with osteoblasts (*SP7*, *ALP*), osteocytes (*SOST*, *DMP1*) and preosteoblasts (*RUNX2*, *THBS2*, *POSTN*) which revealed anatomical region and age specific osteo lineage clusters (Extended Data Fig. 4a, 6a-c).

## **Trajectories of skeletal osteogenesis**

Besides CranialMes, FacialMes and LimbMes, we identified a PArchMes population, which emerged between 6-8 PCW and expressed marker genes of the first pharyngeal arch (*PAX9*, *LHX*, *DLK1*)[^27^](https://paperpile.com/c/v7jyQI/1kkYY), and may comprise mixed mesoderm and NCC origins. LimbMes expressed mesoderm markers *TBX5* and *ISL1*, which have previously been described as markers of forelimb and hindlimb early mesoderm, respectively. We did not further subset LimbMes according to anatomical regions for the trajectory analysis.

Within the two newly defined predicted trajectories for EC and IM osteogenesis, we determined dynamic expression associated with pseudotime (Extended Data Fig. 7a). Common TFs (*RUNX2*, *DLX5*, *SP7*, *SATB2*) mediating osteogenesis were upregulated toward terminal states across both pathways. SutureMes1/2 was enriched for osteogenic genes (*SPARC*, *COL1A1*, *GAS1*) early in the predicted trajectory, and signatures previously associated with suture formation and regulation in the foetal and postnatal mouse suture (*PRDM6*, *MSX1*) were most upregulated upon transition to SutureMes2[^28^](https://paperpile.com/c/v7jyQI/XbzYr). Inhibitory control of osteogenesis differed across the pathways. *LMX1B*, a common repressor, was enriched late in progenitors across both pathways whereas *TWIST1* was only enriched late in IM progenitors, signifying additional inhibitory regulation in the IM pathway. *NFATC2*, a chondro- and osteogenesis repressor[^29,30^](https://paperpile.com/c/v7jyQI/Rv7Gw+N0imC), was observed early in the IM pathway, but late in the EC pathway, potentially reflecting context and timing dependent roles in maintenance of the suture niche (IM), and cartilage primordia replacement (EC), respectively.

In IM progenitors, accessibility across activators (*RUNX2*) and repressors (*TWIST1*, *LMX1B*) was simultaneously high at early parts of the predicted trajectory, suggesting they are poised for osteogenesis but remain repressed in the early stages (Extended Data Fig. 6f). In contrast, at the transcriptome level, there was a reciprocal relationship between repressor and *RUNX2* target expression across pseudotime, suggesting critical regulation of the balance limiting osteogenesis in SutureMes1/2. In the EC pseudotime trajectory, predicted *NFATC2* activity peaked toward Preosteoblasts and then decreased, whereas *RUNX2* activity increased as Preosteoblasts formed, suggesting the *RUNX2* was critical in driving progression. The multi-omic dynamic changes in the IM pathway, in contrast to EC osteogenesis, suggest a more pronounced separation between pro-osteogenic and repressive states (Extended Data Fig. 6f).

While we did not observe evidence for intramembranous ossification in the developing human limb regions profile here, the possibility of this taking place during development can not be entirely excluded without deeper exploration in future work.

## **Angiogenesis in the osteogenic niches**

Along the spatial axis of osteogenesis in the developing frontal bone (Fig. 3d-e), *ATF4*, a regulatory gene that promotes bone angiogenesis in development[^31^](https://paperpile.com/c/v7jyQI/W3aKu), also showed increased expression in osteogenic zones, suggesting a spatial-temporal association between vascularisation and osteogenesis.

To predict distinct cell-cell interactions across the EC and IM niches, we utilised NicheNet to compare differences in inferred signalling during sprouting angiogenesis, focusing on tip cells that lead vascular sprouts. SutureMes1/2 enriched for *EFNB2*, *EFNB3*, and *BMP3*, which were associated with craniofrontonasal dysplasia and may be associated with sprouting behaviour, motility, and vessel formation (Extended Data Fig. 9b)[^32^](https://paperpile.com/c/v7jyQI/UmZ9Q). *RACK1*, which encodes an intracellular scaffold protein that promotes VEGF-FLT1-dependent cell migration[^33^](https://paperpile.com/c/v7jyQI/3AOrr), was highly expressed in tip cells in the calvaria, signifying a motile state. *CXCR4*, which promotes sprout anastomosis, was upregulated in tip cells[^34^](https://paperpile.com/c/v7jyQI/0ntiF). In EC ossification, tip cells distinctly express *CDON*, the receptor for IHH which promotes endothelial proliferation, migration, and angiogenesis *in vivo*[*^35^*](https://paperpile.com/c/v7jyQI/NqZMc). RNA-ISH showed colocalization of *EFNB2* and *EPHB1* expression, a predicted interaction between SutureMes1/2, *HHIP*+PreOB and tip cells ( Extended Data Fig. 10). This data suggests potential intercellular signalling between SutureMes1/2 and *HHIP*+PreOB, with endothelial tip cells in the IM osteogenic niche.

When investigating signalling interactions from endothelial cells toward osteogenic populations, we did not observe *NOG* expression in endothelial cells, unlike the previously described postnatal bone-associated type H vessels[^36^](https://paperpile.com/c/v7jyQI/WSsDT), which suggests possible differences in interactions between endothelial and osteoblastic cells via NOTCH signalling prenatally. Mural cells expressed *RSPO3* which was predicted to interact with LGR5, a signalling pair that had been reported to promote bone mineralization via activation of canonical WNT signalling[^37^](https://paperpile.com/c/v7jyQI/idtEf). We observed *WNT2B*, *FZD5* and *WIF1* expression in proximity on RNA-ISH (Extended Data Fig. 10).

Other lineages, including neurons have also been reported to play a role in modulating osteogenesis and angiogenesis[^38,39^](https://paperpile.com/c/v7jyQI/IZN3X+t1UWw). Neuronal development and axon guidance modules were enriched in early stages of both osteogenic pathways (Supplementary Table 4), suggesting possible involvement of chemical cues from axons in niche formation (Extended Data Fig. 9f).

## **Inference of chondrocyte origins**

To reveal the transcriptional heterogeneity of chondrocytes, we clustered chondrocytes across anatomical regions and timepoints and annotated them based on canonical marker genes (Fig. 4a, b, Extended Data Fig. 11a and Supplementary Table 2). To then explore cellular heterogeneity within spatial context, we applied ISS-Patcher and Cell2location to transfer cell state labels from our single-cell atlas to ISS and 10x Visium data (Extended Data Fig. 11b-c).

We identified known populations, including hypertrophic chondrocyte (HyperChon, *COL10A1*, *IHH*), cycling chondrocyte (CyclingChon, *MKI67*, *TOP2A*), resting chondrocyte (RestingChon, *UCMA*), interzone chondrocyte (InterzoneChon, *GDF5*, *PITX1*). In appendicular regions, we observed two subtypes of articular chondrocytes, with one population enriching for *TRPV4* and *VEGFA*, while the other was more mature, with relatively low *SOX9* expression and high *EPYC* expression. In the skull base, facial chondrocytes (FacialChon) highly expressed *PAX3*, suggesting potential origins from neural crest[^40^](https://paperpile.com/c/v7jyQI/bQo7L). Mandibular chondrocytes (MandibularChon) highly expressed *SEMA3D* in posterior regions[^41,42^](https://paperpile.com/c/v7jyQI/Hc4N7+iWHQP). ChondroPro1,2 were enriched in appendicular joints and skull, respectively, and expressed fibroblast differentiation markers (*POSTN*, *COL1A1*, *PRRX1*, and *TWIST1*), consistent with findings in early chondrocyte progenitors in mice[^43^](https://paperpile.com/c/v7jyQI/gRCcR).

We identified common and region-specific co-expressed gene modules using Hotspot[^44^](https://paperpile.com/c/v7jyQI/dBG7u) and performed gene ontology enrichment on each module (Extended Data Fig. 11d-e). Shared gene modules across regions included chondrocyte cellular structure and functional development modules as well as biomineralization, cell growth, and cartilage development modules. Interestingly, nerve related pathways were enriched both in appendicular and cranial samples in the first trimester[^45^](https://paperpile.com/c/v7jyQI/zJ7VN). In addition, chondrocytes from the skull base enriched gene modules for responding to steroid hormone, while chondrocytes from appendicular joints had specific gene modules, including glucose metabolism and cellular response to hypoxia (Extended Data Fig. 11f-g). Taken together, our data provides a broad catalogue of chondrocyte molecular profiles in multiple developing human joints.

Our work suggested the possible presence of a previously undescribed developmental chondrocyte cluster (*PAX7*+Chon), which was first represented at 7 PCW and enriched between 9-10 PCW (Fig. 4c). The *PAX7*+ Chon cluster co-expressed markers and gene modules of chondrocytes and muscle cells at both transcriptomic and epigenetic level (Supplementary data Fig. 4a), also showing overlap of genes involved in development pathways of both (Supplementary data Fig. 5a-b). The *PAX7*+Chon cluster was enriched in multiple samples from various donors across different anatomical regions (Fig. 4c and Supplementary data Fig. 5c). To reveal GRNs that govern the transcriptional identity of *PAX7*+Chon, we applied SCENIC+ and constructed a GRN based on differentially expressed transcription factors (TFs) with their predicted target genes (Supplementary data Fig. 4b). Aside from identifying myogenic and chondrogenic regulators (*PAX7*, *MYF5*, *SOX5*, *SOX9*), we also discovered core posterior axis HOX gene modules[^46^](https://paperpile.com/c/v7jyQI/yHuoj). Previous mouse studies have suggested shared origins between chondrocytes and the muscle lineage[^47–49^](https://paperpile.com/c/v7jyQI/ghq0C+06n9c+bzhvm). We therefore theorise the possibility of chondrocytes derived from muscle progenitors, noting that further work is required to investigate this.

Next, we investigated this cluster using multiple computational and experimental approaches. First, to mitigate effects of erroneous gene expression due to ambient noise, we repeated cell calling using EmptyDropsMultiome[^50^](https://paperpile.com/c/v7jyQI/9bEmr), which was developed to detect nuclei-containing droplets using single nuclei RNA and ATAC multiome data (Supplementary data Fig. 5d).

We also investigated the potential of the *PAX7*+Chon cluster arising due to doublets or multiplets formation by combining transcriptomic and ATAC doublets detection methods. We performed additional statistical tests on doublet scores from Scrublet at the transcriptomic level, and we applied AMULET[^51^](https://paperpile.com/c/v7jyQI/nzBIZ) to computationally remove doublets by counting uniquely mapped reads in gene regions at the ATAC level (see methods, Supplementary data Fig. 5e). *PAX7*+Chon also displayed transcriptional characteristics not clearly explained by doublet formation (Supplementary data Fig. 5f-g).

Next, we investigated *PAX7*+Chon through RNA in situ hybridization experiments in the knee and shoulder in samples from various developmental developmental stages (Supplementary Fig. 4c, Supplementary Fig. 6a), which was consistent with its imputed position in the multiplexed ISS data (Extended Data Fig. 11b). Through this we observed a potential transcript capture signal which was overall sparsely distributed in regions of the subchondral bone. While PAX7 and chondrocyte markers were observed to colocalise in these regions, a quantitative comparison (Supplementary Fig. 6a, Supplementary Fig. 8) demonstrated that PAX7 staining within putative PAX7+ACAN+ chondrocytes were only modestly expressed compared to background staining. Future investigations will therefore clarify whether these are a *bona fide* spatial representation of the *PAX7+*Chon population.

Future investigations will therefore clarify whether these are a *bona fide* spatial representation of the *PAX7+*Chon population. At the protein level, we purified *PAX7*+Chon using specific surface markers (*FGFR3* and *TACR3*) via fluorescence-activated cell sorting (FACS) (Supplementary data Fig. 6b-c). We isolated TACR3+ FGFR3+ populations from dissociated cells of the embryonic upper limb, which are representative of *PAX7+*chon based on transcriptional patterns from the droplet data. Deeper transcriptional profiling of these sorted populations and negative controls in future work will offer more comprehensive characterisation.

## **Developmental links to complex traits**

Complex diseases are often associated with many mutations across non-coding regions. We therefore sought after a way to integrate the GWAS signals of hip and knee OA described above across enhancers of our gene regulatory network to identify enriched TFs and pathways, while accounting for cell cluster specific effects. To this end, we developed SNP2CELL, a tool that uses gene regulatory networks as a basis to aggregate scores of individual SNPs and cluster marker genes across pathways of connected TFs (Fig. 5b), allowing cell cluster specific enrichment of sub-networks of GRNs.

Using SNP2CELL we find several enriched genes linked to cartilage function in the core network (Fig. 13a). *COL27A1* and the proteoglycan *SNORC*, are involved in chondrocyte ECM makeup and maturation[^52^](https://paperpile.com/c/v7jyQI/diAcs). *PRKCA* is a kinase that has been linked to mechano-sensing in articular chondrocytes[^53^](https://paperpile.com/c/v7jyQI/wa1iQ). Notably, *NFATC1* itself has been described as a marker of articular cartilage progenitors affecting differentiation[^54^](https://paperpile.com/c/v7jyQI/8lmvC) and also protects against OA[^55^](https://paperpile.com/c/v7jyQI/y5Je9).

In contrast, in hipOA we find more enriched genes linked to osteogenesis affecting pathways. Both canonical and non-canonical WNT signalling via Ca2+/Calcineurin/NFAT have been linked to bone formation and remodelling, affecting the balance and differentiation of both osteoblasts and osteoclasts[^56^](https://paperpile.com/c/v7jyQI/Bsh7b). Various WNT signalling inhibitors including DKK1 and FRZB have also been linked to the shaping process of the hip and osteoarthritis, which may be particularly important during foetal development[^57^](https://paperpile.com/c/v7jyQI/43Ohk).

Overall, gene set enrichment analysis (GSEA) on Gene Ontology (GO) terms featured terms related to extracellular matrix organisation, cartilage development and chondrocyte differentiation in articular chondrocytes in knee OA specifically, whereas hip Preosteoblast scores showed enrichment for inositol-phosphate, calcineurin and NFAT signalling, and cellular response to lipid (Fig. 5e), suggesting potential interplay of lipids with bone formation and osteoarthritis[^58^](https://paperpile.com/c/v7jyQI/CaAee).

## **Deciphering monogenic craniosynostosis**

Disease mechanisms that underlie craniosynostosis are reportedly linked to missense mutations and haploinsufficiency in genes that govern persistence of IM osteo-progenitor pools within the suture joints throughout the cranium[^59–61^](https://paperpile.com/c/v7jyQI/VpIBL+KnGKp+g96Mp).

To find overlaps with known genes, we filtered the DEGs from our osteogenesis trajectory to those associated with musculoskeletal (MSK) conditions, and created a global view of enriched expression and accessibility across the intersecting genes (Extended Data Fig. 13b). A high proportion of reported genes obtained from Genomics England Limited rare and common craniosynostosis panels (Supplementary Table 5) were within the top DEGs.

Among genes found through *in silico* perturbations with CellOracle, *TWIST1* and *MSX2* were also IM pseudotime-associated DEGs (Fig. 6b, Extended Data Fig. 13b), suggesting the predicted velocity shift may reflect the loss-of-function mechanism of disease pathogenesis in these TFs[^62,63^](https://paperpile.com/c/v7jyQI/fl0Yc+2zHrk). Numerous other disease-associated TFs demonstrated maximal enrichment in the Suture Zones (*IL11RA*, *SIX1*), whereas others preferentially affected more developed parts of the pseudotime trajectory (*IHH*, *ALPL*, *VLDLR*) (Fig. 6b). While we focused on delineating predicted osteolineage trajectory shifts arising from *in silico* knockout of known craniosynostosis associated genes, other cell states within the microenvironment have been reported to be affected by disease-associated mutations.

When investigating our SCENIC+-derived eGRN, *TCF12* served as a node connecting *TWIST1* to *RUNX2*, suggesting a critical co-regulatory network (Extended Data Fig. 6d). While direct transcriptional regulations of *RUNX2* by the three inhibitory TFs were predicted to be weaker than their inter-regulation, predicted regulation of *RUNX2* (Supplementary data Fig. 7) therefore likely contribute to known complex regulatory mechanisms, including protein-level interactions[^64^](https://paperpile.com/c/v7jyQI/ZHf6v). In addition, cell-extrinsic signals and control mechanisms may potentially act on the connecting nodal points such as *NFATC2*[*^65^*](https://paperpile.com/c/v7jyQI/2ztF4), governing the balance of osteogenesis.

Multi-omic information further allows predictions of enhancer-mediated regulation of transcription. This is of particular importance for monogenic bone-forming conditions such as Van Buchem disease (VBD) whereby mutation in the non-coding ECR5 enhancer of *SOST* leads to sclerosing dysplasia of bone[^66^](https://paperpile.com/c/v7jyQI/HrhJ9). We therefore constructed a regulatory network of normal development centred around *SOST* and identified the region containing the ECR5 enhancer to directly regulate *SOST* (Fig. 6e). Numerous osteogenesis-associated TFs were predicted to regulate the region containing the ECR5 enhancer (*DLX5*, *KLF2*, *KLF4*, *KLF13*, *MEF2C*). Notably, *MEF2C* has previously been shown to regulate ECR5 in mice[^67^](https://paperpile.com/c/v7jyQI/bJSWS), confirming its direct role in controlling *SOST* transcription.

Drug-target enrichment in our osteogenesis trajectory using the chEMBL database (Extended Data Fig. 14) showed that retinoid drugs, and thalidomide enriched for targets in osteocytes, consistent with observations of retinoic acid enhancing *in vitro* osteogenesis in mouse suture cells[^68^](https://paperpile.com/c/v7jyQI/pwst9), and the well-described effects of thalidomide. Aside from antihypertensives targeting the renin-angiotensin pathway (ACEi, ARB, Renin inhibitor) which enriched for targets in SutureMes1 and *HHIP+*PreOB, we also identified high enrichment for endothelin receptor antagonists (ERAs) targets specifically in SutureMes1. Mice lacking the endothelin A receptor recapitulate wide-spread cranial neural crest-related defects *in utero*[*^69^*](https://paperpile.com/c/v7jyQI/Zr6NR) and ERAs carry teratogenic effects when used in animals[^70^](https://paperpile.com/c/v7jyQI/cmUBp).

Apart from the chondrogenic and osteogenic trajectories we study, other lineage relations, for example, the potential for mouse skeletal cell states, such as chondrocytes to transdifferentiate into bone lineage cells have been reported in late gestation[^71^](https://paperpile.com/c/v7jyQI/Q0qqS), but were not observed in our study of early gestation. Further investigation focused beyond the first trimester and of postnatal bone and joints, when tissues mature, will facilitate a complete cell census of the developing skeleton and shed light on the contribution of IM and EC ossification to different regions throughout development.

# Supplementary References

1. [Mantes, A. D. *et al.* Spotiflow: accurate and efficient spot detection for fluorescence microscopy with deep stereographic flow regression. *bioRxiv* 2024.02.01.578426 (2024) doi:](http://paperpile.com/b/v7jyQI/0c8Vw)[10.1101/2024.02.01.578426](http://dx.doi.org/10.1101/2024.02.01.578426)[.](http://paperpile.com/b/v7jyQI/0c8Vw)

2. [Stringer, C., Wang, T., Michaelos, M. & Pachitariu, M. Cellpose: a generalist algorithm for cellular segmentation. *Nat. Methods* **18**, 100–106 (2020).](http://paperpile.com/b/v7jyQI/SAkFF)

3. [Allan, C. *et al.* OMERO: flexible, model-driven data management for experimental biology. *Nat. Methods* **9**, 245–253 (2012).](http://paperpile.com/b/v7jyQI/LUzVM)

4. [Zhang, B. *et al.* A human embryonic limb cell atlas resolved in space and time. *bioRxiv* 2022.04.27.489800 (2023) doi:](http://paperpile.com/b/v7jyQI/1Hoxn)[10.1101/2022.04.27.489800](http://dx.doi.org/10.1101/2022.04.27.489800)[.](http://paperpile.com/b/v7jyQI/1Hoxn)

5. [Jardine, L. *et al.* Blood and immune development in human fetal bone marrow and Down syndrome. *Nature* **598**, 327–331 (2021).](http://paperpile.com/b/v7jyQI/u9fyX)

6. [He, P. *et al.* The changing mouse embryo transcriptome at whole tissue and single-cell resolution. *Nature* **583**, 760–767 (2020).](http://paperpile.com/b/v7jyQI/ICKjJ)

7. [Zhang, B. *et al.* A human embryonic limb cell atlas resolved in space and time. *bioRxiv* 2022.04.27.489800 (2023) doi:](http://paperpile.com/b/v7jyQI/iqAcj)[10.1101/2022.04.27.489800](http://dx.doi.org/10.1101/2022.04.27.489800)[.](http://paperpile.com/b/v7jyQI/iqAcj)

8. [Jardine, L. *et al.* Blood and immune development in human fetal bone marrow and Down syndrome. *Nature* **598**, 327–331 (2021).](http://paperpile.com/b/v7jyQI/4vU29)

9. [He, P. *et al.* The changing mouse embryo transcriptome at whole tissue and single-cell resolution. *Nature* **583**, 760–767 (2020).](http://paperpile.com/b/v7jyQI/lYguI)

10. [Feng, C. *et al.* Lgr5 and Col22a1 Mark Progenitor Cells in the Lineage toward Juvenile Articular Chondrocytes. *Stem cell reports* **13**, (2019).](http://paperpile.com/b/v7jyQI/frKef)

11. [Dann, E., Henderson, N. C., Teichmann, S. A., Morgan, M. D. & Marioni, J. C. Differential abundance testing on single-cell data using k-nearest neighbor graphs. *Nat. Biotechnol.* **40**, 245–253 (2021).](http://paperpile.com/b/v7jyQI/GRfKq)

12. [Chen, H. *et al.* Runx2 Regulates Endochondral Ossification through Control of Chondrocyte Proliferation and Differentiation. *J. Bone Miner. Res.* **29**, 2653 (2014).](http://paperpile.com/b/v7jyQI/o3VOJ)

13. [Runx2 is required for hypertrophic chondrocyte mediated degradation of cartilage matrix during endochondral ossification. *Matrix Biology Plus* **12**, 100088 (2021).](http://paperpile.com/b/v7jyQI/GQT5j)

14. [Ono, K. *et al.* Dmrt2 promotes transition of endochondral bone formation by linking Sox9 and Runx2. *Communications Biology* **4**, 1–13 (2021).](http://paperpile.com/b/v7jyQI/LIPgj)

15. [Snelling, S. J., Hulley, P. A. & Loughlin, J. BMP5 activates multiple signaling pathways and promotes chondrogenic differentiation in the ATDC5 growth plate model. *Growth Factors* **28**, (2010).](http://paperpile.com/b/v7jyQI/Rj8wl)

16. [Bian, Q. *et al.* A single cell transcriptional atlas of early synovial joint development. *Development* **147**, (2020).](http://paperpile.com/b/v7jyQI/Z0kVB)

17. [Feng, C. *et al.* Lgr5 and Col22a1 Mark Progenitor Cells in the Lineage toward Juvenile Articular Chondrocytes. *Stem cell reports* **13**, (2019).](http://paperpile.com/b/v7jyQI/5cStj)

18. [Lee, W. *et al.* Synergy between Piezo1 and Piezo2 channels confers high-strain mechanosensitivity to articular cartilage. *Proc. Natl. Acad. Sci. U. S. A.* **111**, (2014).](http://paperpile.com/b/v7jyQI/GabeB)

19. [Feng, C. *et al.* Lgr5 and Col22a1 Mark Progenitor Cells in the Lineage toward Juvenile Articular Chondrocytes. *Stem cell reports* **13**, (2019).](http://paperpile.com/b/v7jyQI/guvCX)

20. [Beccari, L. *et al.* Dbx2 regulation in limbs suggests interTAD sharing of enhancers. *Dev. Dyn.* **250**, 1280 (2021).](http://paperpile.com/b/v7jyQI/SOtqW)

21. [Craft, A. M. *et al.* Specification of chondrocytes and cartilage tissues from embryonic stem cells. *Development* **140**, 2597–2610 (2013).](http://paperpile.com/b/v7jyQI/VSpGw)

22. [Pothiawala, A. *et al.* GDF5+ chondroprogenitors derived from human pluripotent stem cells preferentially form permanent chondrocytes. *Development* **149**, dev196220 (2022).](http://paperpile.com/b/v7jyQI/Y0xB4)

23. [Dowthwaite, G. P., Edwards, J. C. W. & Pitsillides, A. A. An Essential Role for the Interaction Between Hyaluronan and Hyaluronan Binding Proteins During Joint Development. *J. Histochem. Cytochem.* (1998) doi:](http://paperpile.com/b/v7jyQI/pWN22)[10.1177/002215549804600509](http://dx.doi.org/10.1177/002215549804600509)[.](http://paperpile.com/b/v7jyQI/pWN22)

24. [Palumbo-Zerr, K. *et al.* Composition of TWIST1 dimers regulates fibroblast activation and tissue fibrosis. *Ann. Rheum. Dis.* **76**, 244–251 (2017).](http://paperpile.com/b/v7jyQI/HaoYu)

25. [Twist2-driven chromatin remodeling governs the postnatal maturation of dermal fibroblasts. *Cell Rep.* **39**, 110821 (2022).](http://paperpile.com/b/v7jyQI/VkDQ1)

26. [Mascharak, S. *et al.* Preventing Engrailed-1 activation in fibroblasts yields wound regeneration without scarring. *Science* **372**, (2021).](http://paperpile.com/b/v7jyQI/xiwgC)

27. [Peters, H., Neubüser, A., Kratochwil, K. & Balling, R. Pax9-deficient mice lack pharyngeal pouch derivatives and teeth and exhibit craniofacial and limb abnormalities. *Genes Dev.* **12**, (1998).](http://paperpile.com/b/v7jyQI/1kkYY)

28. [Orestes-Cardoso, S. M. *et al.* Postnatal Msx1 expression pattern in craniofacial, axial, and appendicular skeleton of transgenic mice from the first week until the second year. *Dev. Dyn.* **221**, 1–13 (2001).](http://paperpile.com/b/v7jyQI/XbzYr)

29. [Zanotti, S. & Canalis, E. Activation of Nfatc2 in osteoblasts causes osteopenia. *J. Cell. Physiol.* **230**, (2015).](http://paperpile.com/b/v7jyQI/Rv7Gw)

30. [Ranger, A. M. *et al.* The Nuclear Factor of Activated T Cells (Nfat) Transcription Factor Nfatp (Nfatc2) Is a Repressor of Chondrogenesis. *J. Exp. Med.* **191**, 9–22 (2000).](http://paperpile.com/b/v7jyQI/N0imC)

31. [Zhu, K. *et al.* ATF4 promotes bone angiogenesis by increasing VEGF expression and release in the bone environment. *J. Bone Miner. Res.* **28**, 1870 (2013).](http://paperpile.com/b/v7jyQI/W3aKu)

32. [Wang, Y. *et al.* Ephrin-B2 controls VEGF-induced angiogenesis and lymphangiogenesis. *Nature* **465**, (2010).](http://paperpile.com/b/v7jyQI/UmZ9Q)

33. [Wang, F. *et al.* RACK1 regulates VEGF/Flt1-mediated cell migration via activation of a PI3K/Akt pathway. *J. Biol. Chem.* **286**, (2011).](http://paperpile.com/b/v7jyQI/3AOrr)

34. [Ruehle, M. A. *et al.* Mechanical Regulation of Microvascular Angiogenesis. *bioRxiv* 2020.01.14.906354 (2020) doi:](http://paperpile.com/b/v7jyQI/0ntiF)[10.1101/2020.01.14.906354](http://dx.doi.org/10.1101/2020.01.14.906354)[.](http://paperpile.com/b/v7jyQI/0ntiF)

35. [Matsushita, Y. *et al.* The fate of early perichondrial cells in developing bones. *Nat. Commun.* **13**, 1–17 (2022).](http://paperpile.com/b/v7jyQI/NqZMc)

36. [Ramasamy, S. K., Kusumbe, A. P., Wang, L. & Adams, R. H. Endothelial Notch activity promotes angiogenesis and osteogenesis in bone. *Nature* **507**, 376–380 (2014).](http://paperpile.com/b/v7jyQI/WSsDT)

37. [Baron, R. & Kneissel, M. WNT signaling in bone homeostasis and disease: from human mutations to treatments. *Nat. Med.* **19**, 179–192 (2013).](http://paperpile.com/b/v7jyQI/idtEf)

38. [Tomlinson, R. E. *et al.* NGF-TrkA Signaling by Sensory Nerves Coordinates the Vascularization and Ossification of Developing Endochondral Bone. *Cell Rep.* **16**, (2016).](http://paperpile.com/b/v7jyQI/IZN3X)

39. [Hu, B. *et al.* Sensory nerves regulate mesenchymal stromal cell lineage commitment by tuning sympathetic tones. *J. Clin. Invest.* **130**, (2020).](http://paperpile.com/b/v7jyQI/t1UWw)

40. [Taïhi, I., Nassif, A., Isaac, J., Fournier, B. P. & Ferré, F. Head to Knee: Cranial Neural Crest-Derived Cells as Promising Candidates for Human Cartilage Repair. *Stem Cells Int.* **2019**, 9310318 (2019).](http://paperpile.com/b/v7jyQI/bQo7L)

41. [Chilton, J. K. & Guthrie, S. Cranial expression of class 3 secreted semaphorins and their neuropilin receptors. *Dev. Dyn.* **228**, 726–733 (2003).](http://paperpile.com/b/v7jyQI/Hc4N7)

42. [Berndt, J. D. & Halloran, M. C. Semaphorin 3d promotes cell proliferation and neural crest cell development downstream of TCF in the zebrafish hindbrain. *Development* **133**, 3983–3992 (2006).](http://paperpile.com/b/v7jyQI/iWHQP)

43. [Bian, Q. *et al.* A single cell transcriptional atlas of early synovial joint development. *Development* **147**, (2020).](http://paperpile.com/b/v7jyQI/gRCcR)

44. [DeTomaso, D. & Yosef, N. Hotspot identifies informative gene modules across modalities of single-cell genomics. *Cell Syst.* **12**, 446–456.e9 (2021).](http://paperpile.com/b/v7jyQI/dBG7u)

45. [Wang, Z., Liu, B., Lin, K., Duan, C. & Wang, C. The presence and degradation of nerve fibers in articular cartilage of neonatal rats. *J. Orthop. Surg. Res.* **17**, 331 (2022).](http://paperpile.com/b/v7jyQI/zJ7VN)

46. [Yueh, Y. G., Gardner, D. P. & Kappen, C. Evidence for regulation of cartilage differentiation by the homeobox gene Hoxc-8. *Proc. Natl. Acad. Sci. U. S. A.* **95**, (1998).](http://paperpile.com/b/v7jyQI/yHuoj)

47. [Cartilage repair using bone morphogenetic protein 4 and muscle‐derived stem cells.](http://paperpile.com/b/v7jyQI/ghq0C) <http://dx.doi.org/10.1002/art.21632> [doi:](http://paperpile.com/b/v7jyQI/ghq0C)[10.1002/art.21632](http://dx.doi.org/10.1002/art.21632)[.](http://paperpile.com/b/v7jyQI/ghq0C)

48. [Yin, Z. *et al.* Atlas of Musculoskeletal Stem Cells with the Soft and Hard Tissue Differentiation Architecture. *Adv. Sci. Lett.* **7**, (2020).](http://paperpile.com/b/v7jyQI/06n9c)

49. [Grimaldi, A., Comai, G., Mella, S. & Tajbakhsh, S. Identification of bipotent progenitors that give rise to myogenic and connective tissues in mouse. *Elife* **11**, (2022).](http://paperpile.com/b/v7jyQI/bzhvm)

50. [Megas, S., Lorenzi, V. & Marioni, J. C. EmptyDropsMultiome discriminates real cells from background in single-cell multiomics assays. *Genome Biol.* **25**, 1–15 (2024).](http://paperpile.com/b/v7jyQI/9bEmr)

51. [Thibodeau, A. *et al.* AMULET: a novel read count-based method for effective multiplet detection from single nucleus ATAC-seq data. *Genome Biol.* **22**, 1–19 (2021).](http://paperpile.com/b/v7jyQI/nzBIZ)

52. [Heinonen, J. *et al.* Defects in chondrocyte maturation and secondary ossification in mouse knee joint epiphyses due to Snorc deficiency. *Osteoarthritis Cartilage* **25**, 1132–1142 (2017).](http://paperpile.com/b/v7jyQI/diAcs)

53. [Lee, H.-S. *et al.* Activation of Integrin-RACK1/PKCalpha signalling in human articular chondrocyte mechanotransduction. *Osteoarthritis Cartilage* **10**, 890–897 (2002).](http://paperpile.com/b/v7jyQI/wa1iQ)

54. [Zhang, F. *et al.* NFATc1 marks articular cartilage progenitors and negatively determines articular chondrocyte differentiation. *Elife* **12**, (2023).](http://paperpile.com/b/v7jyQI/8lmvC)

55. [Greenblatt, M. B. *et al.* NFATc1 and NFATc2 repress spontaneous osteoarthritis. *Proc. Natl. Acad. Sci. U. S. A.* **110**, 19914–19919 (2013).](http://paperpile.com/b/v7jyQI/y5Je9)

56. [Ren, R. *et al.* The role of Ca2+/Calcineurin/NFAT signalling pathway in osteoblastogenesis. *Cell Prolif.* **54**, e13122 (2021).](http://paperpile.com/b/v7jyQI/Bsh7b)

57. [Genetics of developmental dysplasia of the hip. *Eur. J. Med. Genet.* **63**, 103990 (2020).](http://paperpile.com/b/v7jyQI/43Ohk)

58. [Zhang, X. *et al.* Lipid peroxidation in osteoarthritis: focusing on 4-hydroxynonenal, malondialdehyde, and ferroptosis. *Cell Death Discovery* **9**, 1–13 (2023).](http://paperpile.com/b/v7jyQI/CaAee)

59. [Mathijssen, I. M. *et al.* Tracing craniosynostosis to its developmental stage through bone center displacement. *J. Craniofac. Genet. Dev. Biol.* **19**, (1999).](http://paperpile.com/b/v7jyQI/VpIBL)

60. [Lajeunie, E., Le Merrer, M., Bonaïti-Pellie, C., Marchac, D. & Renier, D. Genetic study of nonsyndromic coronal craniosynostosis. *Am. J. Med. Genet.* **55**, (1995).](http://paperpile.com/b/v7jyQI/KnGKp)

61. [Wilkie, A. O. *et al.* Prevalence and complications of single-gene and chromosomal disorders in craniosynostosis. *Pediatrics* **126**, (2010).](http://paperpile.com/b/v7jyQI/g96Mp)

62. [Sharma, V. P. *et al.* Mutations in TCF12, encoding a basic helix-loop-helix partner of TWIST1, are a frequent cause of coronal craniosynostosis. *Nat. Genet.* **45**, 304–307 (2013).](http://paperpile.com/b/v7jyQI/fl0Yc)

63. [Yoon, W. J. *et al.* The Boston-type craniosynostosis mutation MSX2 (P148H) results in enhanced susceptibility of MSX2 to ubiquitin-dependent degradation. *J. Biol. Chem.* **283**, (2008).](http://paperpile.com/b/v7jyQI/2zHrk)

64. [Bruderer, M., Richards, R. G., Alini, M. & Stoddart, M. J. Role and regulation of RUNX2 in osteogenesis. *Eur. Cell. Mater.* **28**, 269–286 (2014).](http://paperpile.com/b/v7jyQI/ZHf6v)

65. [Ren, R. *et al.* The role of Ca2+/Calcineurin/NFAT signalling pathway in osteoblastogenesis. *Cell Prolif.* **54**, e13122 (2021).](http://paperpile.com/b/v7jyQI/2ztF4)

66. [van Lierop, A. H. *et al.* Van Buchem disease: clinical, biochemical, and densitometric features of patients and disease carriers. *J. Bone Miner. Res.* **28**, 848–854 (2013).](http://paperpile.com/b/v7jyQI/HrhJ9)

67. [Collette, N. M. *et al.* Targeted deletion of Sost distal enhancer increases bone formation and bone mass. *Proc. Natl. Acad. Sci. U. S. A.* **109**, 14092–14097 (2012).](http://paperpile.com/b/v7jyQI/bJSWS)

68. [James, A. W., Levi, B., Xu, Y., Carre, A. L. & Longaker, M. T. Retinoic acid enhances osteogenesis in cranial suture-derived mesenchymal cells: potential mechanisms of retinoid-induced craniosynostosis. *Plast. Reconstr. Surg.* **125**, 1352–1361 (2010).](http://paperpile.com/b/v7jyQI/pwst9)

69. [Clouthier, D. E. *et al.* Cranial and cardiac neural crest defects in endothelin-A receptor-deficient mice. *Development* **125**, 813–824 (1998).](http://paperpile.com/b/v7jyQI/Zr6NR)

70. [Spence, S., Anderson, C., Cukierski, M. & Patrick, D. Teratogenic effects of the endothelin receptor antagonist L-753,037 in the rat. *Reprod. Toxicol.* **13**, 15–29 (1999).](http://paperpile.com/b/v7jyQI/cmUBp)

71. [Zhou, X. *et al.* Chondrocytes Transdifferentiate into Osteoblasts in Endochondral Bone during Development, Postnatal Growth and Fracture Healing in Mice. *PLoS Genet.* **10**, e1004820 (2014).](http://paperpile.com/b/v7jyQI/Q0qqS)
