# Supplementary material for: A multi-omic atlas of human embryonic skeletal development
Source: Nature. 2024 Nov 20;635(8039):657–67. doi: 10.1038/s41586-024-08189-z (PMC11578895; doi:10.1038/s41586-024-08189-z)
Supplement: Supplementary file 2 — Reporting Summary [file 41586_2024_8189_MOESM2_ESM.pdf]

nature portfolio

Corresponding author(s): Sarah A. Teichmann

Last updated by author(s): Oct 8, 2024

Reporting Summary

Nature Portfolio wishes to improve the reproducibility of the work that we publish. This form provides structure for consistency and transparency in reporting. For further information on Nature Portfolio policies, see our Editorial Policies and the Editorial Policy Checklist.

Statistics

For all statistical analyses, confirm that the following items are present in the figure legend, table legend, main text, or Methods section.

n/a	Confirmed	
☐	☒	The exact sample size (*n*) for each experimental group/condition, given as a discrete number and unit of measurement
☐	☒	A statement on whether measurements were taken from distinct samples or whether the same sample was measured repeatedly
☐	☒	The statistical test(s) used AND whether they are one- or two-sided<br>*Only common tests should be described solely by name; describe more complex techniques in the Methods section.*
☐	☒	A description of all covariates tested
☐	☒	A description of any assumptions or corrections, such as tests of normality and adjustment for multiple comparisons
☐	☒	A full description of the statistical parameters including central tendency (e.g. means) or other basic estimates (e.g. regression coefficient) AND variation (e.g. standard deviation) or associated estimates of uncertainty (e.g. confidence intervals)
☐	☒	For null hypothesis testing, the test statistic (e.g. *F*, *t*, *r*) with confidence intervals, effect sizes, degrees of freedom and *P* value noted<br>*Give P values as exact values whenever suitable.*
☒	☐	For Bayesian analysis, information on the choice of priors and Markov chain Monte Carlo settings
☒	☐	For hierarchical and complex designs, identification of the appropriate level for tests and full reporting of outcomes
☐	☒	Estimates of effect sizes (e.g. Cohen's *d*, Pearson's *r*), indicating how they were calculated

Our web collection on statistics for biologists contains articles on many of the points above.

Software and code

Policy information about availability of computer code

Data collection	The following software was used to process the raw data:<br>CellRanger-ARC v2.0.0<br>Souporcell v2.0<br>SoupX v1.6.0<br>Scrublet v0.2.3<br>AMULET v1.1.0<br>SpaceRanger v1.1.0<br>LoupeBrowser v7.0<br>Imspector Pro 7.5.3 acquisition software<br>ImarisFileConverter 10.1<br>EmptyDropMultiome 1.0.0<br>Velocyto 0.17.17
Data analysis	The following software was used for data analysis:<br>Scanpy v1.9.6<br>ArchR v1.0.2<br>Muon v0.1.2<br>Scenic+ v1.0.0<br>CellOracle v0.12.0<br>scFates v1.0.3<br>CellRank v2.0.2

Monocle3 v1.0.0
Scvelo v0.2.3
CellHint v1.0.0
Milopy v0.1.1
Cell2location v0.1.4
CellPhoneDB v4.0.0
NicheNet v1.1.1
R 4.0.4
Python 1.7.1
FlowJo v9
Macs2 2.2.7.1
MultiVI (scvi: 0.6.8)
scVI 0.9.1
Celltypist 1.6.3
Palantir 1.3.3
Bbknn 1.5.1
PyCisTopic 1.0.2
PyCisTarget 1.0.2
Arboreto 0.1.6
ComplexHeatmap 2.6.2
Seriation 1.3.0
Starsolo 2.7.10a

And the following software was used for image processing:
MACS® iQ View Software v1.0
Adobe Photoshop 25.2
Microaligner 1.0.0
CellPose 3.0
PoSTcode v1.0
STRtree 2.0.6

Drug2cell code is available at https://github.com/Teichlab/drug2cell.
TissueTag code is available at https://github.com/Teichlab/TissueTag.
EmptyDropsMultiome code is available at https://github.com/MarioniLab/EmptyDropsMultiome.
fGWAS code is available at https://github.com/cellgeni/nf-fgwas.
ISS-Patcher is available at https://github.com/Teichlab/iss_patcher.
SNP2CELL is available at https://github.com/Teichlab/snp2cell.

For manuscripts utilizing custom algorithms or software that are central to the research but not yet described in published literature, software must be made available to editors and reviewers. We strongly encourage code deposition in a community repository (e.g. GitHub). See the Nature Portfolio guidelines for submitting code & software for further information.

Data

Policy information about availability of data

All manuscripts must include a data availability statement. This statement should provide the following information, where applicable:

- Accession codes, unique identifiers, or web links for publicly available datasets
- A description of any restrictions on data availability
- For clinical datasets or third party data, please ensure that the statement adheres to our policy

The annotated droplet and spatial data objects for this study can be accessed here: http://developmental.cellatlas.io/skeleton-development.

High-throughput raw sequencing data in this study is available from ArrayExpress (www.ebi.ac.uk/arrayexpress) with the following accession number: E-MTAB-14385.
Human reference genome version 2020-A (GRCh38-2020-A-2.0.0) was downloaded using cellranger-arc v2.0.0.

Data analysed from Zhang et al 2023, are accessible from the following portal https://developmental.cellatlas.io/embryonic-limb .

Full GWAS summary statistics for hip-, knee- and overall osteoarthritis, as well as total knee- and hip replacement from the Genetics of Osteoarthritis (GO) Consortium were obtained from the musculoskeletal knowledge portal (https://msk.hugeamp.org/downloads.html).

Research involving human participants, their data, or biological material

Policy information about studies with human participants or human data. See also policy information about sex, gender (identity/presentation), and sexual orientation and race, ethnicity and racism.

Reporting on sex and gender	Due to the early embryonic stages of the tissue samples; samples received within the first trimester from our tissue resource did NOT contain sex information. Findings apply to all sexes and sex and gender were not considered in research design. The studied processes during early embryonic and foetal development are relevant for all sexes.
Reporting on race, ethnicity, or	Donors were not distinguished based on race, ethnicity or other socially relevant groupings.

other socially relevant groupings	
Population characteristics	Population characteristics of the donors were not considered, however, donations were selected to span 5-11 post conception weeks and only healthy tissues were used. Embryonic age determined through estimated physical parameters of the embryo, e.g. Crown-rump Length were utilised to determine the approximate age of the samples and were recorded for analyses.
Recruitment	Embryonic samples were donated voluntarily by women who underwent termination of pregnancy from the Cambridge Centre for Brain Repair, Cambridge, UK
Ethics oversight	Wellcome Sanger Institute, UK<br><br>AND<br><br>Research and Development Department<br>Cambridge University Hospitals NHS Foundation Trust Cambridge Biomedical Campus<br><br>The human embryonic and fetal material were provided under the following references:<br>Title: Invitro studies of postmortem human foetal neural tissue<br>REC reference: 96/085<br>IRAS project ID: 95602<br><br>AND<br><br>For whole-mount immunostaining, samples used in this study were obtained from terminations of pregnancy with written and informed consent from all sample donors. Samples were provided by INSERM's HuDeCA Biobank and utilised in compliance with French regulations. Authorization to use these tissues was granted by the French agency for biomedical research (Agence de la Biomédecine, Saint-Denis La Plaine, France; N° PFS19-012) and the INSERM Ethics Committee (IRB00003888).

Note that full information on the approval of the study protocol must also be provided in the manuscript.

Field-specific reporting

Please select the one below that is the best fit for your research. If you are not sure, read the appropriate sections before making your selection.

☒ Life sciences ☐ Behavioural & social sciences ☐ Ecological, evolutionary & environmental sciences

For a reference copy of the document with all sections, see nature.com/documents/nr-reporting-summary-flat.pdf

Life sciences study design

All studies must disclose on these points even when the disclosure is negative.

Sample size	No sample size calculation was performed. Sample acquisition depended on the availability of suitable tissue donations, resource availability to study samples and the study period. Available donations were selected to span the range between 5 to 11 post conception weeks in approximately equal time steps with multiple samples per time point. In addition, samples were taken at multiple selected time points for spatial transcriptomics analyses. Analyses in this manuscript are based on an integration of samples across time points, locations and modalities and sampling density across time points was chosen to be comparable to similar studies in the field.
Data exclusions	No samples were excluded from the study. Collected data was subjected to quality control after profiling. Cells with low quality according to established procedures and RNA/ATAC based metrics were excluded from the analysis. Droplets were filtered for >200 genes, and <5% mitochondrial and ribosomal reads. Moreover, cells with TSSenrichment score<7 and nFrags< 1000 were removed. Doublet removal was performed using Scrublet for RNA and ArchR as well as AMULET for ATAC.
Replication	Sample donors and number of replicates are stated in supplementary table 1. All attempts at replication were successful. Study results were checked for consistency with previous findings in the literature. For annotated cell states, we observed they were replicated across multiple donors. For computational analyses, parameters were explored extensively to ensure reproducibility of findings. For imaging data displayed, the number of replicates are stated in each figure legend.
Randomization	Randomization was not relevant to this study. Sample acquisition depended upon availability of tissue donations. Donor sample developmental stages were selected to span the available first-trimester stages (5-11PCW), ensuring approximate coverage of each post-conception week for the droplet data.
Blinding	Blinding was not required for this work as we did not allocate treatment and control groups, and did not administer treatment or test conditions.

Reporting for specific materials, systems and methods

We require information from authors about some types of materials, experimental systems and methods used in many studies. Here, indicate whether each material, system or method listed is relevant to your study. If you are not sure if a list item applies to your research, read the appropriate section before selecting a response.

Materials & experimental systems

n/a	Involved in the study	
☐	☒	Antibodies
☒	☐	Eukaryotic cell lines
☒	☐	Palaeontology and archaeology
☒	☐	Animals and other organisms
☒	☐	Clinical data
☒	☐	Dual use research of concern
☒	☐	Plants

Methods

n/a	Involved in the study	
☒	☐	ChIP-seq
☐	☒	Flow cytometry
☒	☐	MRI-based neuroimaging

Antibodies

Antibodies used

SP7 antibody (supplier name: abcam; Catalog: ab209484; Clone name: EPR21034; dilution: 1:500)
COL2A1 antibody (supplier name: abcam, Catalog: ab185430, Clone name: 2B1.5; dilution: 1:500)
FGFR3 antibody (supplier name: Thermo Fisher Scientific; Catalog: MA5-38521; Clone name: 2H10B4 ; Lot number: YL4148366; dilution: 1:50)
TACR3 antibody (supplier name: Thermo Fisher Scientific; Catalog: BS-0166R; Clone name: bs-0166R-A488; Lot number: BB03107336; dilution: 1:50)

Validation

literature on validation experiments are curated and accessible on the corresponding manufacturer website:
FGFR3: https://www.thermofisher.com/antibody/product/FGFR3-Antibody-clone-2H10B4-Monoclonal/MA5-38521
TACR3: https://www.thermofisher.com/antibody/product/TAC3R-Antibody-clone-2H10B4-Monoclonal/BS-0166R
SP7: https://www.abcam.com/en-gb/products/primary-antibodies/sp7-osterix-antibody-epr21034-ab209484
COL2A1: https://doc.abcam.com/datasheets/inactive/ab185430/en-us/collagen-ii-antibody-2b15-ab185430.pdf

Plants

Seed stocks

Report on the source of all seed stocks or other plant material used. If applicable, state the seed stock centre and catalogue number. If plant specimens were collected from the field, describe the collection location, date and sampling procedures.

Novel plant genotypes

Describe the methods by which all novel plant genotypes were produced. This includes those generated by transgenic approaches, gene editing, chemical/radiation-based mutagenesis and hybridization. For transgenic lines, describe the transformation method, the number of independent lines analyzed and the generation upon which experiments were performed. For gene-edited lines, describe the editor used, the endogenous sequence targeted for editing, the targeting guide RNA sequence (if applicable) and how the editor was applied.

Authentication

Describe any authentication procedures for each seed stock used or novel genotype generated. Describe any experiments used to assess the effect of a mutation and, where applicable, how potential secondary effects (e.g. second site T-DNA insertions, mosiacism, off-target gene editing) were examined.

Flow Cytometry

Plots

Confirm that:

☒ The axis labels state the marker and fluorochrome used (e.g. CD4-FITC).

☒ The axis scales are clearly visible. Include numbers along axes only for bottom left plot of group (a 'group' is an analysis of identical markers).

☒ All plots are contour plots with outliers or pseudocolor plots.

☒ A numerical value for number of cells or percentage (with statistics) is provided.

Methodology

Sample preparation

Prior to cell extraction, the sample tissues (~9 PCW shoulder joints) were dissected to obtain bone samples, and soft tissues were micro-dissected away. The resultant cell suspension was stained with DAPI (Invitrogen) for live-viability, FGFR3 antibody (MA5-38521, Thermo Fisher Scientific) and TAC3R antibody (BS-0166R, Thermo Fisher Scientific), and secondary

	antibodies.
Instrument	BigFoot Spectral Cell Sorter (Thermo Fisher Scientific)
Software	Thermo Fisher Scientific BigFoot proprietary software and flow Jo
Cell population abundance	The characterization of sorted population percentages were determined using flowjo, the sorted cells were not further subject to experimentation
Gating strategy	DAPI positive singlet cells were gated for DAPI staining by FACS. Sequential gating for FGFR3 and TAC3R was then conducted to identify double-positive cells. Positive controls for FGFR3 and TAC3R was conducted using human PBMCs and unstained cells were used as negative controls.

☒ Tick this box to confirm that a figure exemplifying the gating strategy is provided in the Supplementary Information.
