# Supplementary material for: A multi-omic atlas of human embryonic skeletal development
Source: Nature. 2024 Nov 20;635(8039):657–67. doi: 10.1038/s41586-024-08189-z (PMC11578895; doi:10.1038/s41586-024-08189-z)
Supplement: Supplementary file 3 — Supplementary Figures [file 41586_2024_8189_MOESM3_ESM.zip › 2023-10-17784B-s3/SuppFigures.docx]

**Supplementary Figure 1. Cell extraction bias analysis.**

**a.** Dotplot of cluster proportion analysis for clusters across regions and developmental stage, showing region-specific enrichment in expected patterns. **b.** Cell type proportion across ISS and snRNAseq cell state proportions and ISS cell state proportions. **c.** Correlation and difference testing scores between snRNAseq cell state proportions and ISS cell state proportions. **d.** Correlation and difference testing scores between snRNAseq cell state proportions and Visium cell state proportions. PCC and SSIM was used to assess similarity, whereas JSD and RMSE was used to test dissimilarity. PCC: Pearson correlation coefficient; SSIM: structural similarity index measure; JSD: Jensen-Shannon Divergence; RMSE: Root mean square error

**Supplementary Figure 2. Limb joint cavitation analysis**

**a.** Cavitation score computed from literature and GO-database across sample age (PCW) for each knee, hip and shoulder joints, showing peak enrichment before 8 PCW for knee and shoulder, and around approximately 9-10 PCW for hip joint. **b.** enrichment of CD44 (hyaluronate receptor) expression in the knee, hip and shoulder joints across sample age (PCW) showing comparable trend with panel a. **c.** GO terms correlation with cavitation score for each knee, hip and shoulder, showing monocyte aggregation as top-ranked pathway across the three joints.

**Supplementary Figure 3. Regulation of RUNX2**

**a.** eGRN showing the regulation of *RUNX2* predicted with SCENIC+. Various TFs (circles) are predicted to regulate *RUNX2* (square) via binding to regulatory elements (diamonds). The colour and width of arrows from regions to *RUNX2* show importance scores from GRNBoost2. Links from two TFs appearing in Fig. 2h and 6d are highlighted in red. Various osteogenic TFs, including *SP7*, are predicted to regulate *RUNX2*.

**Supplementary Figure 4. Reprocessing of Data from Zhang et al identifies *PAX7*+ chondrocytes that were not analysed**

**a.** workflow for reprocessing raw data from Zhang et al, including stringent quality control of droplets (scAutoQC; <http://github.com/Teichlab/sctk>). **b.** UMAP embedding of scaled gene expression of *PAX7*, *ACAN* and droplets removed by Zhang et al, showing a cluster that co-express *ACAN* and *PAX7*. **c.** Frequency histogram showing distribution of cells removed by Zhang et al across sample stage and regions sampled.

**Supplementary Figure 5. *PAX7^+^* Chon gene expression and regulation**

**a.** RNA UMAP embeddings (upper panel) of representative chondrogenic (*ACAN*, *COL2A1*) and myogenic (*PAX7*, *MEGF10*) genes for *PAX7*^+^chondrocytes. Genome browser tracks (lower panel) from scATAC-seq analyses displaying the sum signal within the indicated gene loci. OtherChon represents the whole chondro-lineages except *PAX7*^+^Chon. **b.** Enhancer-GRN showing enriched TFs in *PAX7*^+^Chon, inferred using SCENIC+. Circles and diamonds represent genes and regions with TF-binding sites, respectively. Colour indicates whether the TF of *PAX7*^+^Chon is also differentially expressed in chondro-lineages or *PAX7*^+^myocytes. TF-region links are coloured by TF. **c.** RNA-ISH for cells that co-expressed myogenic (*PAX7*, red) and chondrogenic markers (*ACAN*, green) in the shoulder at 7.3PCW. Blue marks the nucleus. Scale bar is shown in white.

**Supplementary Figure 6. Features of *PAX7^+^* Chon**

**a**. Boxplot showing the gene scores of cartilage development. Boxplot center line, median; boxes, first and third quartiles of the distribution; whiskers, highest and lowest data points within 1.5 × interquartile ratio (IQR). OtherChon represents chondro-lineages excluding *PAX7*^+^ Chon. The sample size of each category is listed as below, OtherChon: n=54,280 cells, *PAX7*^+^ Chon: n=2,702 cells, and *PAX7*^+^ Myo: n=32,027 cells. **b**. Boxplot showing the gene scores of muscle development. Boxplot center line, median; boxes, first and third quartiles of the distribution; whiskers, highest and lowest data points within 1.5 ×  IQR. The sample size of each category is listed as below, OtherChon: n=54,280 cells, *PAX7*^+^ Chon: n=2,702 cells, and *PAX7*^+^ Myo: n=32,027 cells. **c**. Barplot showing the percentage of samples from different donors in each chondrocyte subtype. Different colours represent different samples from donors. **d**. Barplot showing the percentage of cells that passed or did not pass cell calling by EmptyDropsMultiome in each cell type category. Other cells represent all captured cells in this atlas apart from *PAX7^+^*Chon. **e**. Scatter plot showing the distribution of all the droplets that were acquired after cell calling by CellRanger-ARC software. X-axis is the log q values (FDR using Benjamini-Hochberg adjustment) that were calculated by AMULET using ATAC data. Y-axis is the adjusted p-value (using Benjamini-Hochberg adjustment) from the adapted scrublet workflow using RNA data. Lines represent threshold values that we applied when filtering potential doublets. Red dots represent all the cells from *PAX7^+^*Chon, grey dots represent all the other droplets that passed all QC and light grey represents failed droplets. **f**. Deviation of genes of *PAX7*^+^ Chon from the linear mix for expression profiles of ChondroPro1, *PAX7*^+^ Myoprogenitor1, and SkM.Mesen[^1^](https://paperpile.com/c/v7jyQI/CVJj). **g**. Barplot showing enriched GO terms of top50 genes. All the statistical tests were performed using a two-sided hypothesis.

**Supplementary Figure 7. Experimental exploration of *PAX7^+^* Chon**

**a**. RNA-ISH for cells that co-expressed myogenic markers (*PAX7*, yellow) and chondrogenic markers (*SOX9*, red; *TRPV4*, green; *ACAN*, pink/purple ) in the shoulder at 10.3 PCW, knee at 10.3 PCW and shoulder at 6 PCW. Blue is DAPI. Scale bars are shown in white. Images are representative of sections from two donors. **b**. Dotplot showing gene expression of *FGFR3* and *TACR3*, which were co-expressed specifically by *PAX7^+^*Chon, and not co-expressed in myo-lineage or other cell types. Dot size represents the fraction of cells in each cluster and dot colour represents scaled mean expression. **c**. Flow cytometry cell sorting of dissociated bone-chip whole-cells from 9 PCW shoulder tissue samples. Forward scatter profile was applied to gate for singlets, followed by DAPI signal for live-dead. Subsequently, doublet-positive cells for *TAC3R* and *FGFR3* were identified as *PAX7*^+^Chon, whereas *TAC3R* single-positives represent myogenic cells and *FGFR3* single-positives represent chondrocytes. **d**. UMAP embedding of myogenic compartment. Colours represent the cell type annotation.

**Supplementary Figure 8. RNA-ISH expression quantification**
Boxplots of RNA-ISH expression from regions within Supplementary Fig. 4c. SOX9 expression and ACAN expression, which are markers of chondrocytes, are significantly greater in subchondral regions where chondrocytes are observed. TTN, and PAX7 expression, markers of myocytes, are greater in positive control regions compared to the other regions. PAX7 is greater in Subchondral chondrocyte regions than in negative control regions. Each box extends from the first to the third quartile (Q3) of the data, with a center line at the median. The whiskers extend from the box to the farthest data point lying within 1.5x the inter-quartile range (IQR) from the box.
