# Supplementary figures and images for: A multi-omic atlas of human embryonic skeletal development
Source: Nature. 2024 Nov 20;635(8039):657–67. doi: 10.1038/s41586-024-08189-z (PMC11578895; doi:10.1038/s41586-024-08189-z)

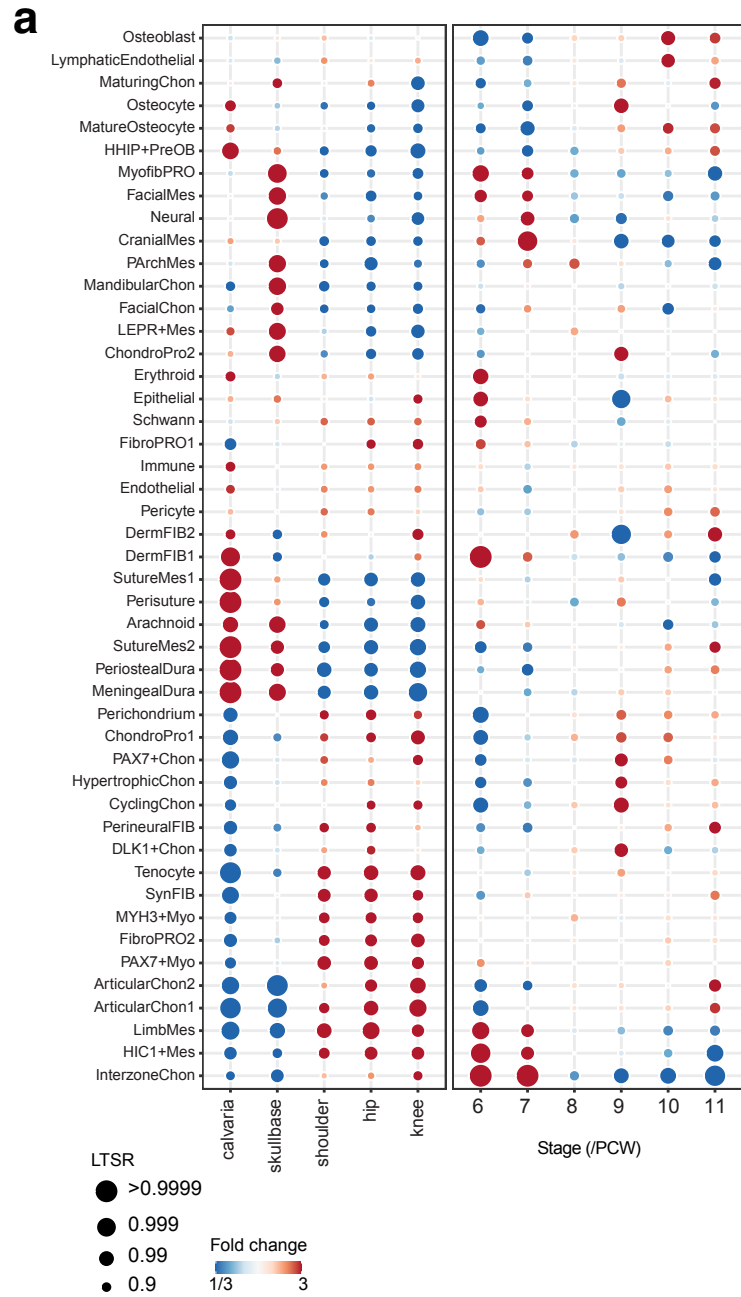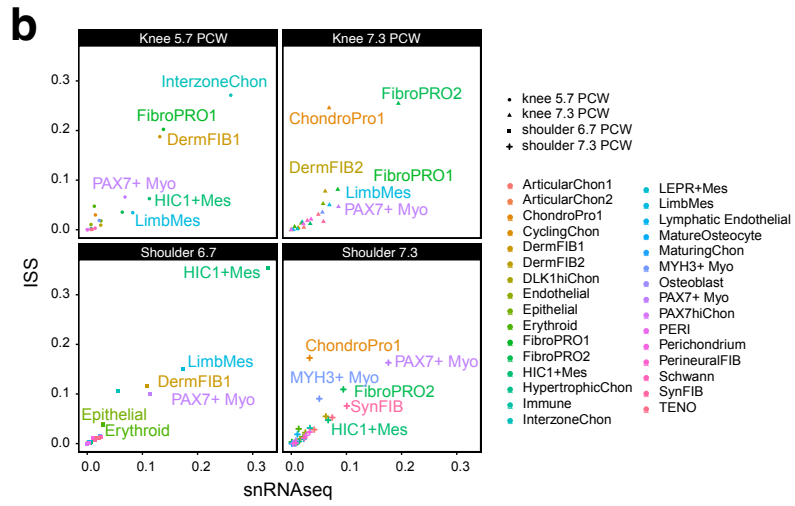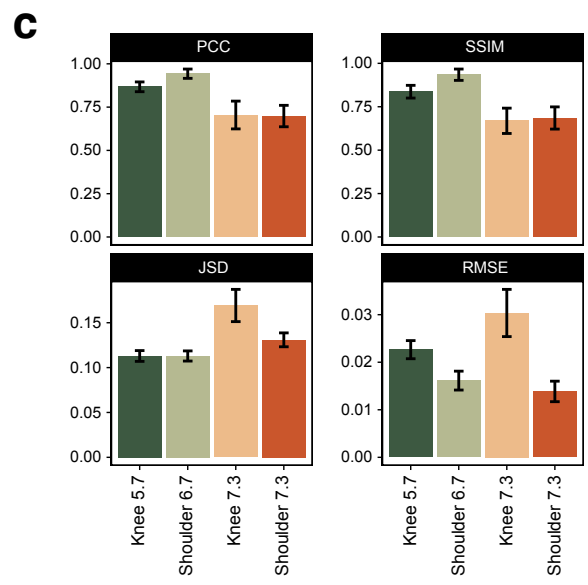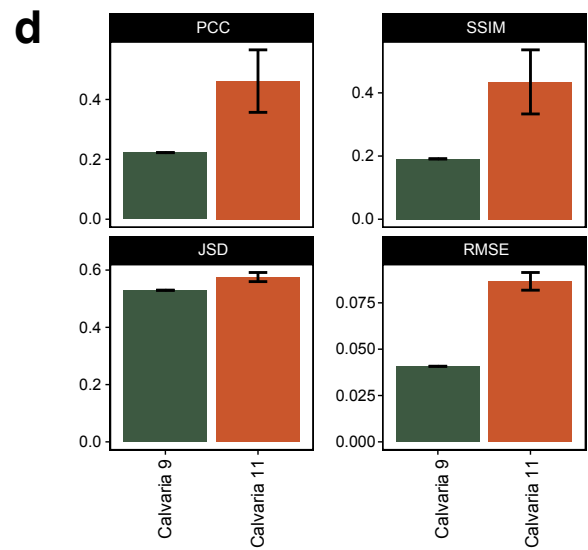

Supplement: Supplementary file 3 — Supplementary Figures [file 41586_2024_8189_MOESM3_ESM.zip › 2023-10-17784B-s3/2023-10-17784B-Supplementary Data Figure 1.pdf]

**a**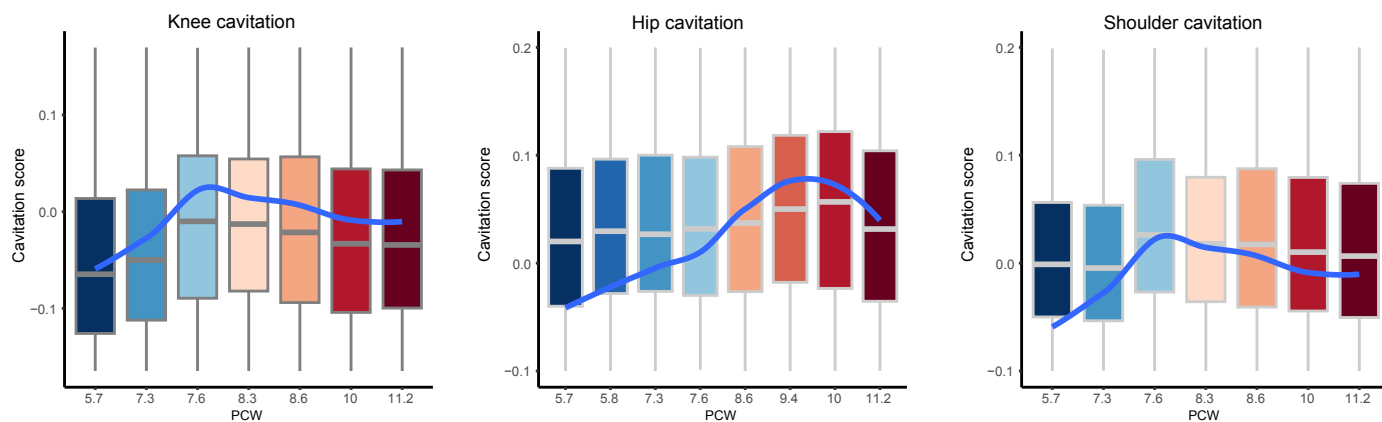**b**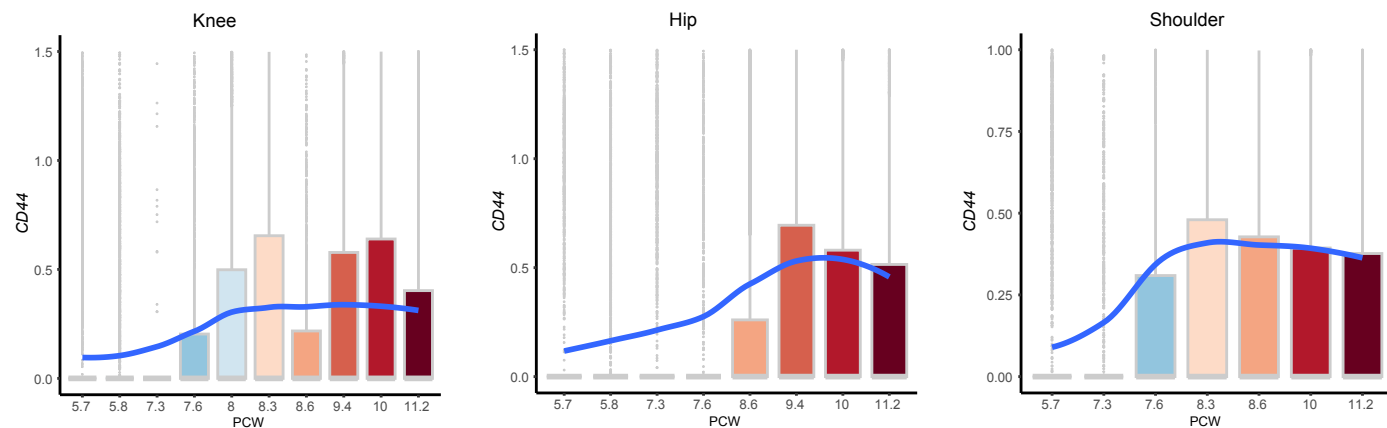**c**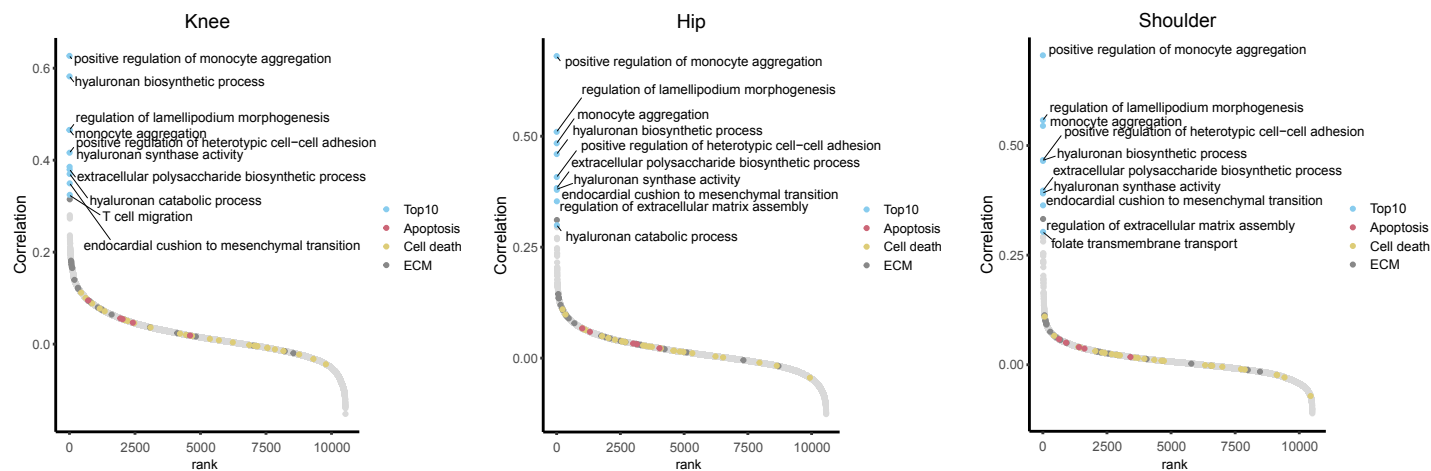

Supplement: Supplementary file 3 — Supplementary Figures [file 41586_2024_8189_MOESM3_ESM.zip › 2023-10-17784B-s3/2023-10-17784B-Supplementary Data Figure 2.pdf]

**a**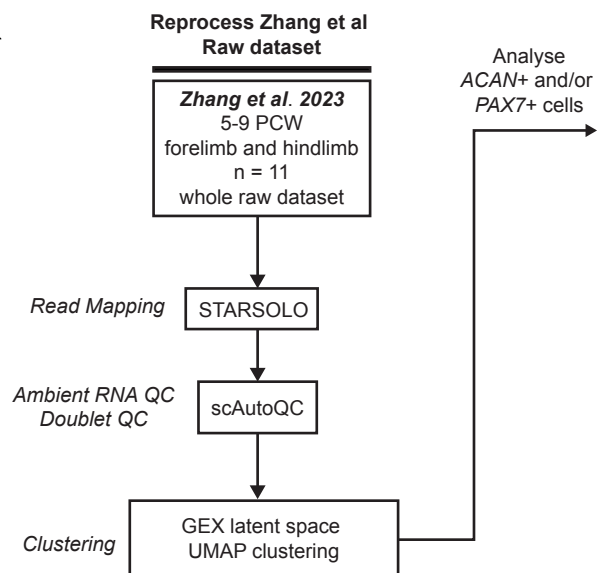**b**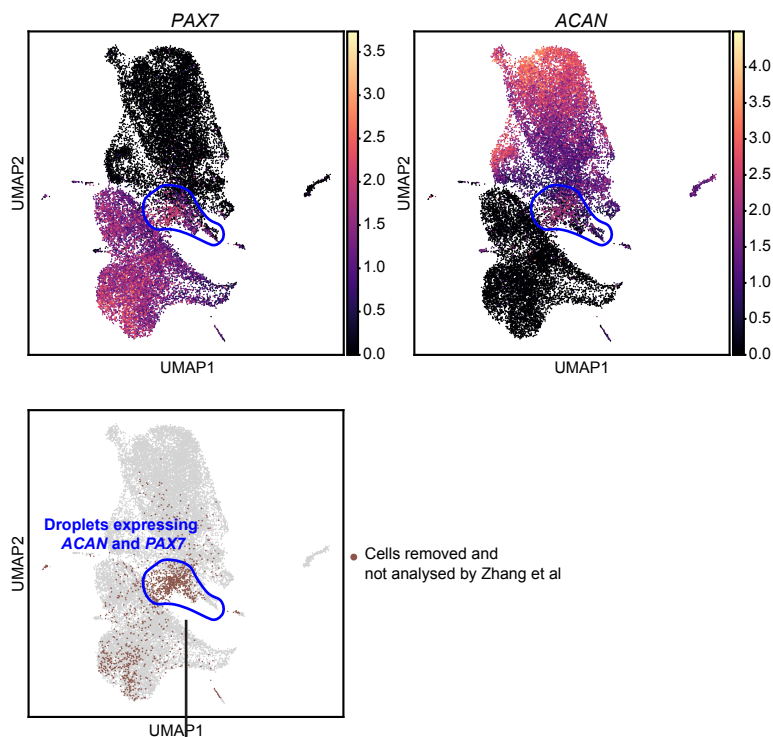**c**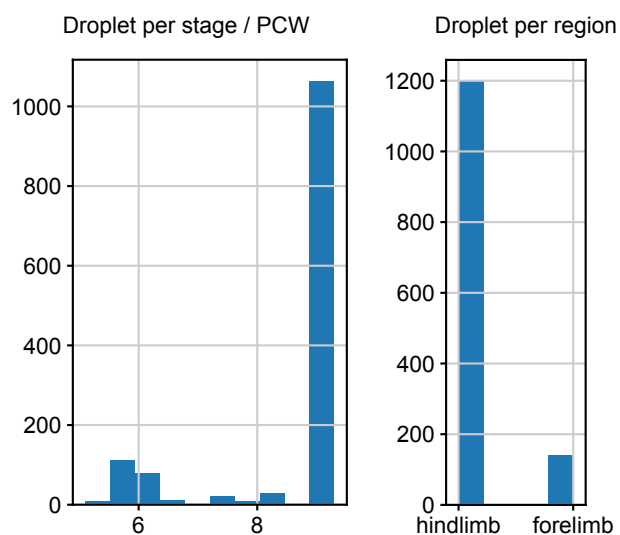

Supplement: Supplementary file 3 — Supplementary Figures [file 41586_2024_8189_MOESM3_ESM.zip › 2023-10-17784B-s3/2023-10-17784B-Supplementary Data Figure 3.pdf]

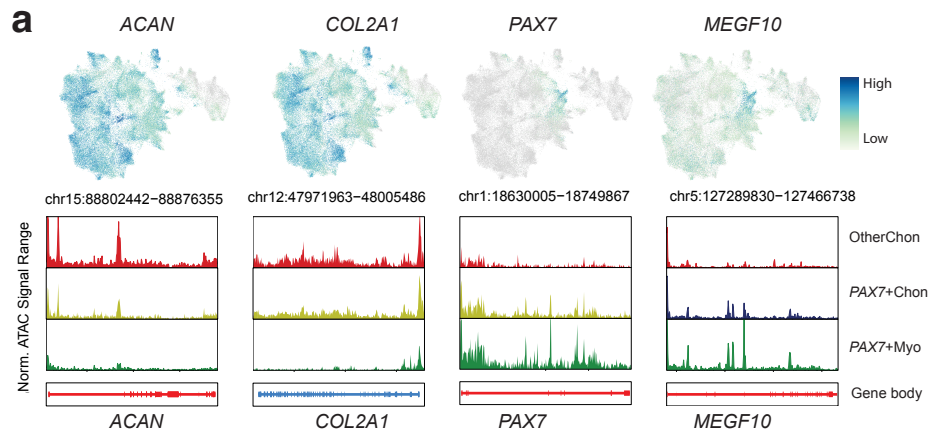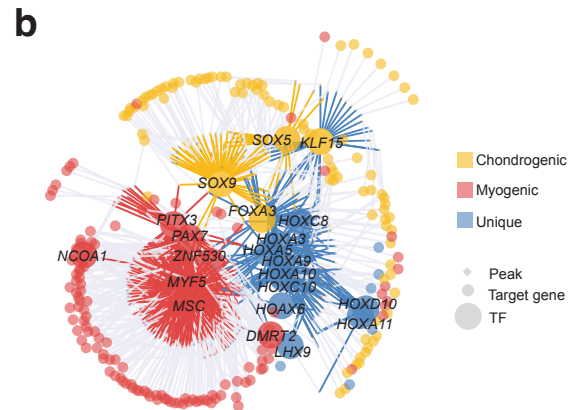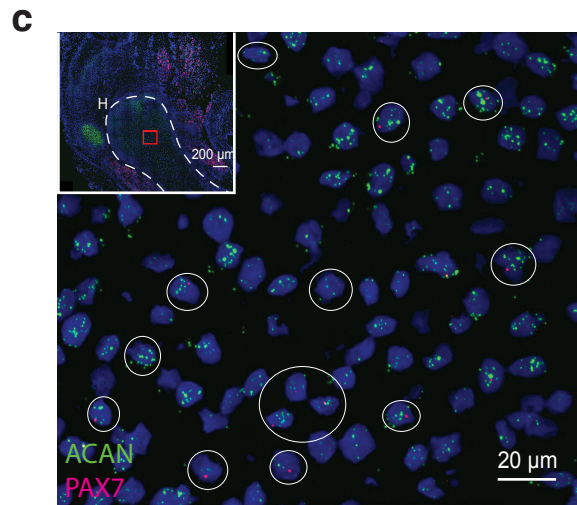

Supplement: Supplementary file 3 — Supplementary Figures [file 41586_2024_8189_MOESM3_ESM.zip › 2023-10-17784B-s3/2023-10-17784B-Supplementary Data Figure 4.pdf]

a

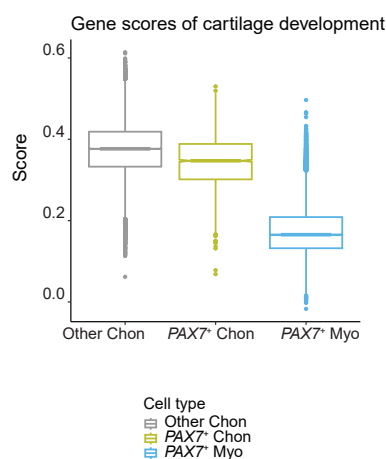

b

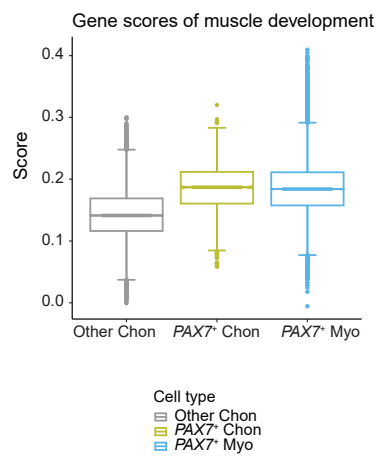

c

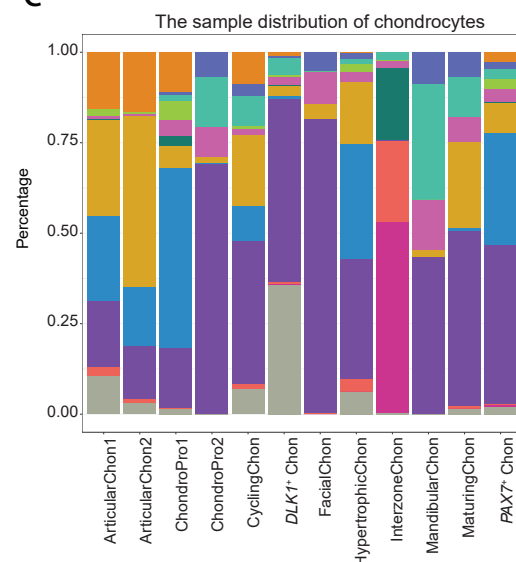

d

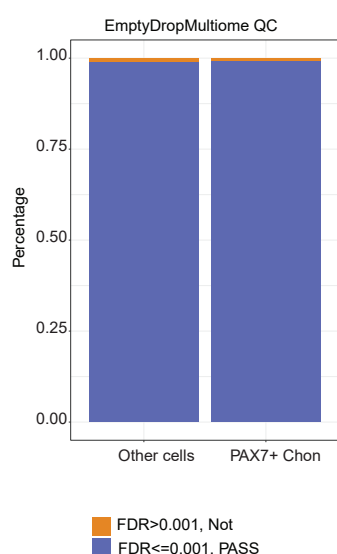

e

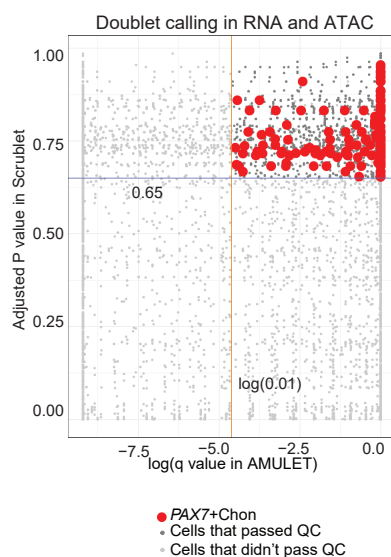

f

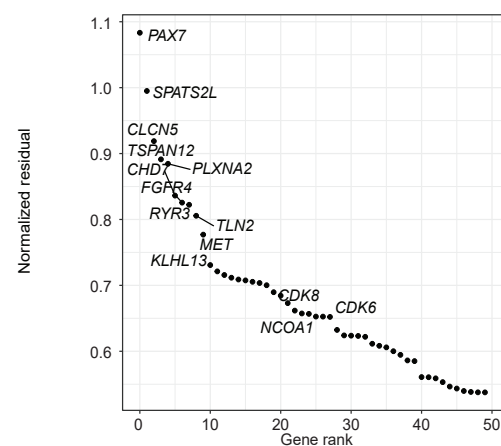

g

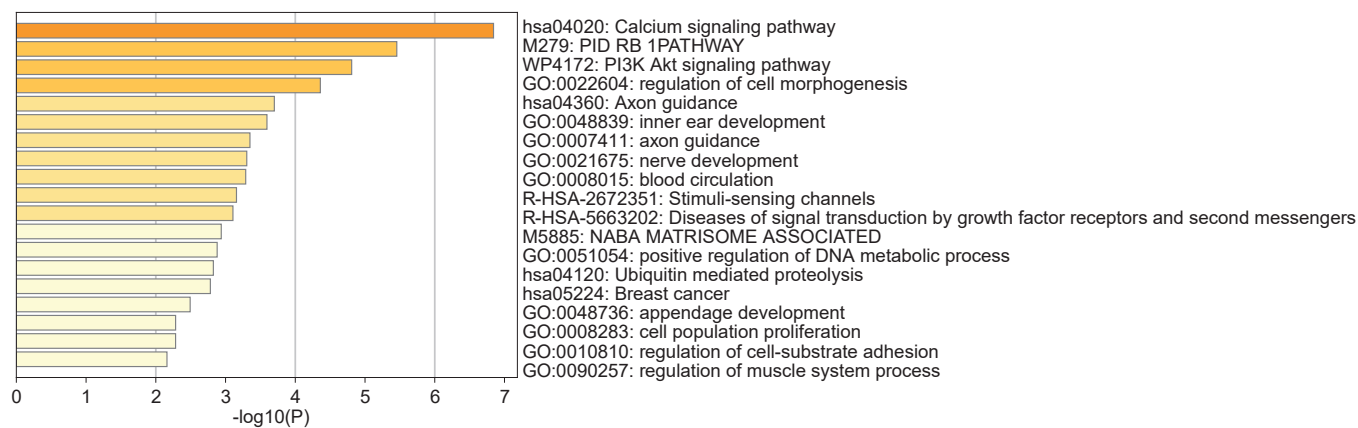

Supplement: Supplementary file 3 — Supplementary Figures [file 41586_2024_8189_MOESM3_ESM.zip › 2023-10-17784B-s3/2023-10-17784B-Supplementary Data Figure 5.pdf]

**a**

10.3 PCW Shoulder

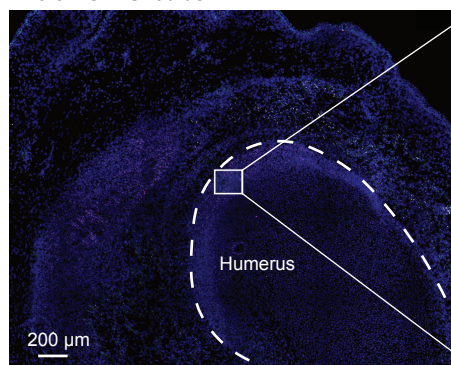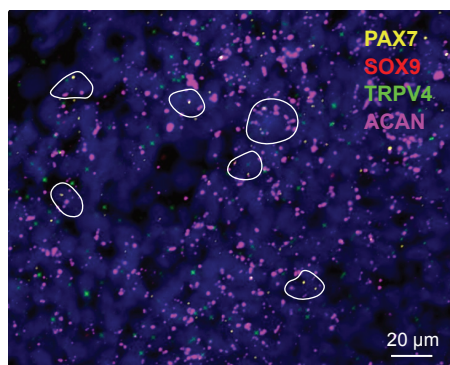

10.3 PCW Knee

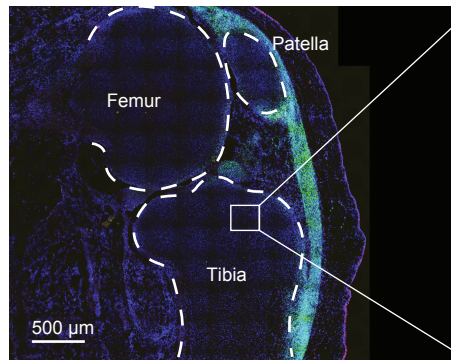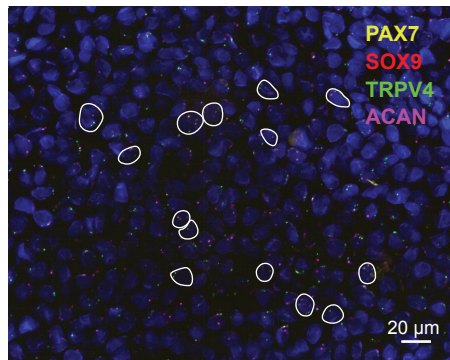

6 PCW Shoulder

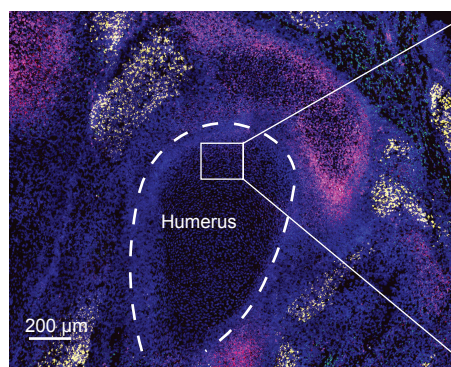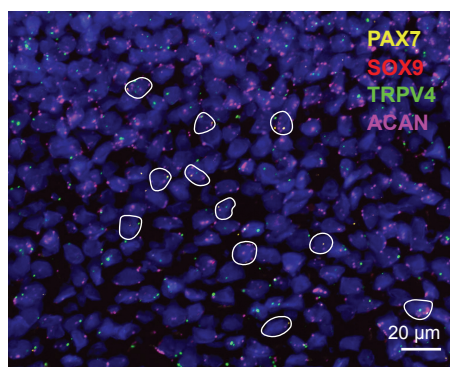**b**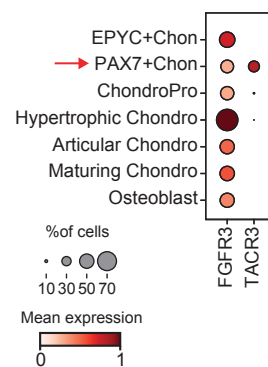**c**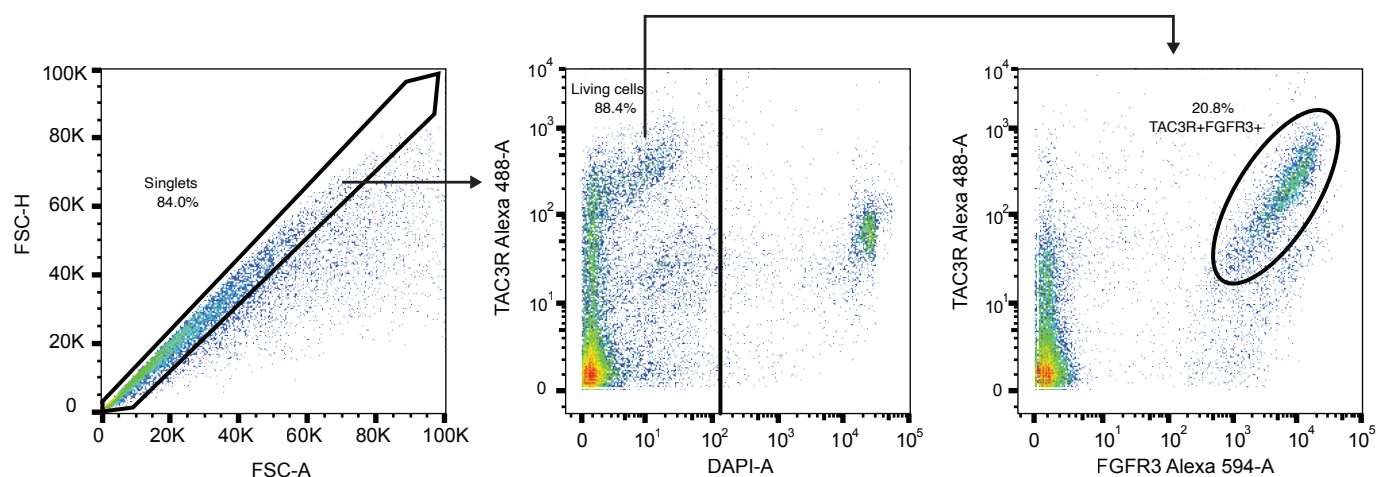**d**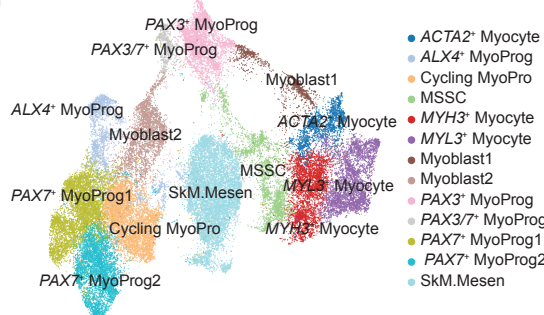

Supplement: Supplementary file 3 — Supplementary Figures [file 41586_2024_8189_MOESM3_ESM.zip › 2023-10-17784B-s3/2023-10-17784B-Supplementary Data Figure 6.pdf]

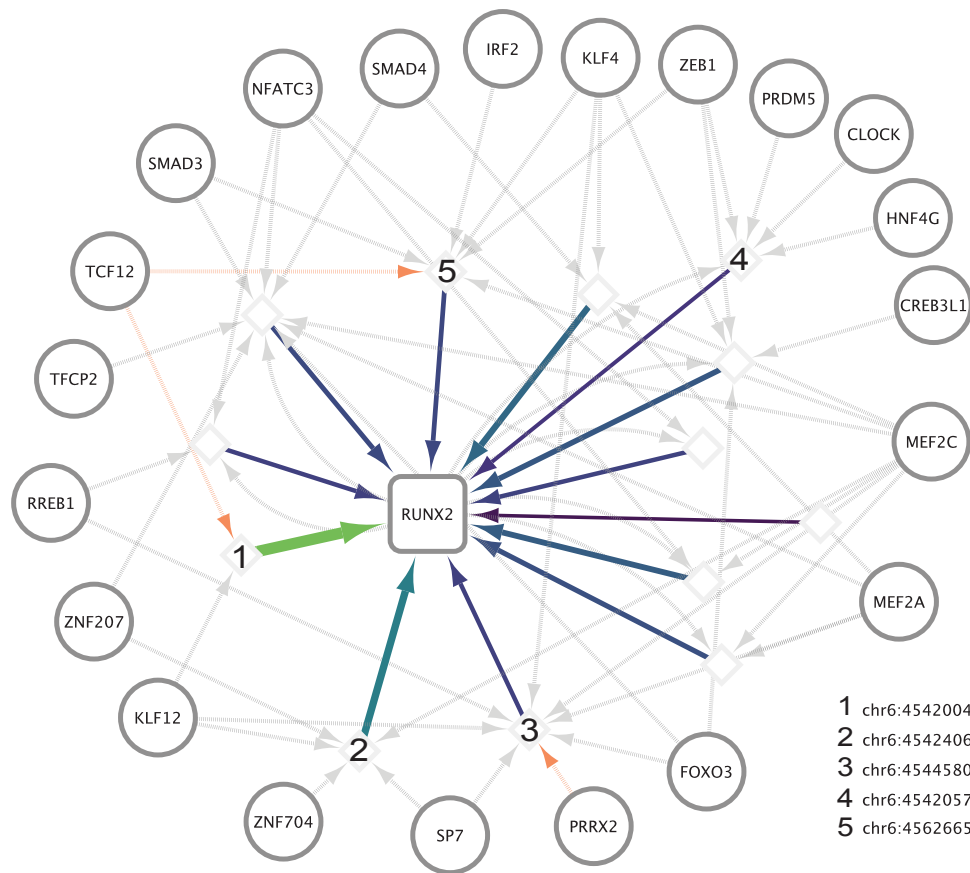

Supplement: Supplementary file 3 — Supplementary Figures [file 41586_2024_8189_MOESM3_ESM.zip › 2023-10-17784B-s3/2023-10-17784B-Supplementary Data Figure 7.pdf]

**a**

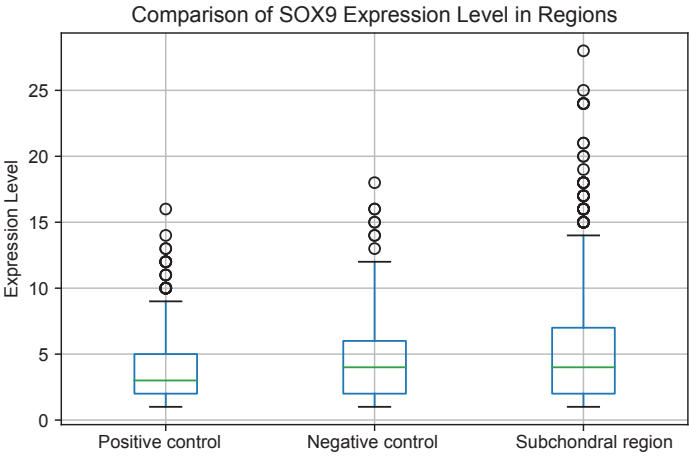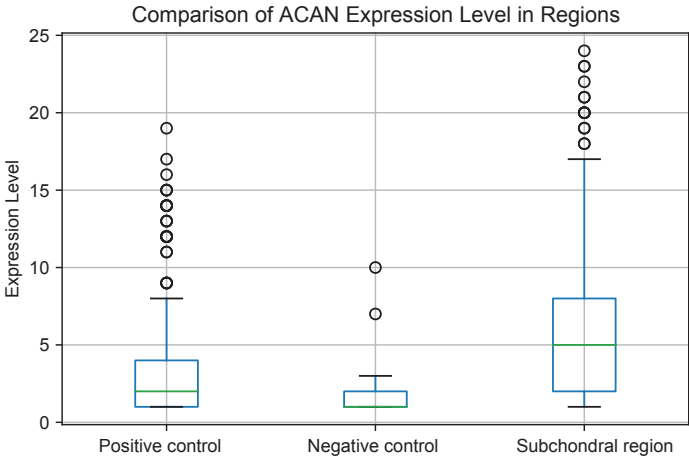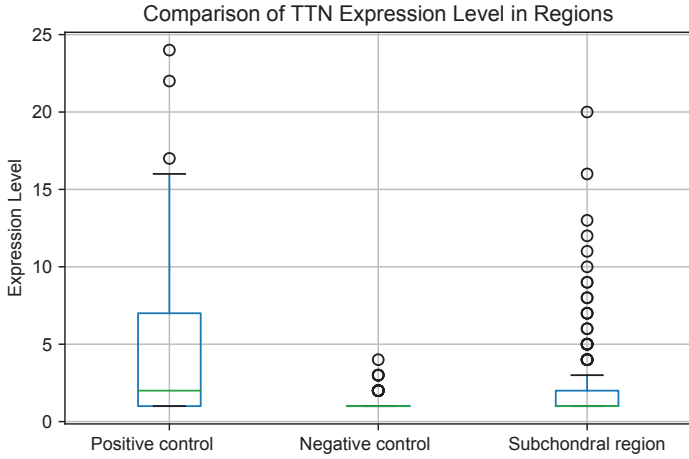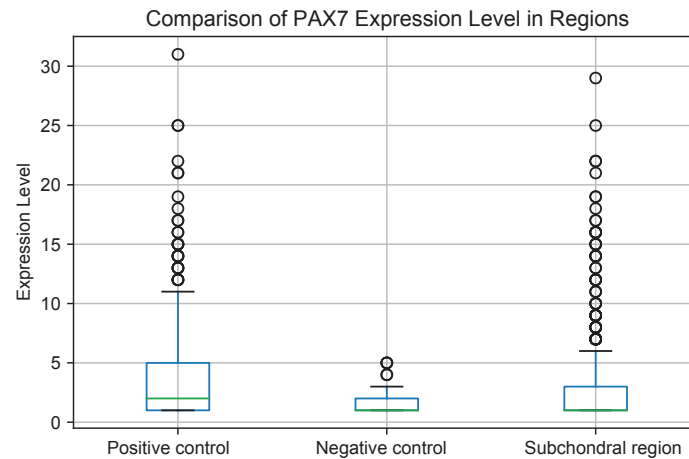

Supplement: Supplementary file 3 — Supplementary Figures [file 41586_2024_8189_MOESM3_ESM.zip › 2023-10-17784B-s3/2023-10-17784B-Supplementary Data Figure 8.pdf]
