# Supplementary material for: A multi-omic atlas of human embryonic skeletal development
Source: Nature. 2024 Nov 20;635(8039):657–67. doi: 10.1038/s41586-024-08189-z (PMC11578895; doi:10.1038/s41586-024-08189-z)
Supplement: Supplementary file 4 — Supplementary Tables [file 41586_2024_8189_MOESM4_ESM.zip › 2023-10-17784B-s4/SuppTables.docx]

**Supplementary Table 1. Samples Overview**

List of donor ID obtained under approvals of REC 96/085 with corresponding anatomical regions, stage (PCW) and number of samples within each region processed across the skeleton. Stage was determined using crown-rump length (CRL) calculations: PCW (days) = 0.9022 × CRL (mm) + 27.372. (See SuppTable_1.xlsx)

**Supplementary Table 2. Droplet recovery information**

Details of each 10X Multiome run including information on target recovery during loading of the 10X lane with droplets, and recovery of droplets following data quality control. (See SuppTable_10.xlsx)

**Supplementary Table 3. Cell cluster marker genes**

Differentially expressed genes (DEGs) for major transcriptomic clusters defined in this study across compartments. (See SuppTable_2.xlsx)

**Supplementary Table 4. ISS Probe panel**

List of RNA probes with gene name and ENSEMBL ID for each gene. Probes were obtained through CARTANA. (See SuppTable_3.xlsx)

**Supplementary Table 5. Craniosynostosis-associated genes**

Curated list of craniosynostosis genes obtained from the online platform Genomics England Panel. (See SuppTable_5.xlsx)

**Supplementary Table 6. Differential gene expression along the trajectories of intramembranous and endochondral osteogenesis**

Differential expression results using a spatial autocorrelation test in monocle3. Columns: gene symbol, module, supermodule, q-value, p-value, morans I, morans test statistic and status. The first sheet contains the filtered DE genes, the second contains results for all genes.

**Supplementary Table 7. Enriched pathways across osteogenesis pseudotime**

Pathways enrichment scored from gene-sets obtained through numerous databases against pseudotime-associated genes within the osteogenesis subcompartment. (See SuppTable_4.xlsx)

**Supplementary Table 8. Genes within each grouped module, and their enrichment for GO terms in chondrocytes, obtained from hotspot analysis**

Details of GO terms, gene names (‘geneID’), q-value and p-value for hotspot.

**Supplementary Table 9. RNAscope probes**

List of probes used for RNAscope experiments and relevant information (See SuppTable_7.xlsx)

**Supplementary Table 10. Genome-wide association studies metadata**

Details of GWAS studies utilised in fGWAS enrichment analysis (See SuppTable_8.xlsx)

**Supplementary Table 11. SCENIC+ results**

TF-enhancer-gene links for osteogenesis, chondrogenesis, fibrogenesis, early joint progenitors, immune and Schwann cells. (See SuppTable_9.xlsx)

**Supplementary Table 12. Drugs with teratogenicity warning**

List of drug names obtained through chEMBL database with black box labels of teratogenicity as a feature. (See SuppTable_6.xlsx)
